# Supplementary figures and images for: Assessment of biomass potentials of microalgal communities in open pond raceways using mass cultivation
Source: PeerJ. 2020 Jul 16;8:e9418. doi: 10.7717/peerj.9418 (PMC7369025; doi:10.7717/peerj.9418)

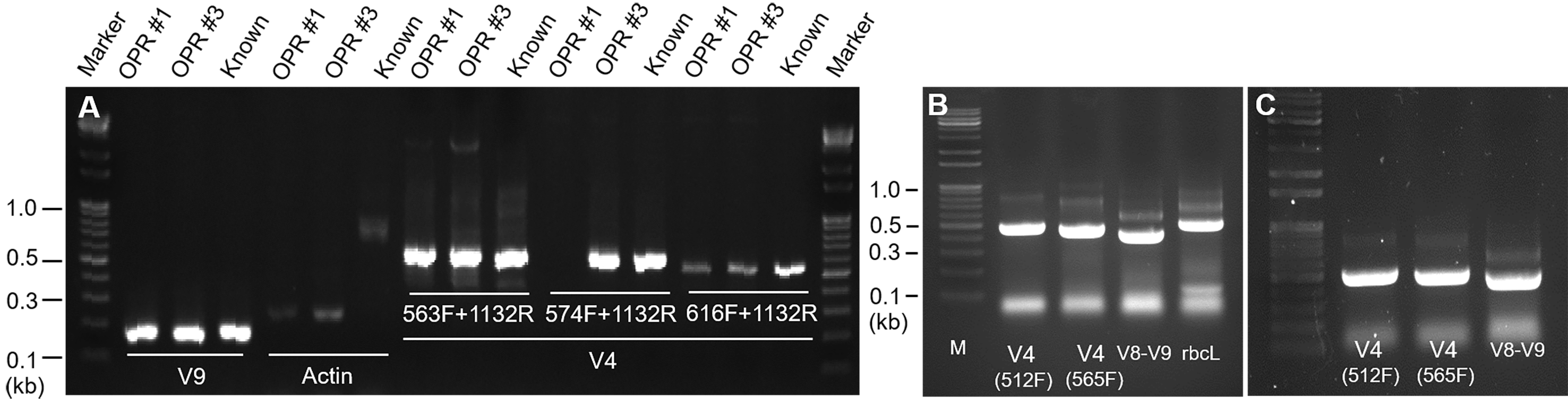

Supplement: Figure S1 — (A) After gDNA isolation, PCR was performed with a primer set, including actin, and V4 (533F and 1132R, 574F and 1132R, and 616F and 1132R) and V9 regions of 18S rDNA. PCR products of actin and the V9 region were <400–500 bp and could not be used in MiSeq. M: 1 kb ladder marker; OPR 1: biomass pooled from OPR 1; OPR 3: biomass pooled from OPR 3; known: Desmodesmus sp. biomass. (B) Comparison of V4 (512F and 1132R, and 512F and 1132R) and V8–V9 regions of 18S rDNA and rbcL in terms of MiSeq performance. As shown, PCR products of V4 and V8–V9 regions were more effective for MiSeq compared to rbcL. (C) Reconfirmation of V4 (512F and 1132R, and 512F and 1132R) and V8–V9 regions of 18S rDNA. M: 1 kb ladder marker. PCR, polymerase chain reaction; gDNA, genomic DNA; rDNA, recombinant DNA; OPR, open raceway pond. [file peerj-08-9418-s001.png]

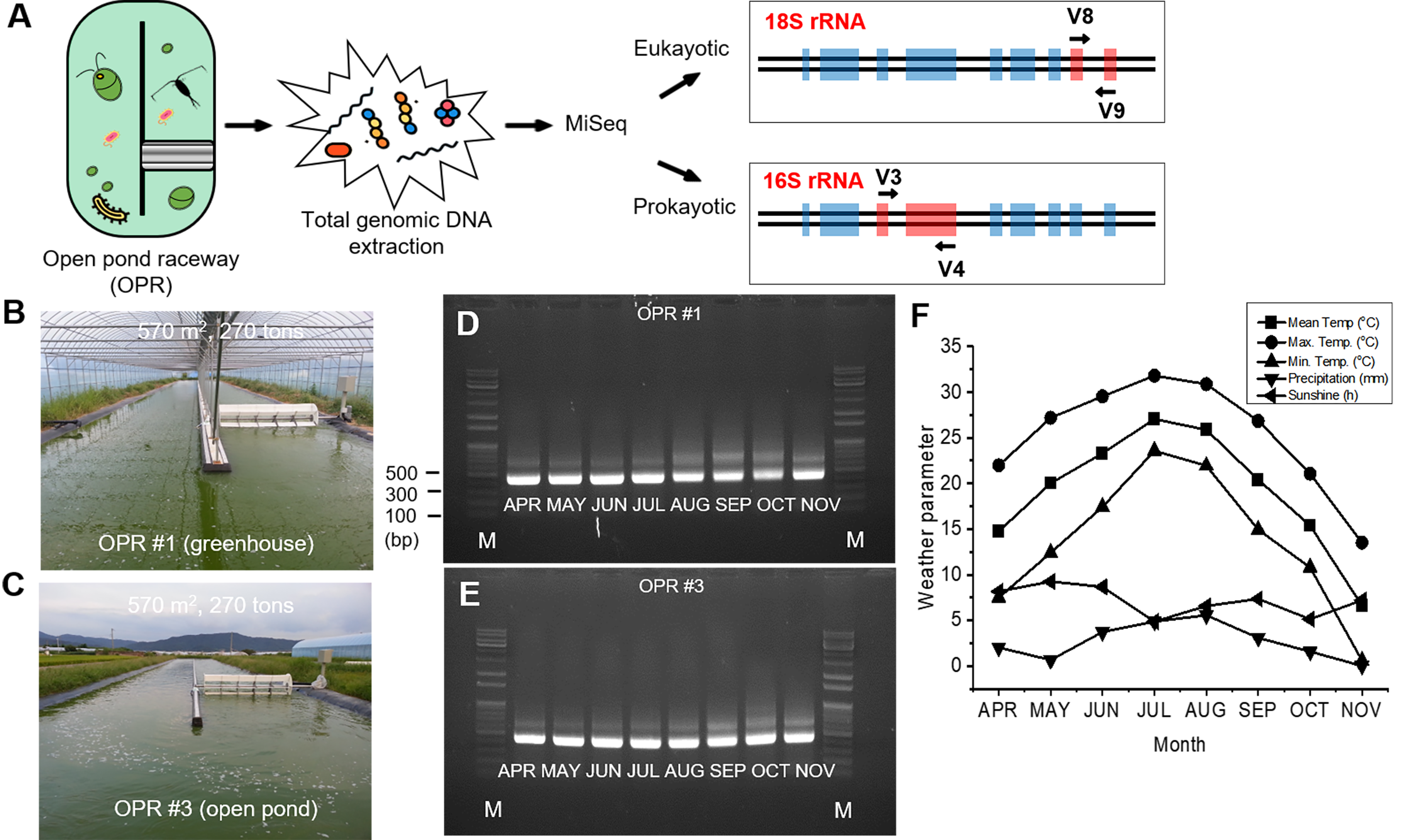

Supplement: Figure S2 — (A) Schematic diagram of MiSeq with biomass harvested from an OPR. Photographs of two mass cultivation systems: (B) OPR 1 and (C) OPR 3. The gDNA was isolated from the pooled biomass from April to November 2017 during mass cultivation in (D) OPR 1 and (E) OPR 3. PCR products using a primer set with the V8–V9 region of 18S rDNA were visualized using 1% agarose gel electrophoresis. (F) Changes in the mean (square), maximum (circle), and minimum (up triangle) temperatures (°C); precipitation (mm; down triangle); and sunshine (h; left triangle) were monitored during the mass cultivation period and reported monthly. OPR, open pond raceway; gDNA, genomic DNA; PCR, polymerase chain reaction; rDNA, recombinant DNA. [file peerj-08-9418-s002.png]

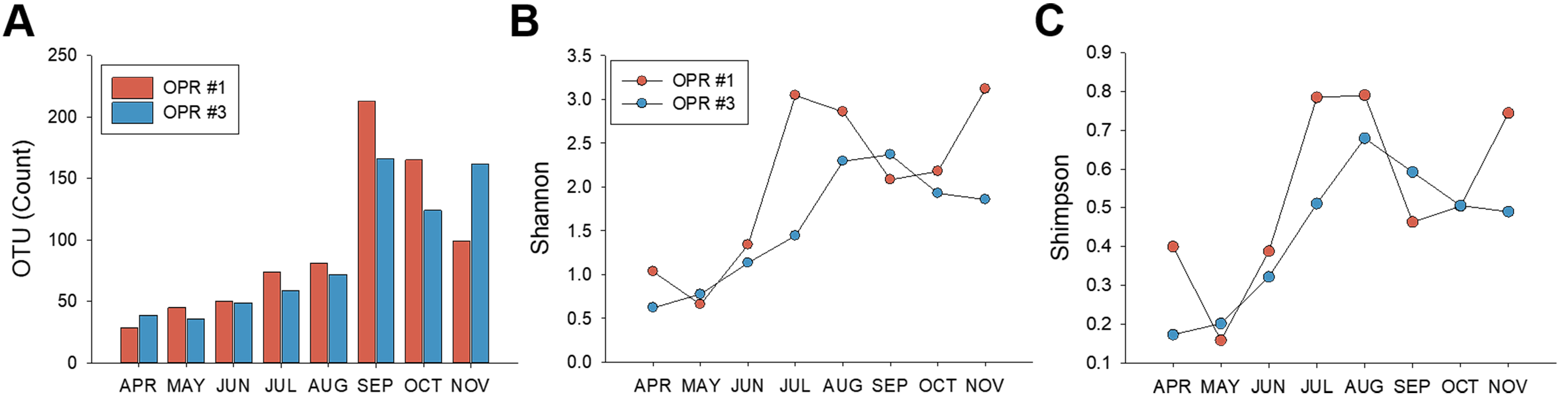

Supplement: Figure S3 — (A) OTU count, (B) Shannon index, and (C) Simpson index from MiSeq results. Light-red bar or circle, OPR 1; light-blue bar or circle, OPR 3. OTU, operational taxonomic unit; OPR, open pond raceway. [file peerj-08-9418-s003.png]

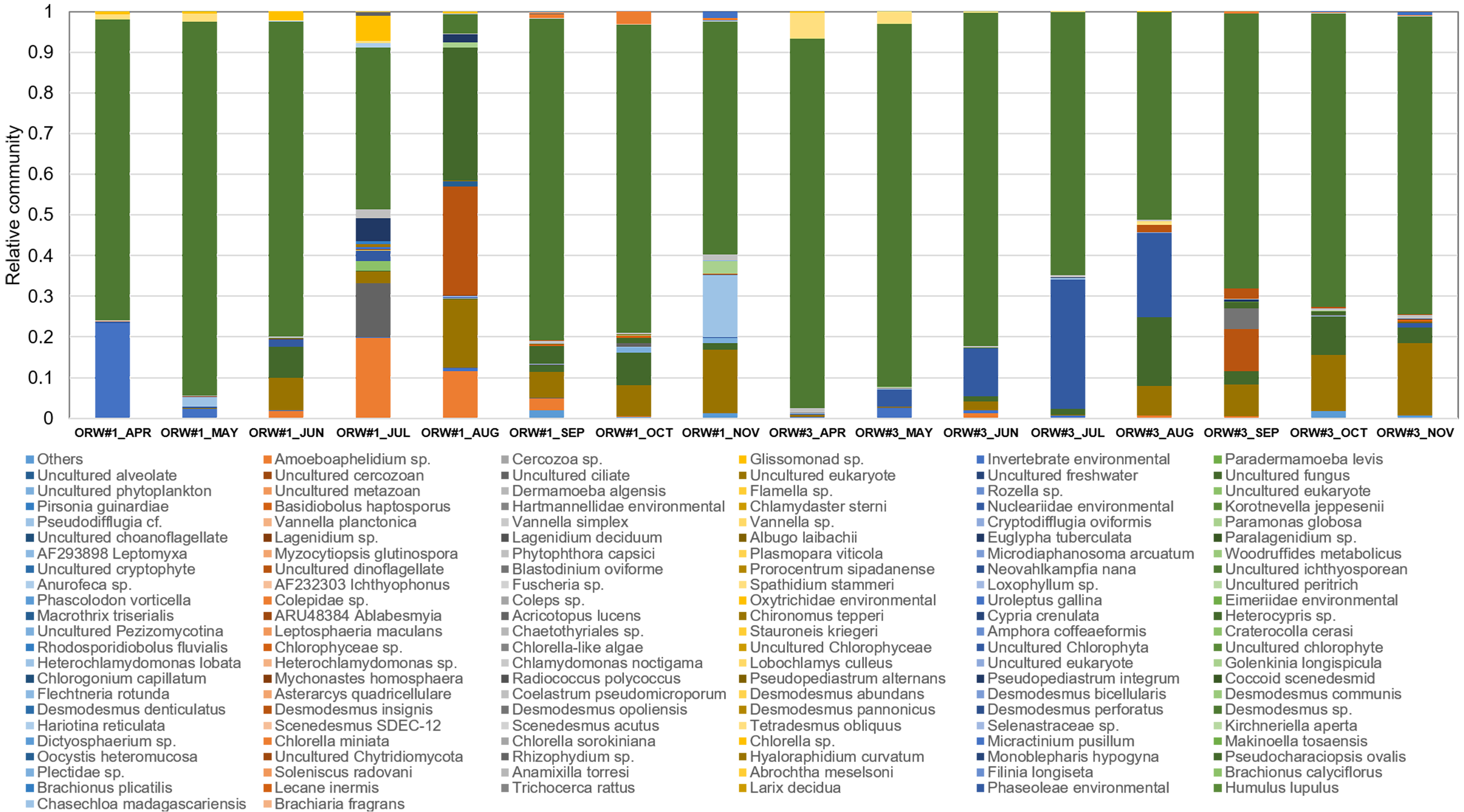

Supplement: Figure S4 [file peerj-08-9418-s004.png]

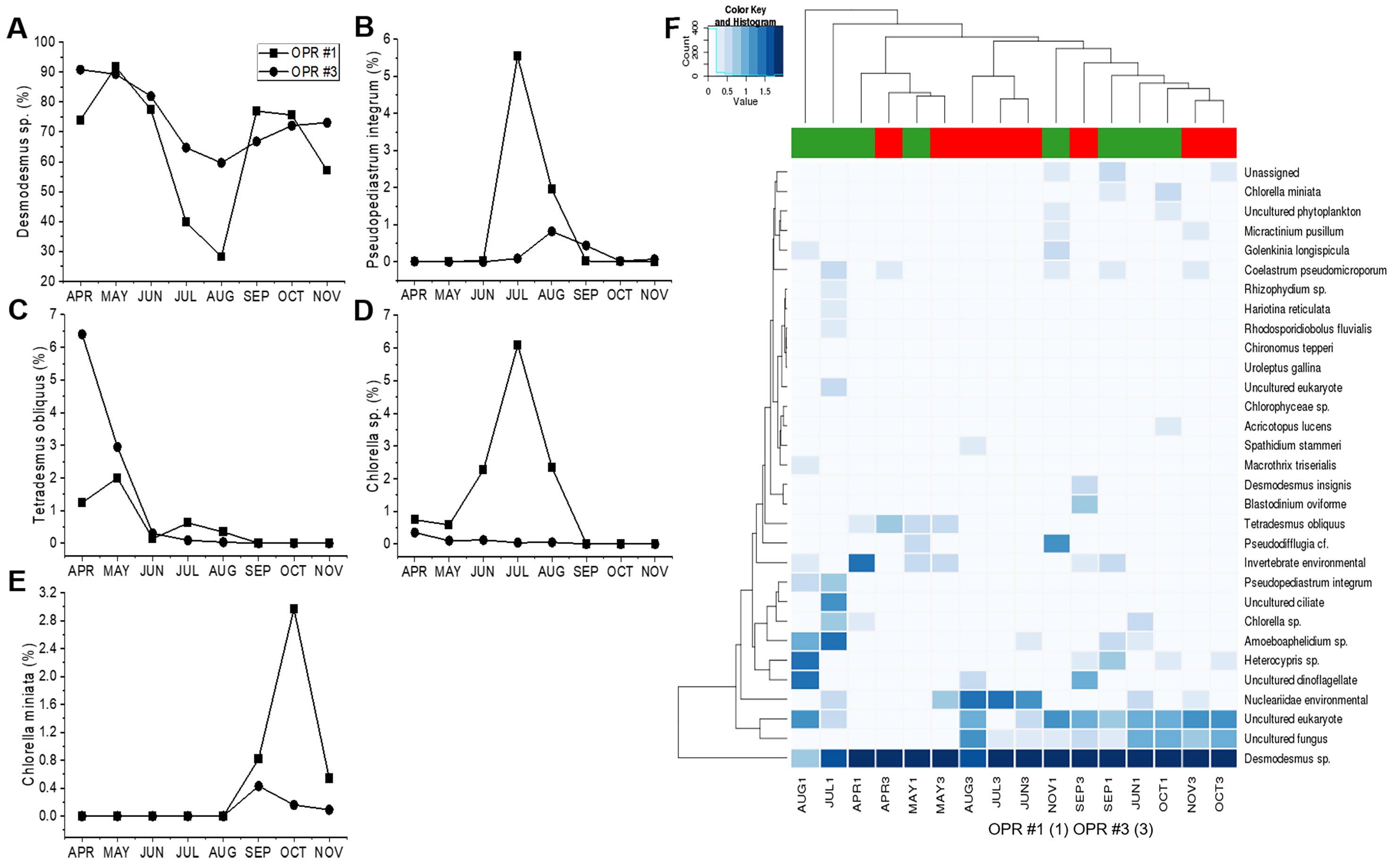

Supplement: Figure S5 — The identified microalgae were (A) Desdodesmus sp., (B) Pseudopediastrum integrum, (C) Tetradesmus obliquus, (D) Chlorella sp., and (E) Chlorella miniate; the dominant microalgal species was Desdodesmus sp.. [file peerj-08-9418-s005.png]

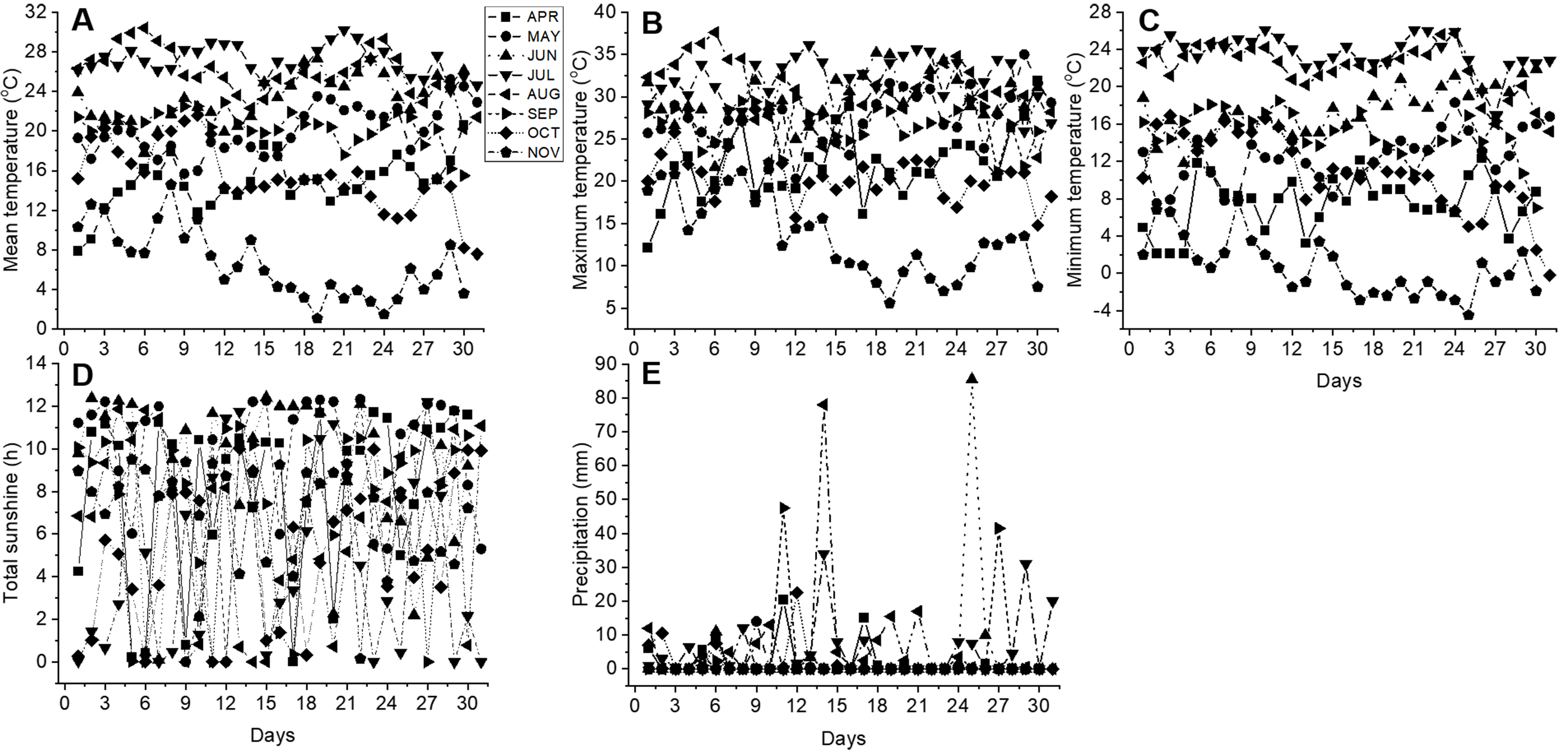

Supplement: Figure S6 — Square, April; circle, May; up triangle, June; down triangle, July; left triangle, August; right triangle, September; diamond, October; pentagon, November. [file peerj-08-9418-s006.png]

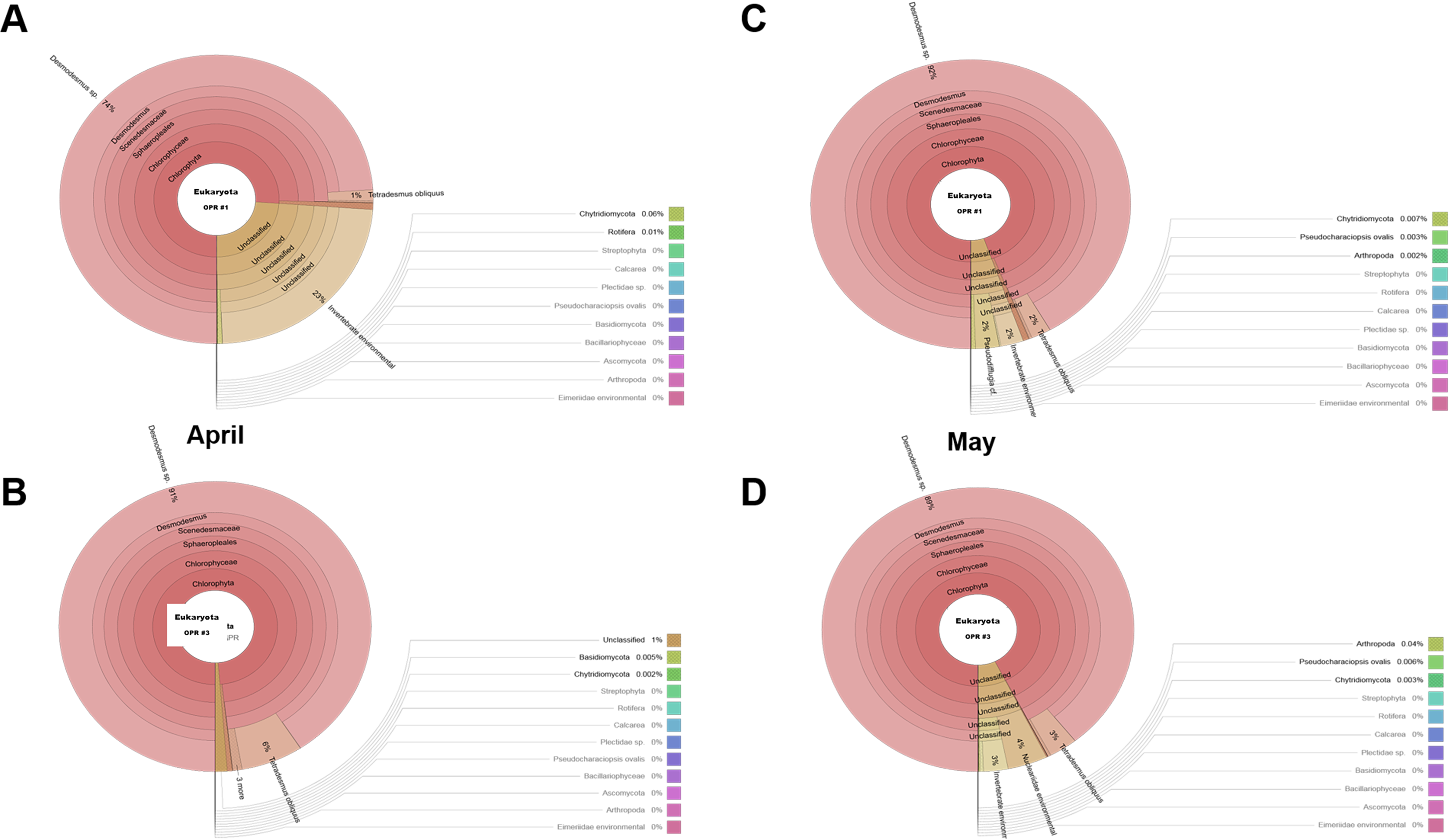

Supplement: Figure S7 — OPR, open pond raceway. [file peerj-08-9418-s007.png]

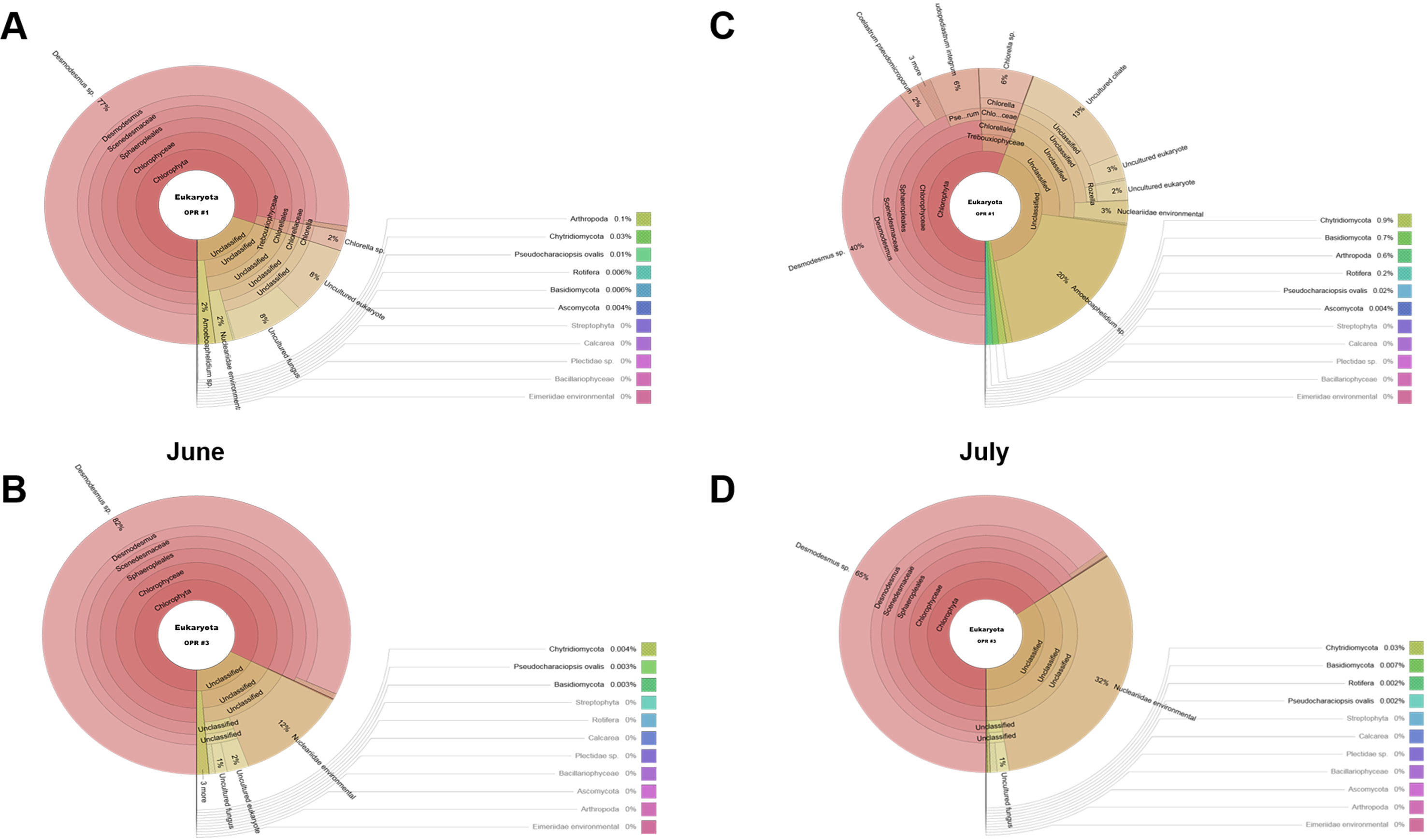

Supplement: Figure S8 — OPR, open pond raceway. [file peerj-08-9418-s008.png]

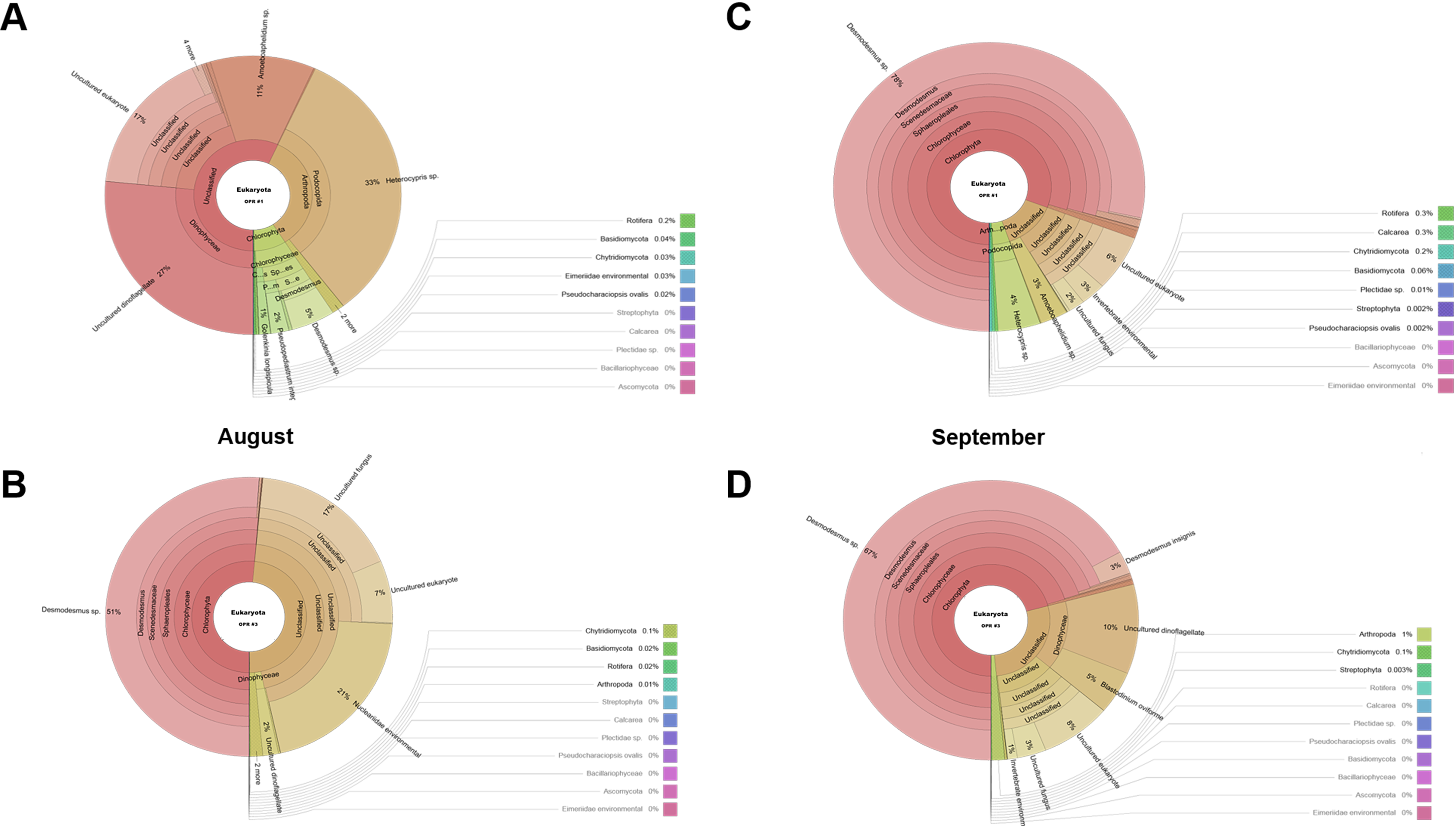

Supplement: Figure S9 — OPR, open pond raceway. [file peerj-08-9418-s009.png]

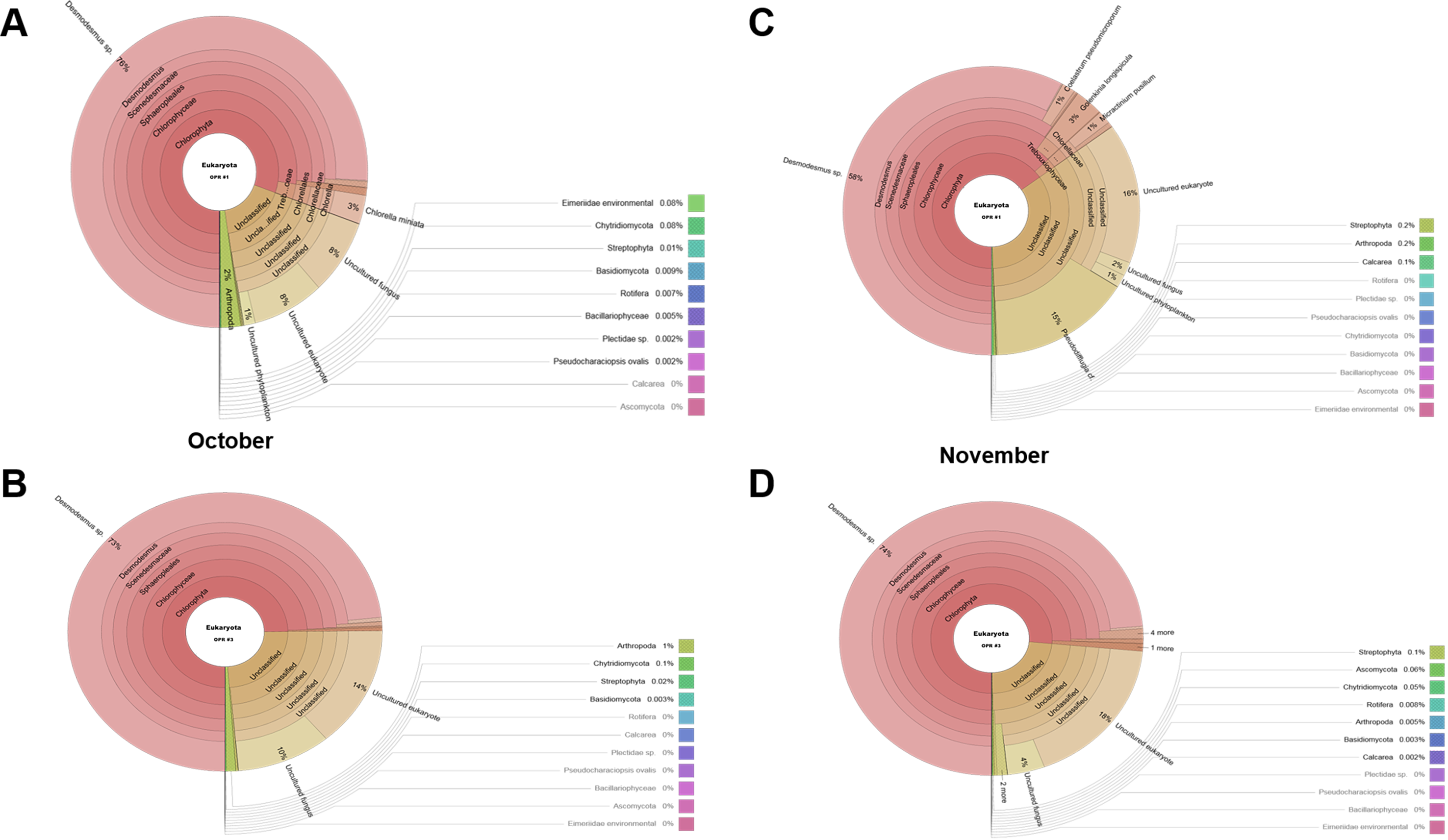

Supplement: Figure S10 — OPR, open pond raceway. [file peerj-08-9418-s010.png]

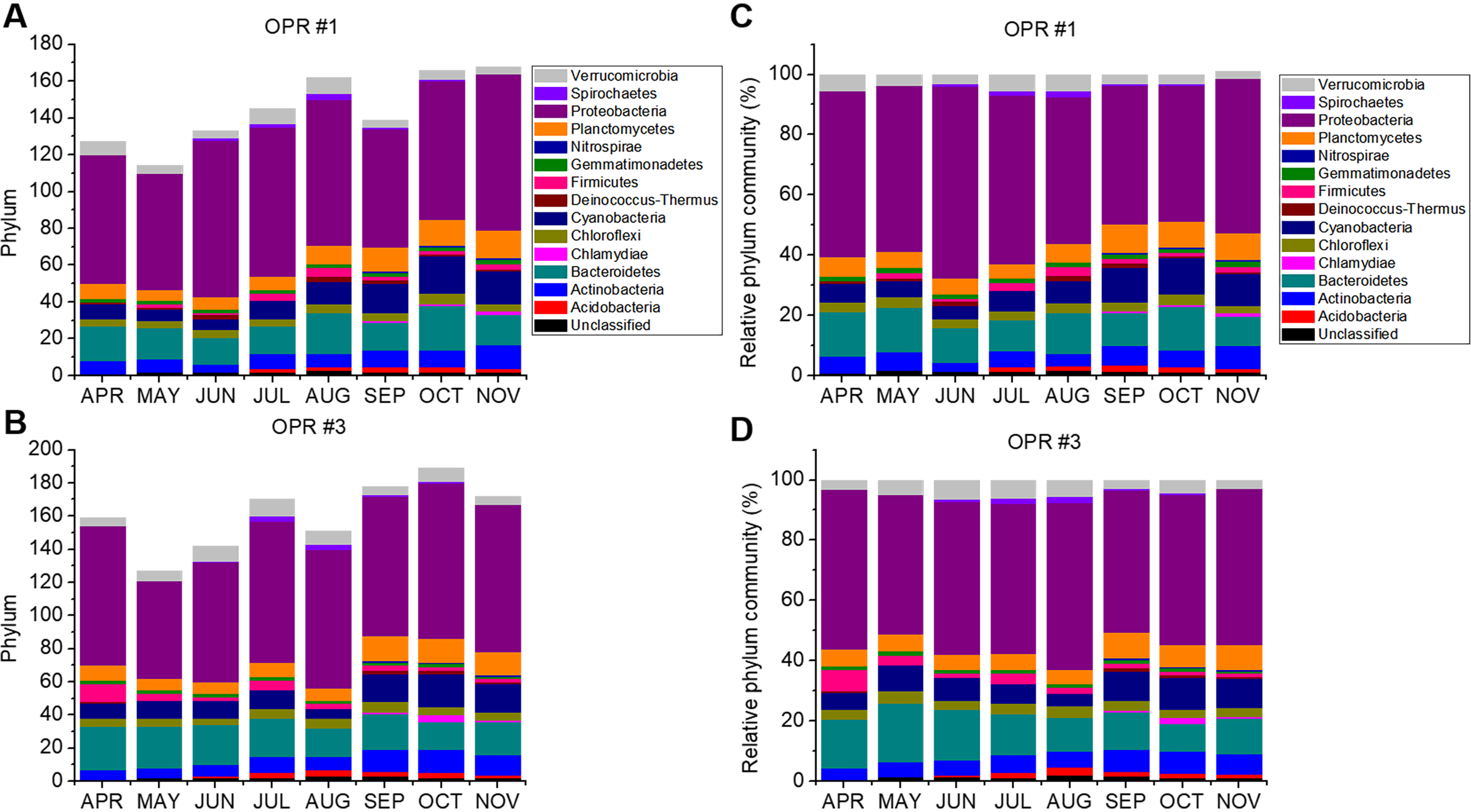

Supplement: Figure S11 — Biodiversity was represented as (A, B) phylum and (C, D) relative phylum community in OPRs 1 and 3. Light gray, Verrucomicrobia; violet, Spirochaetes; purple, Proteobacteria; orange, Planctomycetes; royal, Nitrospirae; olive, Gemmatimonadetes; pink, Fimicutes; wine, Deinococcus; navy, Cyanobacteria; dark yellow, Chloroflexi; magenta, Chlamydiae; dark cyan, Bacterioidetes; blue, Actinobacteria; red, Acidobacteria; black, unclassified. OPR, open pond raceway. [file peerj-08-9418-s011.png]

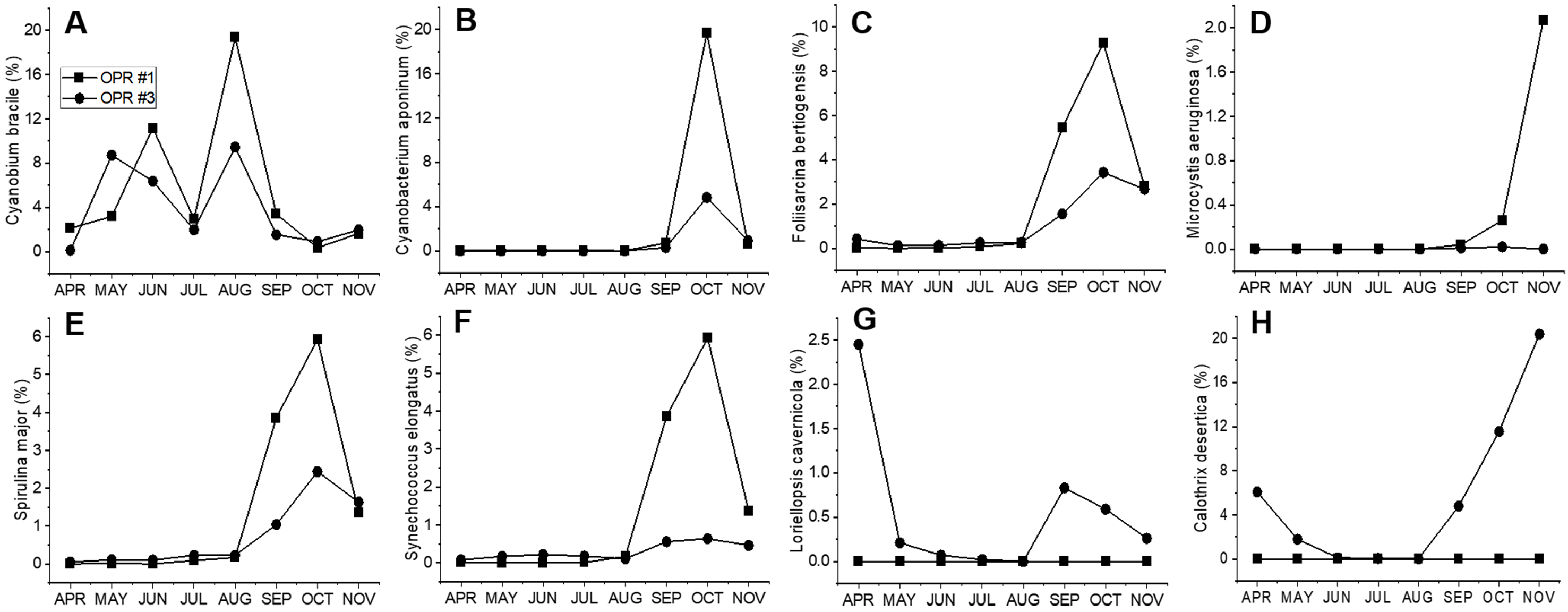

Supplement: Figure S12 — (A) Cyanobium bracile, (B) Cyabobacterium aponium, (C) Foliisarcina bertiogensis, (D) Microcyctis aeruginosa, (E) Spirulina major, (F) Synechococcus elongatus, (G) Loriellopsis cavernicola, and (H) Calothrix desertica. Square, OPR 1; circle, OPR 3. OPR, open pond raceway. [file peerj-08-9418-s012.png]

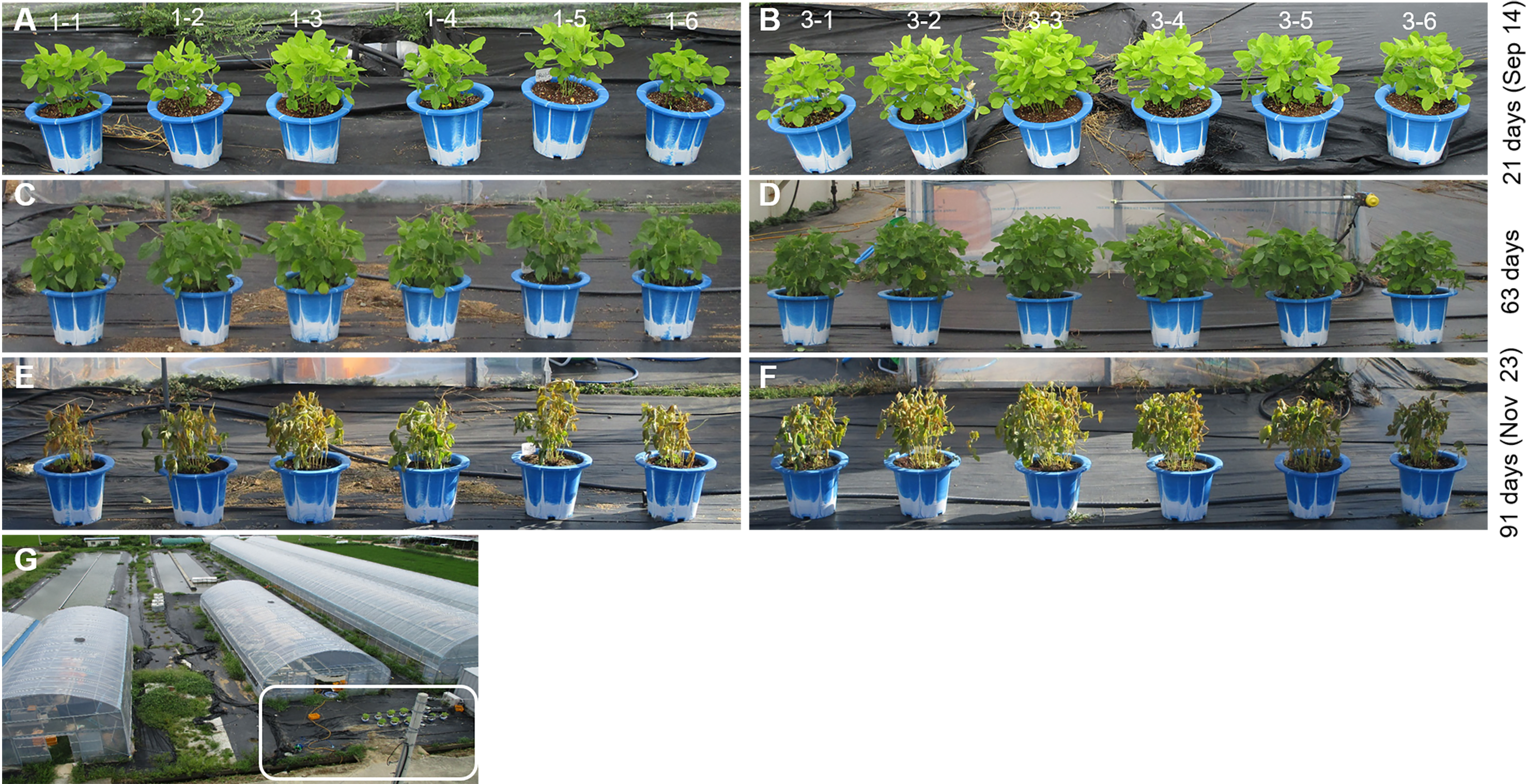

Supplement: Figure S13 — A second biofertilizer experiment was conducted under actual field conditions from August 24 to November 30, 2018. Soybean seeds were grown in potting soil. V2 seedlings were transplanted after 15 days to 30 cm pots (15 seedlings/pot). The plants were grown for 14 weeks and hand-watered, as needed. Environmental adaptation was measured by monitoring the phenotype from the vegetative to the reproductive stage. Phenotypes were photographed at (A, B) 21, (C, D) 63, and (E, F) 91 days in OPRs 1 and 3, respectively. 1–1 and 3–1, soybean plants supplemented with groundwater; 1–2 and 3–2, soybean plants supplemented with eco-sol medium; 1–3 and 3–3, soybean plants supplemented with the clear supernatant from OPRs 1 and 3; 1–4 and 3–4, soybean plants supplemented with microalgal biomasses from OPRs 1 and 3; 1–5 and 3–5, soybean plants supplemented with the cultured solution (supernatant plus microalgal biomasses) from OPRs 1 and 3; 1–6 and 3–6, soybean plants sprayed with the clear supernatant from OPRs 1 and 3. (G) The experiments were performed near the mass cultivation system (boxed in white). OPR, open pond raceway. [file peerj-08-9418-s013.png]

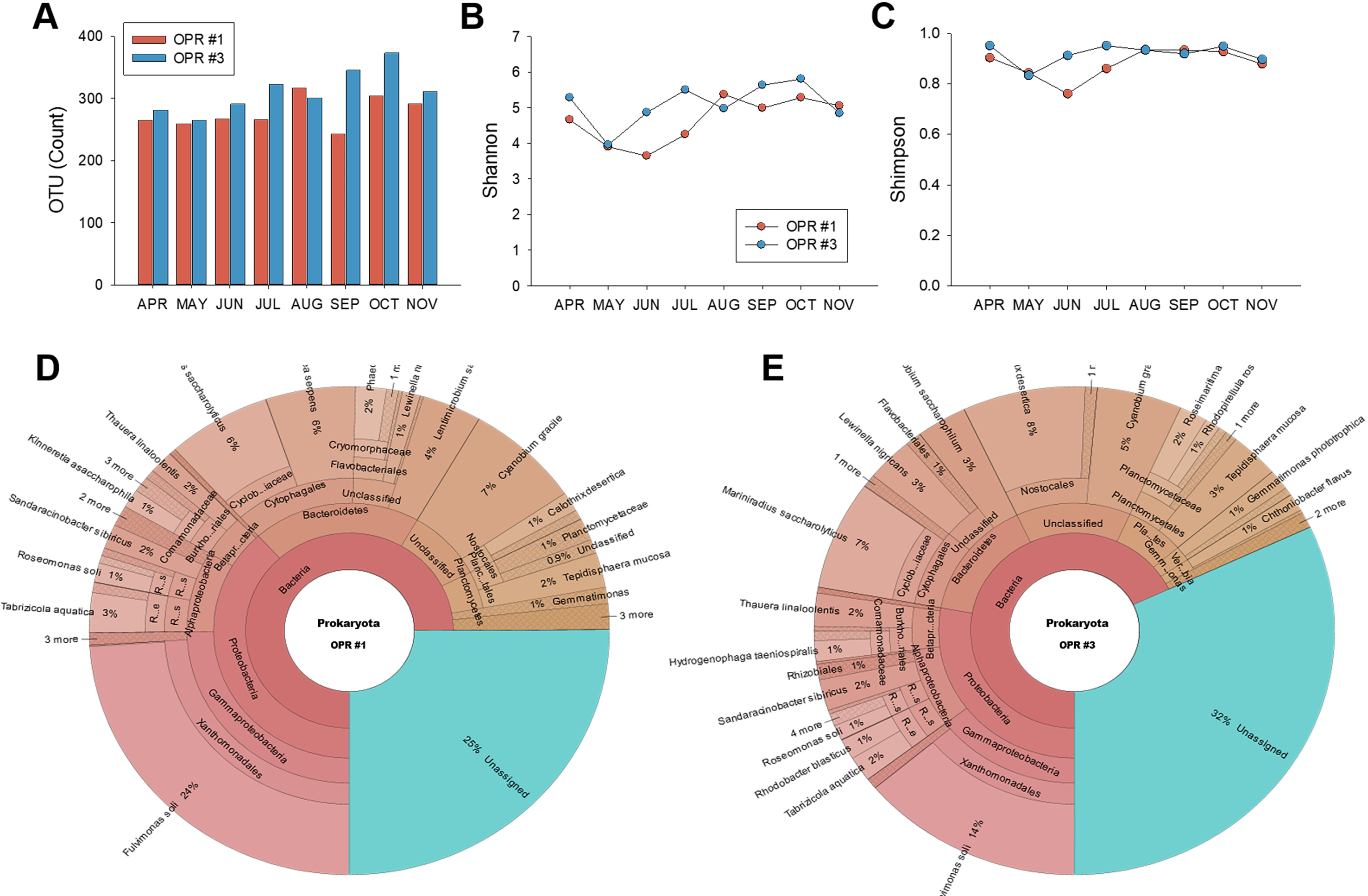

Supplement: Figure S14 — Overview of the MiSeq-based prokaryotic community was represented as the (A) OTU count, (B) Shannon index, and (C) Simpson index. Dark bar or square, OPR 1; black bar or circle, OPR 3. Prokaryotic population richness, including bacteria and cyanobacteria, in (D) OPR 1 and (E) OPR 3. OPR, open pond raceway. [file peerj-08-9418-s014.png]
