# Supplementary material for: Assessment of biomass potentials of microalgal communities in open pond raceways using mass cultivation
Source: PeerJ. 2020 Jul 16;8:e9418. doi: 10.7717/peerj.9418 (PMC7369025; doi:10.7717/peerj.9418)
Supplement: Table S1 [file peerj-08-9418-s015.docx]

Table S1. Oligonucleotide used in this study.

| Primer | Oligonucleotide sequence (5′-3′) | References |
| --- | --- | --- |
| 563F | GCCAGCAVCYGCGGTAAY | *Hugerth et al., 2014* |
| 574F | CGGTAAYTCCAGCTCYAV |  |
| 616F | TTAAARVGYTCGTAGTYG |  |
| 1132R | CCGTCAATTHCTTYAART |  |
| ActF | ATGGTNGGYATGGACCARAA | *Guo et al., 2016* |
| ActR | AAVGTCTCRAACATRATYTGNGTCA |  |
| V9F | CCCTGCCHTTTGTACACAC |  |
| V9R | CCTTCYGCAGGTTCACCTAC |  |
| 512F | ATTCCAGCTCCAATAGCG | *Zimmermann et al., 2011* |
| 978R | GACTACGATGGTATCTAATC |  |
| 565F | CCAGCASCYGCGGTAATTCC | *Bradley et al., 2016* |
| 981R | ACTTTCGTTCTTGAT |  |
| 1422F | ATAACAGGTCTGTGATGCCCT |  |
| 1510R | CCTTCYGCAGGTTCACCTAC |  |
| 192F | GGTACTTGGACAACWGTWTGGAC | *Hadi et al., 2016* |
| 657R | GAAACGGTCTCKCCARCGCAT |  |
| Mi512F | TCGTCGGCAGCGTC**AGATGTGTATAAGAGACAG**ATTCCAGCTCCAATAGCG | This study |
| Mi978R | GTCTCGTGGGCTCGG**AGATGTGTATAAGAGACAG**GACTACGATGGTATCTAATC |  |
| Mi565F | TCGTCGGCAGCGTC**AGATGTGTATAAGAGACAG**CCAGCASCYGCGGTAATTCC | This study |
| Mi981R | GTCTCGTGGGCTCGG**AGATGTGTATAAGAGACAG**ACTTTCGTTCTTGAT |  |
| Mi1422F | TCGTCGGCAGCGTC**AGATGTGTATAAGAGACAG**ATAACAGGTCTGTGATGCCCT | This study |
| Mi1510R | GTCTCGTGGGCTCGG**AGATGTGTATAAGAGACAG**CCTTCYGCAGGTTCACCTAC |  |
| Mi192F | TCGTCGGCAGCGTC**AGATGTGTATAAGAGACAG**GGTACTTGGACAACWGTWTGGAC | This study |
| Mi657R | GTCTCGTGGGCTCGG**AGATGTGTATAAGAGACAG**GAAACGGTCTCKCCARCGCAT |  |

The oligonucleotides of the pre-adapter and sequencing are indicated by the underlined and bolded sequences above, respectively.
