# Supplementary material for: Assessment of biomass potentials of microalgal communities in open pond raceways using mass cultivation
Source: PeerJ. 2020 Jul 16;8:e9418. doi: 10.7717/peerj.9418 (PMC7369025; doi:10.7717/peerj.9418)
Supplement: Table S2 [file peerj-08-9418-s016.docx]

Table S2. Establishment of algae-based MiSeq platform using different primer sets.

| Primer |  | Query | | | | Subject | | | | | Score | | Identities | | | Gaps | | Strand | | Taxonomy | |
| --- | --- | --- | --- | --- | --- | --- | --- | --- | --- | --- | --- | --- | --- | --- | --- | --- | --- | --- | --- | --- | --- |
|  | Accession | Q_  Length | Q_  Start | Q_  End | Q_  Cov. | | S_  Length | S_  Start | S_  End | S_  Cov. | Bit | E-Value | | Match | Pct.  (%) | Match | Pct.  (%) | Query | Subject | | Species |
| V8-V9 | |  |  |  |  | |  |  |  |  |  |  | |  |  |  |  |  |  | |  |
|  | denovo0 | 369 | 1 | 369 | 100 | | 2408 | 1374 | 1742 | 15.32 | 676 | 0 | | 368/369 | 99 | 0/369 | 0 | Plus | Plus | | *Pseudopediastrum integrum* |
|  | denovo1 | 369 | 1 | 369 | 100 | | 1792 | 1419 | 1787 | 20.59 | 682 | 0 | | 369/369 | 100 | 0/369 | 0 | Plus | Plus | | *Tetradesmus obliquus* |
|  | denovo2 | 369 | 1 | 369 | 100 | | 1304 | 5 | 373 | 28.29 | 676 | 0 | | 368/369 | 99 | 0/369 | 0 | Plus | Plus | | *Chlamydomonadaceae* sp. |
|  | denovo3 | 369 | 1 | 369 | 100 | | 2945 | 1806 | 2174 | 12.52 | 676 | 0 | | 368/369 | 99 | 0/369 | 0 | Plus | Plus | | *Desmodesmus* sp. |
|  | denovo4 | 369 | 1 | 369 | 100 | | 3573 | 2709 | 3077 | 10.32 | 638 | 4E-178 | | 361/369 | 97 | 0/369 | 0 | Plus | Plus | | *Chlamydomonad* sp. |
|  | denovo5 | 369 | 1 | 369 | 100 | | 1793 | 1420 | 1788 | 20.58 | 682 | 0 | | 369/369 | 100 | 0/369 | 0 | Plus | Plus | | *Chlorella* sp. |
| V4 565F-981R | |  |  |  |  | |  |  |  |  |  |  | |  |  |  |  |  |  | |  |
|  | denovo0 | 416 | 1 | 416 | 100 | | 1765 | 544 | 959 | 23.56 | 763 | 0 | | 415/416 | 99 | 0/416 | 0 | Plus | Plus | | *Pseudopediastrum* sp. |
|  | denovo1 | 416 | 1 | 416 | 100 | | 1766 | 544 | 959 | 23.55 | 763 | 0 | | 415/416 | 99 | 0/416 | 0 | Plus | Plus | | *Chlamydomonas* sp. |
|  | denovo2 | 415 | 1 | 415 | 100 | | 1001 | 370 | 784 | 41.45 | 761 | 0 | | 414/415 | 99 | 0/415 | 0 | Plus | Plus | | *Desmodesmus subspicatus* |
|  | denovo3 | 415 | 1 | 415 | 100 | | 1687 | 511 | 925 | 24.59 | 745 | 0 | | 411/415 | 99 | 0/415 | 0 | Plus | Plus | | *Chlamydomonad* sp. |
|  | denovo4 | 415 | 1 | 415 | 100 | | 1672 | 470 | 884 | 24.82 | 767 | 0 | | 415/415 | 100 | 0/415 | 0 | Plus | Plus | | *Chlorella* sp. |
|  | denovo5 | 415 | 1 | 415 | 100 | | 1079 | 513 | 927 | 38.46 | 761 | 0 | | 414/415 | 99 | 0/415 | 0 | Plus | Plus | | Uncultured eukaryote |
| V4 512F-978R | |  |  |  |  | |  |  |  |  |  |  | |  |  |  |  |  |  | |  |
|  | denovo0 | 436 | 1 | 436 | 100 | | 1765 | 559 | 994 | 24.70 | 800 | 0 | | 435/436 | 99 | 0/436 | 0 | Plus | Plus | | *Pseudopediastrum* sp. |
|  | denovo1 | 436 | 1 | 436 | 100 | | 1766 | 559 | 994 | 24.68 | 800 | 0 | | 435/436 | 99 | 0/436 | 0 | Plus | Plus | | *Chlamydomonas* sp. |
|  | denovo2 | 435 | 1 | 435 | 100 | | 1230 | 14 | 448 | 35.36 | 798 | 0 | | 434/435 | 99 | 0/435 | 0 | Plus | Plus | | *Desmodesmus abundans* |
|  | denovo3 | 435 | 1 | 435 | 100 | | 1687 | 526 | 960 | 25.78 | 793 | 0 | | 433/435 | 99 | 0/435 | 0 | Plus | Plus | | *Chlamydomonad* sp. |
|  | denovo4 | 435 | 1 | 429 | 98.6 | | 1771 | 562 | 990 | 24.22 | 793 | 0 | | 429/429 | 100 | 0/429 | 0 | Plus | Plus | | *Chlorella vulgaris* |
|  | denovo5 | 435 | 1 | 435 | 100 | | 2473 | 538 | 975 | 17.71 | 717 | 0 | | 423/439 | 96 | 5/439 | 1 | Plus | Plus | | *Chlorococcum* sp. |
