# Supplementary material for: Assessment of biomass potentials of microalgal communities in open pond raceways using mass cultivation
Source: PeerJ. 2020 Jul 16;8:e9418. doi: 10.7717/peerj.9418 (PMC7369025; doi:10.7717/peerj.9418)
Supplement: Table S3 [file peerj-08-9418-s017.docx]

Table S3. Algae identification using different primer sets.

|  | Original identification | Alternative identification | |
| --- | --- | --- | --- |
| V8-V9 |  |  | |
| \| denovo0 \| \| --- \| \| denovo1 \| \| denovo2 \| \| denovo3 \| \| denovo4 \| \| denovo5 \| | *Pseudopediastrum integrum*  *Tetradesmus obliquus*  *Chlamydomonadaceae* sp.  *Desmodesmus* sp.  *Chlamydomonad* sp.  *Chlorella* sp. | *Pseudopediastrum* sp. KNUA039  *Acutodesmus* sp. KNUA038, *Scenedesmus* *obliquus* KNUA040  *Chlamydomonas* sp. KNUA040  *Desmodesmus* sp. KNUA024  *Chlamydomonad* sp. KNUA023  *Micractinium* sp. KNUA034, *Micractinium* sp. KNUA029, *Micractinium* sp. KNUA036, *Chlorella* sp. KNUA027 | |
| V4 (565F-981R) |  |  | |
| \| denovo0 \| \| --- \| \| denovo1 \| \| denovo2 \| \| denovo3 \| \| denovo4 \| \| denovo5 \| | *Pseudopediastrum* sp.  *Chlamydomonas* sp.  *Desmodesmus subspicatus*  *Chlamydomonad* sp.  *Chlorella* sp.  Uncultured eukaryote | *Pseudopediastrum* sp. KNUA039  *Chlamydomonas* sp. KNUA040  *Scenedesmus* sp.  *Chlamydomonad* sp. KNUA023  *Micractinium* sp.  *Scenedesmus* sp. *Chlorella* sp. | |
| V4 (512F-978R) |  |  |  |
| denovo0 | *Pseudopediastrum* sp. | *Pseudopediastrum* sp. KNUA039 | |
| denovo1 | *Chlamydomonas* sp. | *Chlamydomonas* sp. KNUA040 | |
| denovo2 | *Desmodesmus abundans* | *Desmodesmus abundans* KNUA024 | |
| denovo3 | *Chlamydomonad* sp. | *Chlamydomonad* sp. KNUA023 | |
| denovo4 | *Chlorella vulgaris* | *Chlorella vulgaris* KNUA027 | |
| denovo5 | *Chlorococcum* sp. | *Pseudopediastrum* sp. KNUA039, *Scenedesmus* sp. | |
