# Supplementary material for: Assessment of biomass potentials of microalgal communities in open pond raceways using mass cultivation
Source: PeerJ. 2020 Jul 16;8:e9418. doi: 10.7717/peerj.9418 (PMC7369025; doi:10.7717/peerj.9418)
Supplement: Data S3 [file peerj-08-9418-s020.zip › Krona/OPR#1/OPR#1_APR.html]

Javascript must be enabled to view this page.

magnitude
 99.9999999999796
 99.960708298387
 23.7819572506237
 .0104777870914
 .0104777870914
 .0104777870914
 .0104777870914
 .0104777870914
 23.7714794635323
 23.7243294216211
 23.2580678960556
 23.2580678960556
 0
 0
 23.2344928751
 0
 0
 0
 0
 .0130972338642
 0
 .0104777870914
 0
 0
 0
 0
 0
 0
 0
 0
 0
 0
 0
 0
 0
 0
 0
 0
 0
 0
 0
 0
 0
 .458403185247
 .458403185247
 .458403185247
 0
 0
 0
 .00785834031852
 .00785834031852
 .00785834031852
 0
 0
 0
 0
 0
 0
 0
 0
 0
 0
 0
 0
 0
 0
 0
 0
 0
 0
 0
 0
 0
 0
 0
 0
 0
 .0183361274099
 .0183361274099
 .0183361274099
 0
 .0183361274099
 0
 0
 0
 0
 0
 0
 0
 .0288139145013
 .0288139145013
 .0288139145013
 .0288139145013
 0
 0
 0
 0
 0
 0
 0
 0
 0
 0
 0
 0
 0
 0
 0
 0
 0
 0
 0
 0
 0
 0
 0
 0
 0
 0
 0
 0
 0
 0
 0
 0
 0
 0
 0
 0
 0
 0
 0
 0
 0
 0
 0
 0
 0
 0
 0
 0
 0
 0
 0
 0
 0
 0
 0
 0
 0
 0
 0
 0
 0
 0
 0
 0
 0
 0
 0
 0
 0
 0
 0
 0
 0
 0
 0
 0
 0
 0
 0
 0
 0
 0
 0
 0
 0
 0
 0
 0
 0
 0
 0
 0
 0
 0
 0
 0
 0
 0
 0
 0
 0
 0
 0
 0
 0
 0
 0
 .0104777870914
 .0104777870914
 0
 0
 0
 0
 .0104777870914
 0
 0
 0
 0
 0
 0
 0
 .0104777870914
 .0104777870914
 .0104777870914
 0
 0
 0
 0
 0
 0
 0
 0
 0
 0
 0
 0
 0
 0
 0
 0
 0
 0
 0
 0
 0
 0
 0
 0
 0
 0
 0
 .0628667225482
 0
 0
 0
 0
 0
 0
 0
 0
 0
 0
 0
 0
 0
 .0628667225482
 .0628667225482
 .0628667225482
 .0628667225482
 .0628667225482
 76.1054065381237
 75.3667225481829
 0
 0
 0
 0
 0
 0
 0
 0
 0
 0
 0
 0
 0
 0
 0
 0
 75.3667225481829
 .00523889354568
 .00523889354568
 0
 .00523889354568
 75.3352891869088
 0
 0
 .149308466052
 .149308466052
 73.8893545683
 0
 0
 0
 0
 0
 0
 0
 0
 73.8893545683
 .015716680637
 .015716680637
 .0366722548198
 0
 .0366722548198
 1.2442372171
 1.2442372171
 0
 0
 0
 0
 0
 0
 0
 0
 .0261944677284
 0
 0
 .0261944677284
 .0261944677284
 0
 0
 0
 0
 0
 .736064543168
 .736064543168
 .736064543168
 .736064543168
 0
 0
 .736064543168
 0
 0
 0
 0
 0
 0
 0
 0
 0
 0
 0
 .00261944677284
 .00261944677284
 .00261944677284
 .00261944677284
 .00261944677284
 0
 0
 0
 0
 0
 0
 0
 0
 0
 0
 0
 0
 0
 0
 0
 0
 0
 0
 0
 0
 0
 0
 0
 0
 0
 0
 0
 0
 0
 0
 0
 0
 0
 0
 0
 0
 0
 0
 0
 0
 0
 0
 0
 0
 0
 0
 0
 0
 0
 0
 0
 0
 0
 0
 0
 0
 0
 0
 0
 0
 0
 0
 0
 0
 0
 0
 0
 0
 .0392917015926
 .0392917015926
 .0392917015926
 .0392917015926
 .0392917015926
 .0392917015926
 .0392917015926
