# Supplementary material for: Assessment of biomass potentials of microalgal communities in open pond raceways using mass cultivation
Source: PeerJ. 2020 Jul 16;8:e9418. doi: 10.7717/peerj.9418 (PMC7369025; doi:10.7717/peerj.9418)
Supplement: Data S3 [file peerj-08-9418-s020.zip › Krona/OPR#1/OPR#1_AUG.html]

Javascript must be enabled to view this page.

magnitude
 99.999999999926
 99.82130584185
 56.7972508590641
 11.3780068729
 11.3780068729
 11.3780068729
 11.3780068729
 11.3780068729
 18.7044673539053
 18.2164948453142
 17.9518900343173
 17.7044673539049
 0
 .0446735395189
 .742268041237
 0
 0
 0
 .127147766323
 16.6013745704
 0
 .189003436426
 0
 0
 0
 .171821305842
 .171821305842
 .00343642611684
 .00343642611684
 0
 0
 .07216494845358
 .0652920962199
 .00687285223368
 0
 0
 0
 0
 0
 0
 0
 0
 0
 .26116838488
 .26116838488
 .26116838488
 0
 0
 0
 0
 0
 0
 .00343642611684
 .00343642611684
 .00343642611684
 0
 0
 0
 0
 0
 0
 .323024054983
 .323024054983
 .323024054983
 .323024054983
 0
 0
 0
 0
 .0171821305842
 .0171821305842
 .0171821305842
 .0171821305842
 0
 0
 0
 0
 .123711340206
 .123711340206
 .123711340206
 0
 .123711340206
 0
 0
 0
 .0240549828179
 .0240549828179
 .0240549828179
 .0240549828179
 0
 0
 0
 0
 0
 0
 0
 0
 0
 0
 0
 0
 0
 0
 0
 0
 0
 0
 0
 0
 .0412371134021
 .0412371134021
 .0412371134021
 .0412371134021
 .0412371134021
 26.5498281786505
 0
 0
 0
 0
 .0103092783505
 .0103092783505
 .0103092783505
 .0103092783505
 26.5395189003
 26.5395189003
 26.5395189003
 26.5395189003
 0
 0
 0
 0
 0
 0
 0
 0
 0
 0
 0
 0
 0
 0
 .1237113402062
 .106529209622
 .106529209622
 .106529209622
 .106529209622
 0
 0
 0
 .0171821305842
 .0171821305842
 .0171821305842
 .0171821305842
 0
 0
 0
 0
 0
 0
 0
 0
 0
 0
 0
 0
 0
 0
 0
 0
 0
 0
 0
 0
 0
 0
 0
 0
 0
 0
 0
 0
 0
 0
 0
 0
 0
 0
 0
 0
 0
 0
 0
 0
 0
 0
 0
 0
 0
 0
 0
 .213058419244
 0
 0
 0
 0
 0
 0
 0
 0
 0
 0
 0
 0
 0
 0
 0
 0
 .213058419244
 .213058419244
 .213058419244
 .213058419244
 .213058419244
 0
 0
 0
 0
 0
 0
 0
 0
 0
 0
 0
 0
 0
 0
 0
 0
 .020618556701
 .020618556701
 .020618556701
 .020618556701
 .020618556701
 .020618556701
 .0309278350515
 .020618556701
 .020618556701
 .020618556701
 .020618556701
 .020618556701
 .0103092783505
 .0103092783505
 .0103092783505
 .0103092783505
 .0103092783505
 0
 0
 0
 0
 0
 0
 0
 0
 8.72852233676914
 8.42955326460378
 1.26804123711684
 .00343642611684
 .00343642611684
 .00343642611684
 0
 0
 1.264604811
 1.264604811
 1.264604811
 0
 0
 0
 0
 0
 0
 0
 7.16151202748694
 2.0034364261147
 2.0034364261147
 .0274914089347
 1.97594501718
 5.15807560137224
 0
 0
 .134020618557
 .134020618557
 4.79381443298684
 0
 0
 0
 0
 0
 0
 .00343642611684
 0
 4.79037800687
 .175257731959
 .175257731959
 0
 0
 0
 .0549828178694
 .0549828178694
 0
 0
 0
 0
 0
 0
 0
 0
 0
 0
 0
 0
 0
 0
 0
 0
 0
 0
 .29553264604852
 .29553264604852
 .285223367698
 .285223367698
 0
 0
 .285223367698
 0
 0
 .01030927835052
 .00687285223368
 .00687285223368
 .00343642611684
 .00343642611684
 0
 0
 0
 0
 .00343642611684
 .00343642611684
 .00343642611684
 .00343642611684
 .00343642611684
 0
 0
 .0412371134021
 0
 0
 0
 0
 0
 .0412371134021
 .0412371134021
 .0412371134021
 .0412371134021
 .0412371134021
 0
 0
 0
 0
 0
 0
 0
 0
 0
 0
 0
 0
 0
 0
 0
 0
 0
 0
 0
 0
 0
 0
 0
 0
 0
 0
 33.9621993126835
 1.20618556701
 1.20618556701
 1.20618556701
 1.20618556701
 1.20618556701
 .127147766323
 .127147766323
 .127147766323
 0
 0
 0
 0
 .127147766323
 .127147766323
 32.6288659793505
 32.6288659793505
 .0103092783505
 .0103092783505
 .0103092783505
 32.618556701
 32.618556701
 32.618556701
 .0274914089347
 .0274914089347
 .0274914089347
 .0274914089347
 .0274914089347
 .0274914089347
 .178694158076
 .178694158076
 .178694158076
 .178694158076
 .178694158076
 .178694158076
 .178694158076
