# Supplementary material for: Assessment of biomass potentials of microalgal communities in open pond raceways using mass cultivation
Source: PeerJ. 2020 Jul 16;8:e9418. doi: 10.7717/peerj.9418 (PMC7369025; doi:10.7717/peerj.9418)
Supplement: Data S3 [file peerj-08-9418-s020.zip › Krona/OPR#1/OPR#1_JUL.html]

Javascript must be enabled to view this page.

magnitude
 100.000000000083
 99.9657924744272
 42.0524515393834
 19.6921322691
 19.6921322691
 19.6921322691
 19.6921322691
 19.6921322691
 21.7065754466417
 21.5735461802028
 18.7989357659111
 16.415811478568
 0
 0
 .114025085519
 0
 0
 0
 13.3751425314
 2.81261877613
 0
 .114025085519
 0
 0
 0
 .0532117065754
 .0532117065754
 .0266058532877
 .0266058532877
 0
 0
 2.30330672748
 0
 2.30330672748
 0
 0
 0
 0
 0
 0
 0
 0
 0
 2.59977194983
 2.59977194983
 2.59977194983
 0
 0
 0
 .0266058532877
 .0266058532877
 .0266058532877
 .148232611174
 .148232611174
 0
 0
 .148232611174
 0
 0
 0
 0
 0
 0
 0
 0
 0
 0
 0
 0
 0
 0
 0
 0
 0
 0
 0
 0
 .121626757887
 .121626757887
 .121626757887
 0
 .121626757887
 0
 0
 0
 .0114025085519
 .0114025085519
 .0114025085519
 .0114025085519
 0
 0
 0
 0
 0
 0
 0
 0
 0
 0
 0
 0
 0
 0
 0
 0
 0
 0
 0
 0
 0
 0
 0
 0
 0
 .00380083618396
 0
 0
 0
 0
 0
 0
 0
 0
 .00380083618396
 .00380083618396
 .00380083618396
 .00380083618396
 .00760167236792
 .00760167236792
 .00760167236792
 .00760167236792
 .00760167236792
 0
 0
 0
 0
 0
 0
 0
 0
 0
 .114025085519
 .114025085519
 0
 0
 0
 .114025085519
 .114025085519
 .114025085519
 0
 0
 0
 0
 0
 0
 0
 0
 0
 0
 0
 0
 0
 0
 0
 0
 0
 0
 0
 0
 0
 .5283162295708
 .0228050171038
 .0228050171038
 .0228050171038
 .0228050171038
 .505511212467
 .505511212467
 .505511212467
 .505511212467
 0
 0
 0
 0
 0
 0
 0
 0
 0
 0
 0
 0
 0
 0
 0
 0
 0
 0
 0
 0
 0
 .205245153934
 0
 0
 0
 0
 0
 0
 0
 0
 0
 0
 0
 0
 0
 0
 0
 0
 .205245153934
 .205245153934
 .205245153934
 .205245153934
 .205245153934
 0
 0
 0
 0
 0
 0
 0
 0
 0
 0
 0
 0
 0
 0
 0
 0
 .0152033447358
 .0152033447358
 .0152033447358
 .0152033447358
 .0152033447358
 .0152033447358
 .870391486127
 .870391486127
 .870391486127
 .870391486127
 .870391486127
 .870391486127
 0
 0
 0
 0
 0
 0
 0
 0
 0
 0
 0
 0
 0
 55.503610794413
 49.2930444698182
 .0570125427594
 .0304066894717
 .0304066894717
 .0304066894717
 0
 0
 .0266058532877
 .0266058532877
 .0266058532877
 0
 0
 0
 0
 0
 0
 0
 49.2360319270588
 5.5682250095017
 5.5682250095017
 .0304066894717
 5.53781832003
 43.6678069175571
 0
 0
 2.17407829723
 2.17407829723
 39.8821740783273
 0
 0
 0
 0
 0
 0
 .0646142151273
 0
 39.8175598632
 .96921322691
 .96921322691
 .0190041809198
 .0190041809198
 0
 .62333713417
 .62333713417
 0
 0
 0
 0
 0
 0
 0
 0
 0
 0
 0
 0
 0
 0
 0
 0
 0
 0
 6.17635879893922
 6.17635879893922
 6.10414291144396
 6.10414291144396
 0
 .00380083618396
 6.10034207526
 0
 0
 .07221588749526
 .0684150513113
 .0684150513113
 .00380083618396
 .00380083618396
 0
 0
 0
 0
 .0342075256556
 .0342075256556
 .0342075256556
 .0342075256556
 .0342075256556
 0
 0
 .687951349297
 0
 0
 0
 0
 0
 .687951349297
 .687951349297
 .687951349297
 .687951349297
 .687951349297
 0
 0
 0
 0
 0
 0
 0
 0
 0
 0
 .00380083618396
 0
 0
 0
 0
 0
 0
 0
 0
 0
 0
 .00380083618396
 .00380083618396
 .00380083618396
 .00380083618396
 .00380083618396
 .627137970353
 0
 0
 0
 0
 0
 .627137970353
 .627137970353
 .627137970353
 0
 0
 0
 0
 .627137970353
 .627137970353
 0
 0
 0
 0
 0
 0
 0
 0
 0
 0
 0
 0
 0
 0
 .0342075256556
 .0342075256556
 .0342075256556
 .0342075256556
 .0342075256556
 .0342075256556
 .0342075256556
