# Supplementary material for: Assessment of biomass potentials of microalgal communities in open pond raceways using mass cultivation
Source: PeerJ. 2020 Jul 16;8:e9418. doi: 10.7717/peerj.9418 (PMC7369025; doi:10.7717/peerj.9418)
Supplement: Data S3 [file peerj-08-9418-s020.zip › Krona/OPR#1/OPR#1_JUN.html]

Javascript must be enabled to view this page.

magnitude
 100.000000000052
 99.9791401573353
 19.4497173491394
 1.72302300841
 1.72302300841
 1.72302300841
 1.72302300841
 1.72302300841
 17.7246083564577
 17.6933185923826
 15.8075888107808
 15.8075888107808
 0
 0
 .166878741734
 0
 0
 .0208598427168
 0
 7.92674023238
 0
 7.69310999395
 0
 0
 0
 0
 0
 0
 0
 0
 0
 0
 0
 0
 0
 0
 0
 0
 0
 0
 0
 0
 0
 1.8711278917
 1.8711278917
 1.8711278917
 0
 0
 0
 .0146018899018
 .0146018899018
 .0146018899018
 0
 0
 0
 0
 0
 0
 0
 0
 0
 0
 0
 0
 0
 0
 0
 0
 0
 0
 0
 0
 0
 0
 0
 0
 0
 .0250318112601
 .0250318112601
 .0250318112601
 0
 .0250318112601
 0
 0
 0
 0
 0
 0
 0
 .00625795281504
 .00625795281504
 .00625795281504
 .00625795281504
 0
 0
 0
 0
 0
 0
 0
 0
 0
 0
 0
 0
 0
 0
 0
 0
 0
 0
 0
 0
 0
 0
 0
 0
 0
 0
 0
 0
 0
 0
 0
 0
 0
 0
 0
 0
 0
 0
 0
 .00208598427168
 0
 0
 0
 0
 .00208598427168
 .00208598427168
 .00208598427168
 .00208598427168
 0
 0
 0
 0
 0
 0
 0
 0
 0
 0
 0
 0
 0
 0
 0
 0
 0
 0
 0
 0
 0
 0
 0
 0
 0
 0
 0
 0
 0
 0
 0
 0
 0
 0
 0
 0
 0
 0
 0
 0
 0
 0
 0
 0
 0
 0
 0
 0
 0
 0
 0
 0
 0
 0
 0
 0
 0
 0
 0
 .00625795281504
 0
 0
 0
 0
 0
 0
 0
 0
 0
 0
 0
 0
 0
 0
 0
 0
 .00625795281504
 .00625795281504
 .00625795281504
 .00625795281504
 .00625795281504
 0
 0
 0
 0
 0
 0
 0
 0
 0
 0
 0
 0
 0
 0
 0
 0
 .0104299213584
 .0104299213584
 .0104299213584
 .0104299213584
 .0104299213584
 .0104299213584
 .0312897640752
 .0312897640752
 .0312897640752
 .0312897640752
 .0312897640752
 .0312897640752
 0
 0
 0
 0
 0
 0
 0
 0
 0
 0
 0
 0
 0
 80.3312543023868
 78.0617034148051
 0
 0
 0
 0
 0
 0
 0
 0
 0
 0
 0
 0
 0
 0
 0
 0
 78.0617034148051
 .0250318112601
 .0250318112601
 0
 .0250318112601
 78.0053818394698
 0
 0
 .31081165648
 .31081165648
 77.427564196215
 0
 0
 0
 0
 0
 0
 .00625795281504
 0
 77.4213062434
 .0917833079539
 .0917833079539
 .0333757483469
 0
 .0333757483469
 .141846930474
 .141846930474
 0
 0
 0
 0
 0
 0
 0
 0
 .0312897640752
 0
 0
 .0312897640752
 .0312897640752
 0
 0
 0
 0
 0
 2.26746490331
 2.26746490331
 2.26746490331
 2.26746490331
 0
 0
 2.26746490331
 0
 0
 0
 0
 0
 0
 0
 0
 0
 0
 0
 .00208598427168
 .00208598427168
 .00208598427168
 .00208598427168
 0
 0
 .00208598427168
 .00625795281504
 0
 0
 0
 0
 0
 .00625795281504
 .00625795281504
 .00625795281504
 .00625795281504
 .00625795281504
 0
 0
 0
 0
 0
 0
 0
 0
 0
 0
 .00417196854336
 0
 0
 0
 0
 0
 0
 0
 0
 0
 0
 .00417196854336
 .00417196854336
 .00417196854336
 .00417196854336
 .00417196854336
 .139760946202
 0
 0
 0
 0
 0
 .139760946202
 .139760946202
 .139760946202
 0
 0
 .139760946202
 .139760946202
 0
 0
 0
 0
 0
 0
 0
 0
 0
 0
 0
 0
 0
 0
 0
 0
 .0208598427168
 .0208598427168
 .0208598427168
 .0208598427168
 .0208598427168
 .0208598427168
 .0208598427168
