# Supplementary material for: Assessment of biomass potentials of microalgal communities in open pond raceways using mass cultivation
Source: PeerJ. 2020 Jul 16;8:e9418. doi: 10.7717/peerj.9418 (PMC7369025; doi:10.7717/peerj.9418)
Supplement: Data S3 [file peerj-08-9418-s020.zip › Krona/OPR#1/OPR#1_MAY.html]

Javascript must be enabled to view this page.

magnitude
 99.9999999999595
 99.9820998502492
 5.33424461368285
 .00325457267461
 .00325457267461
 .00325457267461
 .00325457267461
 .00325457267461
 5.3179717503098
 5.2626440148415
 2.4474386513085
 2.4474386513085
 0
 0
 2.28959187659
 .0016272863373
 0
 0
 0
 .0764824578533
 0
 .0797370305279
 0
 0
 0
 0
 0
 0
 0
 0
 0
 0
 0
 0
 0
 0
 0
 0
 0
 0
 0
 0
 0
 .408448870663
 .408448870663
 .408448870663
 0
 0
 0
 2.40675649287
 2.40675649287
 2.40675649287
 0
 0
 0
 0
 0
 0
 0
 0
 0
 0
 0
 0
 0
 0
 0
 0
 0
 0
 0
 0
 0
 0
 0
 0
 0
 .0423094447699
 .0423094447699
 .0423094447699
 0
 .0423094447699
 0
 0
 0
 0
 0
 0
 0
 .0130182906984
 .0130182906984
 .0130182906984
 .0130182906984
 0
 0
 0
 0
 0
 0
 0
 0
 0
 0
 0
 0
 0
 0
 0
 0
 0
 0
 0
 0
 0
 0
 0
 0
 0
 0
 0
 0
 0
 0
 0
 0
 0
 0
 0
 0
 0
 0
 0
 .01301829069844
 .00650914534922
 .00650914534922
 .00650914534922
 .00650914534922
 .00650914534922
 .00650914534922
 .00650914534922
 .00650914534922
 0
 0
 0
 0
 0
 0
 0
 0
 0
 0
 0
 0
 0
 0
 0
 0
 0
 0
 0
 0
 0
 0
 0
 0
 0
 0
 0
 0
 0
 0
 0
 0
 0
 0
 0
 0
 0
 0
 0
 0
 0
 0
 0
 0
 0
 0
 0
 0
 0
 0
 0
 0
 0
 0
 0
 0
 0
 0
 0
 0
 0
 0
 0
 0
 0
 0
 0
 0
 0
 0
 0
 0
 0
 0
 0
 0
 0
 0
 0
 0
 0
 0
 0
 0
 0
 0
 0
 0
 0
 0
 0
 0
 0
 0
 0
 0
 0
 .00325457267461
 .00325457267461
 .00325457267461
 .00325457267461
 .00325457267461
 .00325457267461
 .00650914534922
 .00325457267461
 .00325457267461
 .00325457267461
 .00325457267461
 .00325457267461
 0
 0
 0
 0
 0
 0
 0
 0
 .00325457267461
 .00325457267461
 .00325457267461
 .00325457267461
 .00325457267461
 94.6364642322052
 94.0490138644383
 0
 0
 0
 0
 0
 0
 0
 0
 0
 0
 0
 0
 0
 0
 0
 0
 94.0490138644383
 .0113910043611
 .0113910043611
 0
 .0113910043611
 93.9888042699581
 0
 0
 .177374210766
 .177374210766
 91.7122306840746
 0
 0
 0
 0
 0
 0
 .00325457267461
 0
 91.7089761114
 .0406821584326
 .0406821584326
 .0634641671549
 0
 .0634641671549
 1.99505304953
 1.99505304953
 0
 0
 0
 0
 0
 0
 0
 0
 .0488185901191
 0
 0
 .0488185901191
 .0488185901191
 0
 0
 0
 0
 0
 .58745036776691
 .58745036776691
 .582568508755
 .582568508755
 0
 0
 .582568508755
 0
 0
 .00488185901191
 .0016272863373
 .0016272863373
 .00325457267461
 .00325457267461
 0
 0
 0
 0
 0
 0
 0
 0
 0
 0
 0
 0
 0
 0
 0
 0
 0
 0
 0
 0
 0
 0
 0
 0
 0
 0
 0
 0
 0
 0
 0
 0
 0
 0
 0
 0
 0
 0
 0
 0
 0
 0
 0
 0
 0
 0
 0
 0
 .0016272863373
 0
 0
 0
 0
 0
 .0016272863373
 .0016272863373
 .0016272863373
 0
 0
 .0016272863373
 .0016272863373
 0
 0
 0
 0
 0
 0
 0
 0
 0
 0
 0
 0
 0
 0
 0
 0
 .0179001497103
 .0179001497103
 .0179001497103
 .0179001497103
 .0179001497103
 .0179001497103
 .0179001497103
