# Supplementary material for: Assessment of biomass potentials of microalgal communities in open pond raceways using mass cultivation
Source: PeerJ. 2020 Jul 16;8:e9418. doi: 10.7717/peerj.9418 (PMC7369025; doi:10.7717/peerj.9418)
Supplement: Data S3 [file peerj-08-9418-s020.zip › Krona/OPR#1/OPR#1_NOV.html]

Javascript must be enabled to view this page.

magnitude
 99.9999999998979
 98.7180527382379
 33.9797160242745
 .00811359026369
 .00811359026369
 .00811359026369
 .00811359026369
 .00811359026369
 33.9148073021649
 33.9066937119012
 18.4989858011822
 18.4989858011822
 0
 0
 .0243407707911
 0
 0
 0
 0
 15.5456389452
 0
 1.50912778905
 .0243407707911
 0
 1.39553752535
 0
 0
 0
 0
 0
 0
 0
 0
 0
 0
 0
 0
 0
 0
 0
 0
 0
 0
 .129817444219
 .129817444219
 .129817444219
 0
 0
 0
 15.2778904665
 15.2778904665
 15.2778904665
 0
 0
 0
 0
 0
 .00811359026369
 .00811359026369
 .00811359026369
 .00811359026369
 0
 0
 0
 0
 0
 0
 0
 0
 0
 0
 0
 0
 0
 0
 0
 0
 0
 0
 0
 0
 0
 0
 0
 0
 0
 0
 0
 0
 0
 0
 0
 0
 0
 0
 0
 0
 0
 0
 0
 0
 0
 0
 0
 0
 0
 0
 0
 0
 0
 0
 0
 0
 0
 .05679513184589
 .00811359026369
 .00811359026369
 .00811359026369
 .00811359026369
 0
 0
 0
 0
 .0486815415822
 .0486815415822
 .0486815415822
 .0486815415822
 0
 0
 0
 0
 0
 0
 0
 0
 0
 0
 0
 0
 0
 0
 0
 0
 0
 0
 0
 0
 0
 0
 0
 0
 0
 0
 0
 0
 0
 0
 0
 0
 0
 0
 0
 0
 0
 0
 0
 0
 0
 0
 0
 0
 0
 0
 0
 0
 0
 0
 0
 0
 .194726166329
 0
 0
 0
 0
 0
 0
 0
 .194726166329
 .194726166329
 .194726166329
 .194726166329
 .194726166329
 0
 0
 0
 0
 0
 0
 0
 0
 0
 0
 0
 0
 0
 0
 0
 0
 0
 0
 0
 0
 0
 0
 0
 0
 0
 0
 0
 0
 0
 0
 .11359026369169
 .11359026369169
 .105476673428
 .105476673428
 .105476673428
 .105476673428
 .00811359026369
 .00811359026369
 .00811359026369
 .00811359026369
 0
 0
 0
 0
 0
 0
 0
 0
 0
 0
 0
 0
 0
 0
 0
 0
 0
 0
 0
 0
 0
 0
 0
 0
 0
 0
 0
 0
 0
 0
 0
 64.2758620689324
 62.2474645030062
 3.03448275862
 0
 0
 0
 0
 0
 3.03448275862
 3.03448275862
 3.03448275862
 0
 0
 0
 0
 0
 0
 0
 59.1156186612219
 .146044624746
 .146044624746
 .146044624746
 0
 58.9695740364759
 .0324543610548
 .0324543610548
 1.4523326572
 1.4523326572
 57.2251521297829
 .0405679513185
 0
 0
 .00811359026369
 .0486815415822
 0
 .0405679513185
 0
 57.0872210953
 0
 0
 .2352941176471
 .210953346856
 .0243407707911
 0
 0
 .0243407707911
 0
 .0243407707911
 0
 0
 0
 0
 0
 0
 0
 0
 0
 0
 .0973630831643
 .0973630831643
 .0973630831643
 .0973630831643
 0
 2.0283975659262
 1.979716024344
 1.979716024344
 .535496957404
 .535496957404
 0
 0
 1.44421906694
 1.44421906694
 0
 0
 0
 0
 0
 .0486815415822
 .0486815415822
 .0486815415822
 .0486815415822
 0
 0
 0
 0
 0
 0
 0
 0
 0
 0
 0
 0
 0
 0
 0
 0
 0
 0
 0
 0
 0
 0
 0
 0
 0
 0
 0
 0
 0
 0
 0
 0
 0
 0
 0
 0
 0
 0
 0
 0
 0
 0
 0
 0
 .1541582150104
 0
 0
 0
 0
 0
 .0162271805274
 .0162271805274
 .0162271805274
 0
 0
 .0162271805274
 .0162271805274
 0
 0
 .137931034483
 .137931034483
 0
 0
 0
 .137931034483
 .137931034483
 .137931034483
 0
 0
 0
 0
 0
 0
 1.28194726166
 1.28194726166
 1.28194726166
 1.28194726166
 1.28194726166
 1.28194726166
 1.28194726166
