# Supplementary material for: Assessment of biomass potentials of microalgal communities in open pond raceways using mass cultivation
Source: PeerJ. 2020 Jul 16;8:e9418. doi: 10.7717/peerj.9418 (PMC7369025; doi:10.7717/peerj.9418)
Supplement: Data S3 [file peerj-08-9418-s020.zip › Krona/OPR#1/OPR#1_OCT.html]

Javascript must be enabled to view this page.

magnitude
 100.000000000029
 99.6716782087912
 17.1749973088327
 .0932936237396
 .0932936237396
 .0932936237396
 .0932936237396
 .0932936237396
 17.0494097384139
 17.036850981372
 16.8951164376138
 16.8915282213161
 0
 0
 .0161469733396
 0
 .00538232444652
 0
 0
 7.75234131113
 0
 7.85998780006
 0
 0
 1.25766981234
 0
 0
 0
 0
 .00358821629768
 .00358821629768
 0
 0
 0
 0
 0
 0
 0
 0
 0
 0
 0
 0
 .132764003014
 .132764003014
 .132764003014
 0
 0
 0
 .0089705407442
 .0089705407442
 .0089705407442
 0
 0
 0
 0
 0
 .00358821629768
 .00358821629768
 .00358821629768
 .00358821629768
 0
 0
 0
 0
 0
 0
 0
 0
 0
 0
 0
 0
 .00358821629768
 .00358821629768
 .00358821629768
 .00358821629768
 0
 0
 0
 0
 0
 0
 0
 0
 .00538232444652
 .00538232444652
 .00538232444652
 .00538232444652
 0
 0
 0
 0
 0
 0
 0
 0
 0
 0
 0
 0
 0
 0
 0
 0
 0
 0
 0
 0
 0
 0
 0
 0
 0
 .0287057303815
 .0161469733396
 .0161469733396
 .0161469733396
 .0161469733396
 0
 0
 0
 0
 .0125587570419
 .0125587570419
 .0125587570419
 .0125587570419
 0
 0
 0
 0
 0
 0
 0
 0
 0
 0
 0
 0
 0
 0
 .00179410814884
 .00179410814884
 0
 0
 0
 .00179410814884
 .00179410814884
 .00179410814884
 0
 0
 0
 0
 0
 0
 0
 0
 0
 0
 0
 0
 0
 0
 .00179410814884
 .00179410814884
 .00179410814884
 0
 0
 .00179410814884
 .00179410814884
 0
 0
 0
 0
 0
 0
 0
 0
 0
 .0143528651907
 0
 0
 0
 0
 0
 0
 0
 .0143528651907
 .0143528651907
 .0143528651907
 .0143528651907
 .0143528651907
 0
 0
 0
 0
 0
 0
 0
 0
 .00717643259536
 .00717643259536
 0
 0
 0
 0
 .00717643259536
 .00717643259536
 .00717643259536
 0
 .00717643259536
 0
 0
 0
 0
 0
 0
 0
 0
 0
 0
 0
 0
 0
 0
 0
 0
 0
 0
 0
 0
 0
 .00179410814884
 .00179410814884
 .00179410814884
 .00179410814884
 .00179410814884
 .00179410814884
 .00179410814884
 .00179410814884
 .00179410814884
 .00179410814884
 .00179410814884
 .00179410814884
 .0771466504001
 .0466468118698
 .0466468118698
 .0466468118698
 .0466468118698
 .0466468118698
 0
 0
 0
 0
 0
 0
 0
 0
 .0304998385303
 .0304998385303
 .0304998385303
 .0304998385303
 .0304998385303
 80.1697226309154
 77.1735620223505
 .251175140837
 0
 0
 0
 0
 0
 .251175140837
 .251175140837
 .251175140837
 0
 0
 0
 0
 0
 0
 0
 76.4056837346475
 .0663820015071
 .0663820015071
 .0574114607629
 .0089705407442
 76.3393017331404
 .0233234059349
 .0233234059349
 .321145358642
 .321145358642
 75.7346872869815
 .0197351896372
 0
 0
 0
 .0520291363163
 .0125587570419
 .0215292977861
 0
 75.6288349062
 0
 0
 .215292977861
 .100470056335
 .114822921526
 0
 0
 .044852703721
 0
 .044852703721
 0
 0
 0
 0
 0
 0
 0
 0
 0
 0
 .516703146866
 .516703146866
 .516703146866
 .516703146866
 0
 2.9925723922672
 2.9818077433742
 2.9818077433742
 2.97283720263
 2.97283720263
 0
 0
 .0089705407442
 .0089705407442
 0
 0
 0
 0
 0
 .010764648893
 .010764648893
 .010764648893
 .010764648893
 .00358821629768
 .00358821629768
 .00358821629768
 .00358821629768
 0
 0
 .00358821629768
 .0089705407442
 0
 0
 0
 0
 0
 .0089705407442
 .0089705407442
 .0089705407442
 .0089705407442
 .0089705407442
 .00538232444652
 .00538232444652
 .00538232444652
 .00538232444652
 .00538232444652
 .00538232444652
 0
 0
 0
 0
 0
 0
 0
 0
 0
 0
 0
 0
 0
 0
 0
 0
 0
 0
 0
 0
 2.127812264522
 0
 0
 0
 0
 0
 .954465535182
 .954465535182
 .954465535182
 0
 0
 .954465535182
 .954465535182
 0
 0
 1.17334672934
 1.17334672934
 0
 0
 0
 1.17334672934
 1.17334672934
 1.17334672934
 .0825289748466
 .0825289748466
 .0825289748466
 .0825289748466
 .0825289748466
 .0825289748466
 .328321791238
 .328321791238
 .328321791238
 .328321791238
 .328321791238
 .328321791238
 .328321791238
