# Supplementary material for: Assessment of biomass potentials of microalgal communities in open pond raceways using mass cultivation
Source: PeerJ. 2020 Jul 16;8:e9418. doi: 10.7717/peerj.9418 (PMC7369025; doi:10.7717/peerj.9418)
Supplement: Data S3 [file peerj-08-9418-s020.zip › Krona/OPR#1/OPR#1_SEP.html]

Javascript must be enabled to view this page.

magnitude
 100.000000000031
 98.0914880366712
 13.6107037386751
 2.77437370673
 2.77437370673
 2.77437370673
 2.77437370673
 2.77437370673
 10.7358820338738
 10.6414609156867
 10.6344295558217
 10.6213713160724
 0
 0
 2.56544187074
 0
 .0572553589007
 0
 .197882556201
 6.12732788236
 .00301343994214
 1.65839644816
 .00301343994214
 0
 .00904031982643
 0
 0
 0
 0
 0
 0
 .0130582397493
 0
 .0130582397493
 0
 0
 0
 0
 0
 0
 .00401791992286
 .00401791992286
 .00401791992286
 0
 0
 0
 0
 0
 0
 .00301343994214
 .00301343994214
 .00301343994214
 0
 0
 0
 0
 0
 .00301343994214
 .00301343994214
 .00301343994214
 .00301343994214
 0
 0
 0
 0
 .00401791992286
 .00401791992286
 .00401791992286
 .00401791992286
 0
 0
 0
 0
 .049219519055
 .049219519055
 .049219519055
 .049219519055
 .0381702392671
 .0381702392671
 .0381702392671
 .0381702392671
 0
 0
 0
 0
 0
 0
 0
 0
 0
 0
 0
 0
 0
 0
 0
 0
 0
 0
 0
 0
 0
 0
 0
 0
 0
 0
 0
 0
 .00602687988428
 .00602687988428
 .00602687988428
 .00602687988428
 .00602687988428
 .0713180786306
 .0462060791128
 .0462060791128
 .0462060791128
 .0462060791128
 0
 0
 0
 0
 .0251119995178
 .0251119995178
 .0251119995178
 .0251119995178
 0
 0
 0
 0
 0
 0
 0
 0
 0
 0
 0
 0
 0
 0
 .01607167969139
 .01406271972996
 .00401791992286
 .00401791992286
 .00401791992286
 .0100447998071
 .0100447998071
 .0100447998071
 .00200895996143
 .00200895996143
 .00200895996143
 .00200895996143
 0
 0
 0
 0
 0
 0
 0
 0
 0
 0
 .007031359865
 .007031359865
 .007031359865
 .007031359865
 .007031359865
 0
 0
 0
 0
 0
 0
 0
 0
 0
 0
 0
 .00200895996143
 0
 0
 0
 0
 0
 0
 0
 .00200895996143
 0
 0
 0
 0
 .00200895996143
 .00200895996143
 .00200895996143
 .00200895996143
 0
 0
 0
 0
 .31339775398285
 .31339775398285
 .29531711433
 .29531711433
 .29531711433
 .29531711433
 .01808063965285
 .01506719971071
 .01506719971071
 .00602687988428
 .00904031982643
 .00301343994214
 .00301343994214
 .00301343994214
 0
 0
 0
 0
 0
 0
 0
 0
 .2812543946
 .2812543946
 .2812543946
 .2812543946
 .2812543946
 .2812543946
 0
 0
 0
 0
 .01406271973
 .01406271973
 .01406271973
 .01406271973
 .01406271973
 .01406271973
 .00200895996143
 .00200895996143
 .00200895996143
 .00200895996143
 .00200895996143
 .00200895996143
 .242079675352
 .150671997107
 .150671997107
 .150671997107
 .150671997107
 .150671997107
 0
 0
 0
 0
 0
 0
 0
 0
 .091407678245
 .091407678245
 .091407678245
 .091407678245
 .091407678245
 79.2454346385156
 78.3876087349864
 .00502239990356
 .00100447998071
 0
 0
 .00100447998071
 .00100447998071
 .00100447998071
 .00100447998071
 .00100447998071
 0
 0
 0
 .00301343994214
 .00301343994214
 0
 .00301343994214
 78.0571348213314
 .01607167969141
 .01607167969141
 .00100447998071
 .0150671997107
 77.9938525825465
 .00502239990357
 .00502239990357
 .696104626635
 .696104626635
 77.1581252385922
 .0452015991321
 .007031359865
 0
 0
 .116519677763
 0
 .0452015991321
 0
 76.9441710027
 0
 0
 .133595837435
 .133595837435
 0
 0
 0
 .00100447998071
 0
 .00100447998071
 .0441971191514
 0
 0
 .0441971191514
 .0441971191514
 .00301343994214
 .00301343994214
 .00301343994214
 0
 0
 .32545151375143
 .32545151375143
 .32545151375143
 .32344255379
 .00200895996143
 .81865118428143
 .81664222432
 .81664222432
 .81664222432
 .81664222432
 0
 0
 0
 0
 0
 0
 0
 0
 0
 .00200895996143
 .00200895996143
 .00200895996143
 .00200895996143
 .0391747192478
 .0391747192478
 .0391747192478
 .0391747192478
 0
 0
 .0391747192478
 .0602687988428
 0
 0
 0
 0
 0
 .0602687988428
 .0602687988428
 .0602687988428
 .0602687988428
 .0602687988428
 0
 0
 0
 0
 0
 0
 0
 0
 0
 0
 0
 0
 0
 0
 0
 0
 0
 0
 0
 0
 0
 0
 0
 0
 0
 0
 4.32026839705
 0
 0
 0
 0
 0
 0
 0
 0
 0
 0
 0
 0
 0
 0
 4.32026839705
 4.32026839705
 0
 0
 0
 4.32026839705
 4.32026839705
 4.32026839705
 0
 0
 0
 0
 0
 0
 1.90851196336
 1.90851196336
 1.90851196336
 1.90851196336
 1.90851196336
 1.90851196336
 1.90851196336
