# Supplementary material for: Assessment of biomass potentials of microalgal communities in open pond raceways using mass cultivation
Source: PeerJ. 2020 Jul 16;8:e9418. doi: 10.7717/peerj.9418 (PMC7369025; doi:10.7717/peerj.9418)
Supplement: Data S3 [file peerj-08-9418-s020.zip › Krona/OPR#1.html]

Javascript must be enabled to view this page.

magnitude
 99.9999999999948
 99.5237832007436
 26.5226298354595
 4.46033442886366
 4.46033442886366
 4.46033442886366
 4.46033442886366
 4.46033442886366
 5.9079991607975E-03
 5.9079991607975E-03
 5.9079991607975E-03
 5.9079991607975E-03
 5.9079991607975E-03
 3.33880599446156
 8.80833033951125E-03
 8.80833033951125E-03
 8.80833033951125E-03
 8.80833033951125E-03
 1.2886597938125E-03
 1.2886597938125E-03
 1.2886597938125E-03
 1.2886597938125E-03
 3.32870900432823
 3.32870900432823
 3.32870900432823
 3.32870900432823
 .00095020904599
 .00095020904599
 .00095020904599
 .00095020904599
 .00095020904599
 .001888034371265
 8.136431686525E-04
 8.136431686525E-04
 8.136431686525E-04
 8.136431686525E-04
 1.0743912026125E-03
 1.0743912026125E-03
 1.0743912026125E-03
 1.0743912026125E-03
 3.19502766956788E-02
 .029551390377475
 1.38183911931075E-02
 1.38183911931075E-02
 1.38183911931075E-02
 1.57329991843675E-02
 1.57329991843675E-02
 1.57329991843675E-02
 2.39888631820375E-03
 2.39888631820375E-03
 2.39888631820375E-03
 2.39888631820375E-03
 .00110318350173
 .00110318350173
 .00110318350173
 .000878919983125
 .000878919983125
 .000224263518605
 .000224263518605
 .06603952869635
 .002850627137975
 .002850627137975
 .002850627137975
 .002850627137975
 .063188901558375
 .063188901558375
 .063188901558375
 .063188901558375
 18.6156501806624
 1.83940581293875E-03
 1.83940581293875E-03
 1.83940581293875E-03
 1.83940581293875E-03
 .040378006872875
 .040378006872875
 .040378006872875
 .040378006872875
 5.022399903575E-04
 5.022399903575E-04
 5.022399903575E-04
 5.022399903575E-04
 .002147766323025
 .002147766323025
 .002147766323025
 .002147766323025
 .006600966919085
 .006600966919085
 .006600966919085
 .006600966919085
 .0461482151
 .0461482151
 .0461482151
 4.7712799083875E-03
 4.13769351916125E-02
 .00510497697704
 .00510497697704
 .00510497697704
 .00510497697704
 6.0112697518425E-03
 6.0112697518425E-03
 6.0112697518425E-03
 6.0112697518425E-03
 18.5069173329153
 5.022399903575E-04
 5.022399903575E-04
 5.022399903575E-04
 .732687716194125
 .732687716194125
 .732687716194125
 2.21821212794554
 2.21821212794554
 2.21821212794554
 .018958629661355
 .018958629661355
 .000429553264605
 .01852907639675
 15.5365566191239
 .028129126552175
 .028129126552175
 3.7552849255675E-03
 3.7552849255675E-03
 .00044852703721
 .00044852703721
 .29856623946036
 8.1615120274875E-03
 .290404727432872
 15.2056574411486
 5.5841924398625E-03
 3.64414827938134
 2.034107921625E-04
 7.8297104184025E-03
 .0026074803396
 1.7125216067405
 7.10695267616469
 3.766799927675E-04
 2.38923317134804
 .003419276341655
 .332780957189554
 2.63859989351413E-02
 2.63859989351413E-02
 2.61348789399625E-02
 2.61348789399625E-02
 2.61348789399625E-02
 2.61348789399625E-02
 2.5111999517875E-04
 2.5111999517875E-04
 2.5111999517875E-04
 2.5111999517875E-04
 9.44516874578313E-02
 4.13814967087013E-02
 .03691463929125
 .03691463929125
 .03691463929125
 .03691463929125
 4.46685741745125E-03
 2.78045403825875E-03
 2.78045403825875E-03
 .000753359985535
 2.02709405272375E-03
 3.766799927675E-04
 3.766799927675E-04
 3.766799927675E-04
 .001309723386425
 .001309723386425
 .001309723386425
 .05307019074913
 .05307019074913
 .05307019074913
 .05307019074913
 .05307019074913
 4.93555822864613E-02
 4.93555822864613E-02
 .0483413835035
 .0483413835035
 .0483413835035
 .0483413835035
 1.01419878296125E-03
 1.01419878296125E-03
 1.01419878296125E-03
 1.01419878296125E-03
 .001982103484855
 .001982103484855
 .001982103484855
 .001982103484855
 .001982103484855
 .001982103484855
 .00666368294751
 .00666368294751
 .00666368294751
 .00666368294751
 .00666368294751
 .00666368294751
 .165151409862903
 .140359148569326
 .140359148569326
 .140359148569326
 .140359148569326
 .140359148569326
 1.2886597938125E-03
 1.2886597938125E-03
 1.2886597938125E-03
 1.2886597938125E-03
 1.2886597938125E-03
 2.35036014997638E-02
 2.35036014997638E-02
 2.35036014997638E-02
 2.35036014997638E-02
 2.35036014997638E-02
 67.3745346927827
 65.3760841027739
 .5769667599046
 4.35594944615625E-03
 4.2303894485675E-03
 4.2303894485675E-03
 1.2555999758875E-04
 1.2555999758875E-04
 .572234130465676
 .572234130465676
 .572234130465676
 3.766799927675E-04
 3.766799927675E-04
 3.766799927675E-04
 64.6816776248966
 .980227681340974
 .980227681340974
 3.27948329870012E-02
 .947432848353973
 63.6822607709286
 7.60002086165875E-03
 7.60002086165875E-03
 .67689698644525
 .67689698644525
 62.2278878269076
 .013188092510975
 .000878919983125
 1.01419878296125E-03
 2.71537944576875E-02
 1.5698446302375E-03
 2.31077518713113E-02
 62.1609752246713
 .161581638236562
 .161581638236562
 9.20874105230625E-02
 .058002927693225
 3.40844828298375E-02
 .507432143642925
 .507432143642925
 8.77474431160125E-03
 8.77474431160125E-03
 .005524639893925
 .005524639893925
 .005524639893925
 .013664532733105
 3.766799927675E-04
 3.766799927675E-04
 1.32878527403375E-02
 1.32878527403375E-02
 .117439717972716
 .117439717972716
 .117439717972716
 .117188597977538
 2.5111999517875E-04
 1.98781155021344
 1.98012965640886
 1.96920377830164
 1.78755507734112
 .54062204804425
 .000475104522995
 1.24645792477388
 .181648700960525
 .181648700960525
 1.09258781072113E-02
 .009614398735285
 .009614398735285
 1.31147937192625E-03
 1.31147937192625E-03
 7.68189380457875E-03
 7.68189380457875E-03
 7.68189380457875E-03
 7.68189380457875E-03
 .010639039795305
 .010639039795305
 .010639039795305
 .010639039795305
 .00503292481816
 .005606114977145
 .100585719387642
 .100585719387642
 .100585719387642
 .100585719387642
 .100585719387642
 .100585719387642
 .000672790555815
 .000672790555815
 .000672790555815
 .000672790555815
 .000672790555815
 .000672790555815
 .000996600590915
 .000996600590915
 .000996600590915
 .000996600590915
 .000996600590915
 .000996600590915
 5.16662054901978
 .15077319587625
 .15077319587625
 .15077319587625
 .15077319587625
 .15077319587625
 .233295835615588
 .233295835615588
 .233295835615588
 .139010118531088
 .139010118531088
 .0942857170845
 .0942857170845
 4.78255151752794
 4.78255151752794
 1.2886597938125E-03
 1.2886597938125E-03
 1.2886597938125E-03
 4.78126285773413
 4.78126285773413
 4.78126285773413
 1.37525479726625E-02
 1.37525479726625E-02
 1.37525479726625E-02
 1.37525479726625E-02
 1.37525479726625E-02
 1.37525479726625E-02
 .476216799251163
 .476216799251163
 .476216799251163
 .476216799251163
 .476216799251163
 .476216799251163
 .476216799251163
