# Supplementary material for: Assessment of biomass potentials of microalgal communities in open pond raceways using mass cultivation
Source: PeerJ. 2020 Jul 16;8:e9418. doi: 10.7717/peerj.9418 (PMC7369025; doi:10.7717/peerj.9418)
Supplement: Data S3 [file peerj-08-9418-s020.zip › Krona/OPR#3/OPR#3_APR.html]

Javascript must be enabled to view this page.

magnitude
 99.9999999999554
 99.9999999999554
 1.2462586874553
 0
 0
 0
 0
 0
 1.23103978896539
 1.21412990175433
 .86747721392733
 .86747721392733
 0
 0
 .27563116154
 0
 0
 .00169098872111
 .00169098872111
 .586773086224
 0
 .00169098872111
 0
 0
 0
 0
 0
 0
 0
 0
 0
 0
 0
 0
 0
 0
 0
 0
 0
 0
 0
 0
 0
 .142043052573
 .142043052573
 .142043052573
 0
 0
 0
 .204609635254
 .204609635254
 .204609635254
 0
 0
 0
 0
 0
 0
 0
 0
 0
 0
 0
 0
 0
 0
 0
 0
 0
 0
 0
 0
 0
 0
 0
 0
 0
 .00676395488442
 .00676395488442
 .00676395488442
 0
 .00676395488442
 0
 0
 0
 0
 0
 0
 0
 .00169098872111
 .00169098872111
 .00169098872111
 .00169098872111
 .00845494360553
 0
 0
 0
 .00845494360553
 .00845494360553
 .00845494360553
 0
 0
 0
 0
 0
 0
 0
 0
 0
 0
 0
 0
 0
 0
 .00338197744221
 0
 0
 0
 0
 0
 0
 0
 0
 .00338197744221
 .00338197744221
 .00338197744221
 .00338197744221
 0
 0
 0
 0
 0
 0
 0
 0
 0
 0
 0
 0
 0
 0
 0
 0
 0
 0
 0
 0
 0
 0
 0
 0
 0
 0
 0
 0
 0
 0
 0
 0
 0
 0
 0
 0
 0
 0
 0
 0
 0
 0
 0
 .0118369210477
 0
 0
 0
 0
 .0118369210477
 .0118369210477
 .0118369210477
 .0118369210477
 0
 0
 0
 0
 0
 0
 0
 0
 0
 0
 0
 0
 0
 0
 0
 0
 0
 0
 0
 0
 0
 0
 0
 0
 0
 0
 0
 0
 0
 0
 0
 0
 0
 0
 0
 0
 0
 0
 0
 0
 0
 0
 0
 0
 0
 0
 0
 0
 0
 0
 0
 0
 0
 0
 0
 0
 0
 0
 0
 0
 0
 0
 0
 0
 0
 .00169098872111
 .00169098872111
 .00169098872111
 .00169098872111
 .00169098872111
 .00169098872111
 0
 0
 0
 0
 0
 0
 0
 0
 0
 0
 0
 0
 0
 98.7469773576156
 98.2413717300044
 .0507296616332
 0
 0
 0
 0
 0
 0
 0
 0
 .0507296616332
 .0507296616332
 .0507296616332
 0
 0
 0
 0
 98.1906420683712
 .0101459323266
 .0101459323266
 0
 .0101459323266
 98.1771141586024
 0
 0
 .958790604867
 .958790604867
 90.8145492669121
 0
 0
 .0490386729121
 0
 0
 0
 0
 0
 90.765510594
 .00169098872111
 .00169098872111
 .00338197744221
 0
 .00338197744221
 6.39870132066
 6.39870132066
 0
 0
 0
 0
 0
 0
 0
 0
 .00338197744221
 0
 0
 .00338197744221
 .00338197744221
 0
 0
 0
 0
 0
 .35003466526911
 .35003466526911
 .348343676548
 .348343676548
 0
 0
 .348343676548
 0
 0
 .00169098872111
 0
 0
 .00169098872111
 .00169098872111
 0
 0
 0
 0
 .1555709623421
 .1555709623421
 .1555709623421
 .1555709623421
 .138661075131
 .0169098872111
 0
 .00507296616332
 0
 0
 0
 0
 0
 .00507296616332
 .00507296616332
 .00507296616332
 .00507296616332
 .00507296616332
 0
 0
 0
 0
 0
 0
 0
 0
 0
 0
 0
 0
 0
 0
 0
 0
 0
 0
 0
 0
 0
 0
 0
 0
 0
 0
 0
 0
 0
 0
 0
 0
 0
 0
 0
 0
 0
 0
 0
 0
 0
 0
 0
 0
 0
 0
 0
 0
 0
 0
 0
 0
 0
 0
 0
 0
 0
 0
 0
 0
 0
 0
