# Supplementary material for: Assessment of biomass potentials of microalgal communities in open pond raceways using mass cultivation
Source: PeerJ. 2020 Jul 16;8:e9418. doi: 10.7717/peerj.9418 (PMC7369025; doi:10.7717/peerj.9418)
Supplement: Data S3 [file peerj-08-9418-s020.zip › Krona/OPR#3/OPR#3_AUG.html]

Javascript must be enabled to view this page.

magnitude
 100.000000000026
 99.9424394175133
 48.2587923790303
 .52955735912
 .52955735912
 .52955735912
 .52955735912
 .52955735912
 45.124618661197
 44.9001323893958
 24.2243711506675
 24.2186150924162
 .00575605825131
 0
 .0978529902723
 0
 .00863408737696
 0
 0
 7.20082887239
 .00287802912565
 16.902665055
 0
 0
 0
 .00287802912565
 .00287802912565
 0
 0
 0
 0
 .00287802912565
 0
 .00287802912565
 .00575605825131
 .00575605825131
 .00575605825131
 0
 0
 0
 0
 0
 0
 20.6613710931
 20.6613710931
 20.6613710931
 0
 0
 0
 .00863408737696
 .00863408737696
 .00863408737696
 0
 0
 0
 0
 0
 0
 0
 0
 0
 .126633281529
 .126633281529
 .126633281529
 .126633281529
 0
 0
 0
 0
 0
 0
 0
 0
 0
 0
 0
 0
 .0115121165026
 .0115121165026
 .0115121165026
 0
 .0115121165026
 0
 0
 0
 0
 0
 0
 0
 .0863408737696
 .0863408737696
 .0863408737696
 .0863408737696
 0
 0
 0
 0
 0
 0
 0
 0
 0
 0
 0
 0
 0
 0
 0
 0
 0
 0
 0
 0
 0
 1.75271973752
 0
 0
 0
 0
 0
 0
 0
 0
 1.75271973752
 1.75271973752
 1.75271973752
 1.75271973752
 0
 0
 0
 0
 0
 0
 0
 0
 0
 0
 0
 0
 0
 0
 .85189662119331
 .846140562942
 0
 0
 0
 .846140562942
 .846140562942
 .846140562942
 .00575605825131
 .00575605825131
 .00575605825131
 .00575605825131
 0
 0
 0
 0
 0
 0
 0
 0
 0
 0
 0
 0
 0
 0
 0
 0
 0
 0
 0
 0
 0
 0
 0
 0
 0
 0
 0
 0
 0
 0
 0
 0
 0
 0
 0
 0
 0
 0
 0
 0
 0
 0
 0
 0
 0
 0
 0
 .0201462038796
 0
 0
 0
 0
 0
 0
 0
 0
 0
 0
 0
 0
 0
 0
 0
 0
 .0201462038796
 .0201462038796
 .0201462038796
 .0201462038796
 .0201462038796
 0
 0
 0
 0
 0
 0
 0
 0
 0
 0
 0
 0
 0
 0
 0
 0
 0
 0
 0
 0
 0
 0
 .103609048524
 .103609048524
 .103609048524
 .103609048524
 .103609048524
 .103609048524
 0
 0
 0
 0
 0
 0
 0
 0
 0
 0
 0
 0
 0
 51.5253554365716
 51.4735509123098
 .04317043688485
 0
 0
 0
 0
 0
 .0402924077592
 .0402924077592
 .0402924077592
 .00287802912565
 .00287802912565
 .00287802912565
 0
 0
 0
 0
 51.4303804754249
 .0460484660105
 .0460484660105
 0
 .0460484660105
 51.3756979220375
 0
 0
 .227364300927
 .227364300927
 51.1166753007283
 .0143901456283
 0
 0
 0
 0
 0
 0
 0
 51.1022851551
 .00287802912565
 .00287802912565
 0
 0
 0
 .0287802912565
 .0287802912565
 0
 0
 0
 0
 0
 0
 0
 0
 .00863408737696
 .00863408737696
 .00863408737696
 0
 0
 0
 0
 0
 0
 0
 .0460484660105
 .0460484660105
 .0460484660105
 .0460484660105
 0
 0
 .0460484660105
 0
 0
 0
 0
 0
 0
 0
 0
 0
 0
 0
 .00575605825131
 .00575605825131
 .00575605825131
 .00575605825131
 .00575605825131
 0
 0
 .0230242330052
 0
 0
 0
 0
 0
 .0230242330052
 .0230242330052
 .0230242330052
 .0230242330052
 .0230242330052
 0
 0
 0
 0
 0
 0
 0
 0
 0
 0
 0
 0
 0
 0
 0
 0
 0
 0
 0
 0
 0
 0
 0
 0
 0
 0
 .01151211650261
 .00863408737696
 .00863408737696
 .00863408737696
 .00863408737696
 .00863408737696
 .00287802912565
 .00287802912565
 .00287802912565
 0
 0
 0
 0
 .00287802912565
 .00287802912565
 0
 0
 0
 0
 0
 0
 0
 0
 0
 0
 0
 0
 0
 0
 .0575605825131
 .0575605825131
 .0575605825131
 .0575605825131
 .0575605825131
 .0575605825131
 .0575605825131
