# Supplementary material for: Assessment of biomass potentials of microalgal communities in open pond raceways using mass cultivation
Source: PeerJ. 2020 Jul 16;8:e9418. doi: 10.7717/peerj.9418 (PMC7369025; doi:10.7717/peerj.9418)
Supplement: Data S3 [file peerj-08-9418-s020.zip › Krona/OPR#3/OPR#3_JUL.html]

Javascript must be enabled to view this page.

magnitude
 100.000000000007
 99.9667553191556
 34.3977463605627
 .0909854423292
 .0909854423292
 .0909854423292
 .0909854423292
 .0909854423292
 34.2752659574272
 34.2385218364865
 2.12241041433636
 2.08216685330616
 0
 0
 .479423292273
 .00174972004479
 .00174972004479
 0
 .00174972004479
 .260708286674
 0
 1.33503639418
 0
 .00174972004479
 0
 0
 0
 0
 0
 0
 0
 .0402435610302
 0
 .0402435610302
 0
 0
 0
 0
 0
 0
 0
 0
 0
 31.8641517357
 31.8641517357
 31.8641517357
 0
 0
 0
 .244960806271
 .244960806271
 .244960806271
 .00699888017917
 .00699888017917
 0
 .00699888017917
 0
 0
 0
 0
 0
 0
 0
 0
 0
 0
 0
 0
 0
 0
 0
 0
 0
 0
 0
 0
 0
 .0297452407615
 .0297452407615
 .0297452407615
 0
 .0297452407615
 0
 0
 0
 .00524916013438
 .00524916013438
 .00524916013438
 .00524916013438
 .00174972004479
 .00174972004479
 .00174972004479
 .00174972004479
 0
 0
 0
 0
 0
 0
 0
 0
 0
 0
 0
 0
 0
 0
 0
 0
 .0174972004479
 .0174972004479
 .0174972004479
 .0174972004479
 .0174972004479
 0
 0
 0
 0
 0
 0
 0
 0
 0
 0
 0
 0
 0
 0
 0
 0
 0
 0
 .00174972004479
 0
 0
 0
 0
 .00174972004479
 .00174972004479
 .00174972004479
 .00174972004479
 .01224804031359
 .0104983202688
 0
 0
 0
 .0104983202688
 .0104983202688
 .0104983202688
 .00174972004479
 .00174972004479
 .00174972004479
 .00174972004479
 0
 0
 0
 0
 0
 0
 0
 0
 0
 0
 0
 0
 0
 0
 0
 0
 0
 0
 0
 0
 0
 0
 0
 0
 0
 0
 0
 0
 0
 0
 0
 0
 0
 0
 0
 0
 0
 0
 0
 0
 0
 0
 0
 0
 0
 0
 0
 .00174972004479
 0
 0
 0
 0
 0
 0
 0
 0
 0
 0
 0
 0
 0
 0
 0
 0
 .00174972004479
 .00174972004479
 .00174972004479
 .00174972004479
 .00174972004479
 0
 0
 0
 0
 0
 0
 0
 0
 0
 0
 0
 0
 0
 0
 0
 0
 .00174972004479
 .00174972004479
 .00174972004479
 .00174972004479
 .00174972004479
 .00174972004479
 .0279955207167
 .0279955207167
 .0279955207167
 .0279955207167
 .0279955207167
 .0279955207167
 0
 0
 0
 0
 0
 0
 0
 0
 0
 0
 0
 0
 0
 65.5305151176076
 65.4832726763982
 .0402435610302
 0
 0
 0
 0
 0
 .0402435610302
 .0402435610302
 .0402435610302
 0
 0
 0
 0
 0
 0
 0
 65.443029115368
 .0857362821948
 .0857362821948
 0
 .0857362821948
 65.3572928331732
 0
 0
 .556410974244
 .556410974244
 64.7098964166
 0
 0
 0
 0
 0
 0
 0
 0
 64.7098964166
 .00524916013438
 .00524916013438
 0
 0
 0
 .0857362821948
 .0857362821948
 0
 0
 0
 0
 0
 0
 0
 0
 0
 0
 0
 0
 0
 0
 0
 0
 0
 0
 .04374300111979
 .04374300111979
 .0402435610302
 .0402435610302
 0
 0
 .0402435610302
 0
 0
 .00349944008959
 0
 0
 .00349944008959
 .00349944008959
 0
 0
 0
 0
 .00349944008959
 .00349944008959
 .00349944008959
 .00349944008959
 .00349944008959
 0
 0
 .00699888017917
 0
 0
 0
 0
 0
 .00699888017917
 .00699888017917
 .00699888017917
 .00699888017917
 .00699888017917
 0
 0
 0
 0
 0
 0
 0
 0
 0
 0
 0
 0
 0
 0
 0
 0
 0
 0
 0
 0
 0
 0
 0
 0
 0
 0
 0
 0
 0
 0
 0
 0
 0
 0
 0
 0
 0
 0
 0
 0
 0
 0
 0
 0
 0
 0
 0
 0
 0
 0
 0
 0
 0
 0
 0
 .0332446808511
 .0332446808511
 .0332446808511
 .0332446808511
 .0332446808511
 .0332446808511
 .0332446808511
