# Supplementary material for: Assessment of biomass potentials of microalgal communities in open pond raceways using mass cultivation
Source: PeerJ. 2020 Jul 16;8:e9418. doi: 10.7717/peerj.9418 (PMC7369025; doi:10.7717/peerj.9418)
Supplement: Data S3 [file peerj-08-9418-s020.zip › Krona/OPR#3/OPR#3_JUN.html]

Javascript must be enabled to view this page.

magnitude
 100.000000000023
 99.9911911089155
 17.287448798322
 1.24792624022
 1.24792624022
 1.24792624022
 1.24792624022
 1.24792624022
 15.9954781025649
 15.9910736570112
 4.18862772157843
 4.18862772157843
 0
 0
 .629835714181
 0
 0
 0
 .00587259407162
 2.296184282
 0
 1.25379883429
 0
 .00293629703581
 0
 0
 0
 0
 0
 0
 0
 0
 0
 0
 0
 0
 0
 0
 0
 0
 0
 0
 0
 11.7833600047
 11.7833600047
 11.7833600047
 0
 0
 0
 .0190859307328
 .0190859307328
 .0190859307328
 0
 0
 0
 0
 0
 0
 0
 0
 0
 0
 0
 0
 0
 0
 0
 0
 0
 0
 0
 0
 0
 0
 0
 0
 0
 .00293629703581
 .00293629703581
 .00293629703581
 0
 .00293629703581
 0
 0
 0
 0
 0
 0
 0
 .0014681485179
 .0014681485179
 .0014681485179
 .0014681485179
 0
 0
 0
 0
 0
 0
 0
 0
 0
 0
 0
 0
 0
 0
 0
 0
 0
 0
 0
 0
 0
 0
 0
 0
 0
 0
 0
 0
 0
 0
 0
 0
 0
 0
 0
 0
 0
 0
 0
 .0117451881432
 0
 0
 0
 0
 .0117451881432
 .0117451881432
 .0117451881432
 .0117451881432
 .0322992673939
 .0322992673939
 0
 0
 0
 .0322992673939
 .0322992673939
 .0322992673939
 0
 0
 0
 0
 0
 0
 0
 0
 0
 0
 0
 0
 0
 0
 0
 0
 0
 0
 0
 0
 0
 0
 0
 0
 0
 0
 0
 0
 0
 0
 0
 0
 0
 0
 0
 0
 0
 0
 0
 0
 0
 0
 0
 0
 0
 0
 0
 0
 0
 0
 0
 0
 0
 0
 0
 0
 0
 0
 0
 0
 0
 0
 0
 0
 0
 0
 0
 0
 0
 0
 0
 0
 0
 0
 0
 0
 0
 0
 0
 0
 0
 0
 0
 0
 0
 0
 0
 0
 0
 .00293629703581
 .00293629703581
 .00293629703581
 .00293629703581
 .00293629703581
 .00293629703581
 .00440444555371
 .0014681485179
 .0014681485179
 .0014681485179
 .0014681485179
 .0014681485179
 .00293629703581
 .00293629703581
 0
 0
 0
 .00293629703581
 .00293629703581
 .00293629703581
 0
 0
 0
 0
 0
 82.6934652709681
 82.5495867162136
 .00734074258952
 0
 0
 0
 0
 0
 0
 0
 0
 .00734074258952
 .00734074258952
 .00734074258952
 0
 0
 0
 0
 82.542245973624
 .0014681485179
 .0014681485179
 0
 .0014681485179
 82.5363733795524
 0
 0
 .325928970975
 .325928970975
 81.9065376653716
 0
 0
 .00293629703581
 0
 0
 0
 .00293629703581
 0
 81.9006650713
 .00293629703581
 .00293629703581
 0
 0
 0
 .30097044617
 .30097044617
 0
 0
 0
 0
 0
 0
 0
 0
 .00440444555371
 0
 0
 .00440444555371
 .00440444555371
 0
 0
 0
 0
 0
 .12626077253971
 .12626077253971
 .121856326986
 .121856326986
 0
 0
 .121856326986
 0
 0
 .00440444555371
 .00293629703581
 .00293629703581
 .0014681485179
 .0014681485179
 0
 0
 0
 0
 .01761778221481
 .01761778221481
 .01761778221481
 .01761778221481
 .014681485179
 .00293629703581
 0
 .00293629703581
 0
 0
 0
 0
 0
 .00293629703581
 .00293629703581
 .00293629703581
 .00293629703581
 .00293629703581
 0
 0
 0
 0
 0
 0
 0
 0
 0
 0
 0
 0
 0
 0
 0
 0
 0
 0
 0
 0
 0
 0
 0
 0
 0
 0
 0
 0
 0
 0
 0
 0
 0
 0
 0
 0
 0
 0
 0
 0
 0
 0
 0
 0
 0
 0
 0
 0
 0
 0
 0
 0
 0
 0
 0
 .00880889110742
 .00880889110742
 .00880889110742
 .00880889110742
 .00880889110742
 .00880889110742
 .00880889110742
