# Supplementary material for: Assessment of biomass potentials of microalgal communities in open pond raceways using mass cultivation
Source: PeerJ. 2020 Jul 16;8:e9418. doi: 10.7717/peerj.9418 (PMC7369025; doi:10.7717/peerj.9418)
Supplement: Data S3 [file peerj-08-9418-s020.zip › Krona/OPR#3/OPR#3_MAY.html]

Javascript must be enabled to view this page.

magnitude
 100.000000000031
 99.988376485661
 7.20076715194528
 0
 0
 0
 0
 0
 7.16153779094798
 7.154273094467
 2.900066835209
 2.900066835209
 0
 0
 2.53683201116
 0
 0
 0
 .151105686804
 .212129137245
 0
 0
 0
 0
 0
 0
 0
 0
 0
 0
 0
 0
 0
 0
 0
 0
 0
 0
 0
 0
 0
 0
 0
 4.13506523697
 4.13506523697
 4.13506523697
 0
 0
 0
 .119141022288
 .119141022288
 .119141022288
 0
 0
 0
 0
 0
 0
 0
 0
 0
 0
 0
 0
 0
 0
 0
 0
 0
 0
 0
 0
 0
 0
 0
 0
 0
 .0014529392962
 .0014529392962
 .0014529392962
 0
 .0014529392962
 0
 0
 0
 0
 0
 0
 0
 .00581175718478
 .00581175718478
 .00581175718478
 .00581175718478
 0
 0
 0
 0
 0
 0
 0
 0
 0
 0
 0
 0
 0
 0
 0
 0
 0
 0
 0
 0
 0
 0
 0
 0
 0
 0
 0
 0
 0
 0
 0
 0
 0
 0
 0
 0
 0
 0
 0
 .0363234824049
 0
 0
 0
 0
 .0363234824049
 .0363234824049
 .0363234824049
 .0363234824049
 .0014529392962
 .0014529392962
 0
 0
 0
 .0014529392962
 .0014529392962
 .0014529392962
 0
 0
 0
 0
 0
 0
 0
 0
 0
 0
 0
 0
 0
 0
 0
 0
 0
 0
 0
 0
 0
 .0014529392962
 0
 0
 0
 0
 .0014529392962
 .0014529392962
 .0014529392962
 .0014529392962
 0
 0
 0
 0
 0
 0
 0
 0
 0
 0
 0
 0
 0
 0
 0
 0
 0
 0
 0
 0
 0
 0
 0
 0
 0
 0
 0
 0
 0
 0
 0
 0
 0
 0
 0
 0
 0
 0
 0
 0
 0
 0
 0
 0
 0
 0
 0
 0
 0
 0
 0
 0
 0
 0
 0
 0
 0
 0
 0
 .00581175718478
 .00581175718478
 .00581175718478
 .00581175718478
 .00581175718478
 .00581175718478
 .0029058785924
 0
 0
 0
 0
 0
 .0014529392962
 .0014529392962
 0
 0
 0
 .0014529392962
 .0014529392962
 .0014529392962
 .0014529392962
 .0014529392962
 .0014529392962
 .0014529392962
 .0014529392962
 92.7396623369412
 92.5885566501364
 .00726469648098
 0
 0
 0
 0
 0
 0
 0
 0
 .00726469648098
 .00726469648098
 .00726469648098
 0
 0
 0
 0
 92.5812919536554
 .00435881788859
 .00435881788859
 0
 .00435881788859
 92.5769331357668
 0
 0
 .32836428094
 .32836428094
 89.2947432656582
 0
 0
 .014529392962
 0
 0
 0
 .0014529392962
 0
 89.2787609334
 .00435881788859
 .00435881788859
 0
 0
 0
 2.94946677128
 2.94946677128
 0
 0
 0
 0
 0
 0
 0
 0
 0
 0
 0
 0
 0
 0
 0
 0
 0
 0
 .10461162932659
 .10461162932659
 .100252811438
 .100252811438
 0
 0
 .100252811438
 0
 0
 .00435881788859
 .00290587859239
 .00290587859239
 .0014529392962
 .0014529392962
 0
 0
 0
 0
 .04649405747828
 .04649405747828
 .04649405747828
 .04649405747828
 .0392293609973
 .00726469648098
 0
 0
 0
 0
 0
 0
 0
 0
 0
 0
 0
 0
 0
 0
 0
 0
 0
 0
 0
 0
 0
 0
 0
 0
 0
 0
 0
 0
 0
 0
 0
 0
 0
 0
 0
 0
 0
 0
 .0392293609973
 0
 0
 0
 0
 0
 .0392293609973
 .0392293609973
 .0392293609973
 0
 0
 .0392293609973
 .0392293609973
 0
 0
 0
 0
 0
 0
 0
 0
 0
 0
 0
 0
 0
 0
 0
 0
 .0116235143696
 .0116235143696
 .0116235143696
 .0116235143696
 .0116235143696
 .0116235143696
 .0116235143696
