# Supplementary material for: Assessment of biomass potentials of microalgal communities in open pond raceways using mass cultivation
Source: PeerJ. 2020 Jul 16;8:e9418. doi: 10.7717/peerj.9418 (PMC7369025; doi:10.7717/peerj.9418)
Supplement: Data S3 [file peerj-08-9418-s020.zip › Krona/OPR#3/OPR#3_NOV.html]

Javascript must be enabled to view this page.

magnitude
 100.000000000011
 99.3752998752629
 23.0975914019441
 .0124748104788
 .0124748104788
 .0124748104788
 .0124748104788
 .0124748104788
 23.0093081277863
 22.7636503214338
 21.6725842049374
 21.6716246041313
 0
 0
 .014394012091
 0
 0
 0
 .000959600806065
 17.7113520775
 0
 3.94108051051
 .000959600806065
 0
 .00287880241819
 0
 0
 0
 0
 .000959600806065
 .000959600806065
 0
 0
 0
 0
 0
 0
 0
 0
 0
 0
 0
 0
 1.08530851166
 1.08530851166
 1.08530851166
 0
 0
 0
 .00575760483639
 .00575760483639
 .00575760483639
 0
 0
 0
 0
 0
 .237980999904
 .237980999904
 .237980999904
 .237980999904
 0
 0
 0
 0
 0
 0
 0
 0
 0
 0
 0
 0
 0
 0
 0
 0
 .00767680644852
 .00575760483639
 .00575760483639
 0
 .00575760483639
 .00191920161213
 .00191920161213
 .00191920161213
 0
 0
 0
 0
 0
 0
 0
 0
 0
 0
 0
 0
 0
 0
 0
 .01535361289704
 .00959600806065
 .00959600806065
 .00959600806065
 .00959600806065
 .00575760483639
 .00575760483639
 .00575760483639
 .00575760483639
 0
 0
 0
 0
 0
 .0470204394971
 .0345456290183
 .0345456290183
 .0345456290183
 .0345456290183
 0
 0
 0
 0
 .0124748104788
 .0124748104788
 .0124748104788
 .0124748104788
 0
 0
 0
 0
 0
 0
 0
 0
 0
 0
 0
 0
 0
 0
 0
 0
 0
 0
 0
 0
 0
 0
 0
 0
 0
 0
 .00767680644852
 .00767680644852
 .00767680644852
 .00767680644852
 .00767680644852
 .00287880241819
 .00287880241819
 .00287880241819
 .00287880241819
 .00287880241819
 0
 0
 0
 0
 0
 0
 0
 .00287880241819
 .00287880241819
 .00287880241819
 .00287880241819
 .00287880241819
 0
 0
 0
 0
 .1266673064005
 .0844448709337
 .0844448709337
 .0844448709337
 .0681316572306
 .0681316572306
 .0163132137031
 .0163132137031
 .0422224354668
 .0422224354668
 .0422224354668
 .0422224354668
 .0422224354668
 0
 0
 0
 0
 0
 0
 0
 0
 .00767680644852
 .00767680644852
 0
 0
 0
 0
 .00767680644852
 .00767680644852
 .00767680644852
 .00767680644852
 0
 0
 0
 0
 0
 0
 0
 0
 0
 0
 0
 0
 .00191920161213
 .00191920161213
 0
 0
 0
 0
 .00191920161213
 .00191920161213
 .00191920161213
 .00191920161213
 0
 0
 0
 0
 0
 0
 0
 0
 0
 0
 0
 0
 .04702043949712
 .0422224354668
 .0422224354668
 .0422224354668
 .0422224354668
 .0422224354668
 0
 0
 0
 0
 0
 0
 0
 0
 .00479800403032
 .00479800403032
 .00479800403032
 .00479800403032
 .00479800403032
 76.0234142597117
 75.1156318971742
 .09596008060643
 .00191920161213
 0
 0
 .00191920161213
 .00191920161213
 .0834852701276
 .0834852701276
 .0834852701276
 0
 0
 0
 .0105556088667
 .0105556088667
 0
 .0105556088667
 74.6128010747967
 .07005085884272
 .07005085884272
 .00479800403032
 .0652528548124
 74.5321946070873
 .0383840322426
 .0383840322426
 .790711064197
 .790711064197
 73.3749160349737
 .0134344112849
 0
 0
 .00191920161213
 .256213415219
 .000959600806065
 .0211112177334
 .00287880241819
 73.0783993859
 0
 0
 .22550618942513
 .223586987813
 .00191920161213
 0
 0
 .10267728624889
 .00575760483639
 .0969196814125
 .01055560886671
 .00383840322426
 .00383840322426
 .00671720564245
 .00671720564245
 0
 0
 0
 0
 0
 .406870741771
 .406870741771
 .406870741771
 .406870741771
 0
 .9077823625376
 .8885903464163
 .8885903464163
 .0930812781883
 .0930812781883
 0
 0
 .795509068228
 .795509068228
 0
 0
 0
 0
 0
 .0191920161213
 .0191920161213
 .0191920161213
 .0191920161213
 0
 0
 0
 0
 0
 0
 0
 .00287880241819
 .00287880241819
 .00287880241819
 .00287880241819
 .00287880241819
 .00287880241819
 0
 0
 0
 0
 0
 0
 0
 0
 0
 0
 0
 0
 0
 0
 0
 .06333365320019
 .0259092217637
 .0259092217637
 .0259092217637
 .0259092217637
 .0259092217637
 .00287880241819
 .00287880241819
 .00287880241819
 .00287880241819
 .00287880241819
 .0345456290183
 .0345456290183
 .0345456290183
 .0345456290183
 .0345456290183
 .004798004030325
 0
 0
 0
 0
 0
 .000959600806065
 .000959600806065
 .000959600806065
 0
 0
 .000959600806065
 .000959600806065
 0
 0
 .00383840322426
 .00383840322426
 0
 0
 0
 .00383840322426
 .00383840322426
 .00383840322426
 0
 0
 0
 0
 0
 0
 .624700124748
 .624700124748
 .624700124748
 .624700124748
 .624700124748
 .624700124748
 .624700124748
