# Supplementary material for: Assessment of biomass potentials of microalgal communities in open pond raceways using mass cultivation
Source: PeerJ. 2020 Jul 16;8:e9418. doi: 10.7717/peerj.9418 (PMC7369025; doi:10.7717/peerj.9418)
Supplement: Data S3 [file peerj-08-9418-s020.zip › Krona/OPR#3/OPR#3_OCT.html]

Javascript must be enabled to view this page.

magnitude
 100.000000000072
 98.3181433807016
 23.6117636005188
 .0125277960475
 .0125277960475
 .0125277960475
 .0125277960475
 .0125277960475
 23.3987910677118
 23.3893952206762
 23.3643396285812
 23.3643396285812
 0
 0
 .00939584703561
 0
 0
 0
 .00626389802374
 13.7649159072
 0
 9.58063202731
 0
 0
 .00313194901187
 0
 0
 0
 0
 0
 0
 0
 0
 0
 0
 0
 0
 0
 0
 0
 0
 0
 0
 .0125277960475
 .0125277960475
 .0125277960475
 0
 0
 0
 .0125277960475
 .0125277960475
 .0125277960475
 0
 0
 0
 0
 0
 .00313194901187
 .00313194901187
 .00313194901187
 .00313194901187
 0
 0
 0
 0
 0
 0
 0
 0
 0
 0
 0
 0
 .00626389802374
 .00626389802374
 .00626389802374
 .00626389802374
 0
 0
 0
 0
 0
 0
 0
 0
 0
 0
 0
 0
 0
 0
 0
 0
 0
 0
 0
 0
 0
 0
 0
 0
 0
 0
 0
 0
 0
 0
 0
 0
 0
 0
 0
 0
 0
 .1847849917001
 .162861348617
 .162861348617
 .162861348617
 .162861348617
 0
 0
 0
 0
 .0219236430831
 .0219236430831
 .0219236430831
 .0219236430831
 0
 0
 0
 0
 0
 .0156597450594
 0
 0
 0
 0
 .0156597450594
 .0156597450594
 .0156597450594
 .0156597450594
 0
 0
 0
 0
 0
 0
 0
 0
 0
 0
 0
 0
 0
 0
 0
 0
 0
 0
 0
 0
 0
 0
 0
 0
 0
 0
 0
 0
 0
 0
 0
 0
 0
 0
 0
 0
 0
 0
 .0187916940712
 0
 0
 0
 0
 0
 0
 0
 .0187916940712
 .0187916940712
 .0187916940712
 .0187916940712
 .0187916940712
 0
 0
 0
 0
 0
 0
 0
 0
 0
 0
 0
 0
 0
 0
 0
 0
 0
 0
 0
 0
 0
 0
 0
 0
 0
 0
 0
 0
 0
 0
 0
 0
 0
 0
 0
 0
 0
 0
 0
 0
 0
 0
 0
 0
 0
 0
 0
 0
 0
 0
 0
 0
 .1221460114629
 .0375833881424
 .0375833881424
 .0375833881424
 .0375833881424
 .0375833881424
 0
 0
 0
 0
 0
 0
 0
 0
 .0845626233205
 .0845626233205
 .0845626233205
 .0845626233205
 .0845626233205
 73.6101976260282
 73.2500234896638
 .0814306743087
 0
 0
 0
 0
 0
 .0657709292493
 .0657709292493
 .0657709292493
 0
 0
 0
 .0156597450594
 .0156597450594
 0
 .0156597450594
 73.099689937094
 .0689028782612
 .0689028782612
 .0532431332018
 .0156597450594
 73.0245231608091
 .00626389802374
 .00626389802374
 .419681167591
 .419681167591
 72.5515988600163
 .0219236430831
 0
 0
 0
 .43534091265
 .0156597450594
 .00626389802374
 0
 72.0724106612
 0
 0
 .0438472861662
 .0438472861662
 0
 0
 0
 .00313194901187
 .00313194901187
 0
 .00626389802374
 0
 0
 .00626389802374
 .00626389802374
 0
 0
 0
 0
 0
 .0689028782611
 .0689028782611
 .0689028782611
 .0689028782611
 0
 .3601741363644
 .322590748222
 .322590748222
 .162861348617
 .162861348617
 0
 0
 .159729399605
 .159729399605
 0
 0
 0
 0
 0
 .0375833881424
 .0375833881424
 .0375833881424
 .0375833881424
 0
 0
 0
 0
 0
 0
 0
 .00313194901187
 0
 0
 0
 0
 0
 .00313194901187
 .00313194901187
 .00313194901187
 .00313194901187
 .00313194901187
 0
 0
 0
 0
 0
 0
 0
 0
 0
 0
 0
 0
 0
 0
 0
 0
 0
 0
 0
 0
 0
 0
 0
 0
 0
 0
 .95211249960861
 0
 0
 0
 0
 0
 .00939584703561
 .00939584703561
 .00939584703561
 0
 0
 0
 0
 .00939584703561
 .00939584703561
 .942716652573
 .942716652573
 0
 0
 0
 .942716652573
 .942716652573
 .942716652573
 0
 0
 0
 0
 0
 0
 1.68185661937
 1.68185661937
 1.68185661937
 1.68185661937
 1.68185661937
 1.68185661937
 1.68185661937
