# Supplementary material for: Assessment of biomass potentials of microalgal communities in open pond raceways using mass cultivation
Source: PeerJ. 2020 Jul 16;8:e9418. doi: 10.7717/peerj.9418 (PMC7369025; doi:10.7717/peerj.9418)
Supplement: Data S3 [file peerj-08-9418-s020.zip › Krona/OPR#3/OPR#3_SEP.html]

Javascript must be enabled to view this page.

magnitude
 99.9999999999559
 99.8071757007979
 27.5854112760544
 .206008866622
 .206008866622
 .206008866622
 .206008866622
 .206008866622
 12.060583087494
 12.0292697397676
 12.0193813141697
 12.0193813141697
 0
 0
 1.08113453203
 0
 .00824035466486
 0
 .00659228373189
 7.75417373964
 0
 3.16759233317
 0
 0
 .00164807093297
 0
 0
 0
 0
 0
 0
 0
 0
 0
 0
 0
 0
 0
 0
 0
 0
 0
 0
 .00659228373189
 .00659228373189
 .00659228373189
 0
 0
 0
 .00329614186595
 .00329614186595
 .00329614186595
 0
 0
 0
 0
 0
 .00494421279892
 .00494421279892
 .00494421279892
 .00494421279892
 0
 0
 0
 0
 0
 0
 0
 0
 0
 0
 0
 0
 .0230729930616
 .0230729930616
 .0230729930616
 .0230729930616
 .00329614186595
 0
 0
 0
 0
 .00329614186595
 .00329614186595
 .00329614186595
 0
 0
 0
 0
 0
 0
 0
 0
 0
 0
 0
 0
 0
 0
 0
 0
 0
 0
 0
 0
 0
 0
 0
 0
 0
 0
 0
 0
 0
 15.2858579032789
 4.95245315358
 4.95245315358
 4.95245315358
 4.95245315358
 .00494421279892
 .00494421279892
 .00494421279892
 .00494421279892
 10.3284605369
 10.3284605369
 10.3284605369
 10.3284605369
 0
 0
 0
 0
 0
 .00164807093297
 0
 0
 0
 0
 .00164807093297
 .00164807093297
 .00164807093297
 .00164807093297
 .00329614186595
 0
 0
 0
 0
 0
 0
 0
 .00329614186595
 .00329614186595
 .00329614186595
 .00329614186595
 0
 0
 0
 0
 0
 0
 0
 0
 0
 0
 .02801720586052
 .02801720586052
 .02801720586052
 .0230729930616
 .0230729930616
 .00494421279892
 .00494421279892
 0
 0
 0
 0
 0
 0
 0
 0
 0
 .00329614186594
 0
 0
 0
 0
 0
 0
 0
 .00329614186594
 .00164807093297
 .00164807093297
 .00164807093297
 .00164807093297
 .00164807093297
 .00164807093297
 .00164807093297
 .00164807093297
 0
 0
 0
 0
 0
 0
 0
 0
 0
 0
 0
 0
 0
 0
 0
 0
 0
 0
 0
 0
 0
 0
 0
 0
 0
 0
 0
 0
 0
 0
 0
 0
 0
 0
 0
 0
 0
 0
 0
 0
 0
 0
 0
 0
 0
 0
 0
 0
 .09723618504537
 .0955881141124
 .0955881141124
 .0955881141124
 .0955881141124
 .0955881141124
 0
 0
 0
 0
 0
 0
 0
 0
 .00164807093297
 .00164807093297
 .00164807093297
 .00164807093297
 .00164807093297
 70.7121314501422
 70.2243024539822
 .09064390131352
 0
 0
 0
 0
 0
 .0856996885146
 .0856996885146
 .0856996885146
 0
 0
 0
 .00494421279892
 .00494421279892
 .00494421279892
 0
 70.0743279990816
 .45157143563489
 .45157143563489
 .00659228373189
 .444979151903
 69.6211084925137
 .00659228373189
 .00659228373189
 .159862880498
 .159862880498
 69.4315803352222
 .0329614186595
 .00164807093297
 0
 0
 2.6039520741
 .00659228373189
 .00988842559784
 0
 66.7765380622
 0
 0
 .0181287802627
 .0181287802627
 0
 0
 0
 .00494421279892
 .00494421279892
 0
 .00164807093297
 0
 0
 .00164807093297
 .00164807093297
 0
 0
 0
 0
 0
 .05933055358707
 .05933055358707
 .05933055358707
 .0576824826541
 .00164807093297
 .44003493910381
 .43344265537192
 .43344265537192
 .428498442573
 .428498442573
 0
 0
 .00494421279892
 .00494421279892
 0
 0
 0
 0
 0
 .00659228373189
 .00659228373189
 .00659228373189
 .00659228373189
 .0477940570562
 .0477940570562
 .0477940570562
 .0477940570562
 0
 0
 .0477940570562
 0
 0
 0
 0
 0
 0
 0
 0
 0
 0
 0
 0
 0
 0
 0
 0
 0
 0
 0
 0
 0
 0
 0
 0
 0
 0
 0
 0
 0
 0
 0
 0
 0
 0
 0
 0
 0
 1.40910064769
 0
 0
 0
 0
 0
 0
 0
 0
 0
 0
 0
 0
 0
 0
 1.40910064769
 1.40910064769
 0
 0
 0
 1.40910064769
 1.40910064769
 1.40910064769
 0
 0
 0
 0
 0
 0
 .192824299158
 .192824299158
 .192824299158
 .192824299158
 .192824299158
 .192824299158
 .192824299158
