# Supplementary material for: Assessment of biomass potentials of microalgal communities in open pond raceways using mass cultivation
Source: PeerJ. 2020 Jul 16;8:e9418. doi: 10.7717/peerj.9418 (PMC7369025; doi:10.7717/peerj.9418)
Supplement: Data S3 [file peerj-08-9418-s020.zip › Krona/OPR#3.html]

Javascript must be enabled to view this page.

magnitude
 100.00000000001
 99.6736726609954
 22.8357224569791
 .262435064352188
 .262435064352188
 .262435064352188
 .262435064352188
 .262435064352188
 20.2820778230118
 20.2100557701241
 11.4199073104259
 11.4140374079149
 7.1950728141375E-04
 .640562445072864
 2.1871500559875E-04
 2.32802026082625E-03
 2.1137359013875E-04
 2.17793465254019E-02
 6.22338317360912
 3.5975364070625E-04
 4.52281201789764
 1.19950100758125E-04
 .000585752135075
 9.5735279537875E-04
 3.5975364070625E-04
 3.5975364070625E-04
 1.19950100758125E-04
 1.19950100758125E-04
 5.39019876948125E-03
 5.39019876948125E-03
 7.1950728141375E-04
 7.1950728141375E-04
 7.1950728141375E-04
 8.7113024643103
 8.7113024643103
 8.7113024643103
 .077251628084075
 .077251628084075
 .077251628084075
 8.7486002239625E-04
 8.7486002239625E-04
 8.7486002239625E-04
 3.07571452143488E-02
 3.07571452143488E-02
 3.07571452143488E-02
 3.07571452143488E-02
 .015829160191125
 .015829160191125
 .015829160191125
 .015829160191125
 3.6671113856675E-03
 3.6671113856675E-03
 3.6671113856675E-03
 3.6671113856675E-03
 .007922937099375
 .007271019164615
 .007271019164615
 .007271019164615
 .00065191793476
 .00065191793476
 .00065191793476
 6.561450167975E-04
 6.561450167975E-04
 6.561450167975E-04
 6.561450167975E-04
 1.21326860297725E-02
 1.21326860297725E-02
 1.21326860297725E-02
 1.21326860297725E-02
 1.05686795069125E-03
 1.05686795069125E-03
 1.05686795069125E-03
 1.05686795069125E-03
 .00191920161213
 1.19950100758125E-03
 1.19950100758125E-03
 1.19950100758125E-03
 1.19950100758125E-03
 7.1970060454875E-04
 7.1970060454875E-04
 7.1970060454875E-04
 7.1970060454875E-04
 2.1871500559875E-03
 2.1871500559875E-03
 2.1871500559875E-03
 2.1871500559875E-03
 2.1871500559875E-03
 2.15922063117979
 .643732516401912
 .643732516401912
 .643732516401912
 .643732516401912
 .000618026599865
 .000618026599865
 .000618026599865
 .000618026599865
 1.51487008817801
 1.51487008817801
 1.51487008817801
 1.51487008817801
 8.3907758231575E-03
 8.3907758231575E-03
 8.3907758231575E-03
 8.3907758231575E-03
 8.3907758231575E-03
 .112649126257869
 .111298886237613
 .111298886237613
 .111298886237613
 .111298886237613
 1.35024002025625E-03
 1.35024002025625E-03
 1.35024002025625E-03
 1.35024002025625E-03
 .000959600806065
 .000959600806065
 .000959600806065
 .000959600806065
 .000959600806065
 3.5985030227375E-04
 3.5985030227375E-04
 3.5985030227375E-04
 3.5985030227375E-04
 3.5985030227375E-04
 .003502150732565
 .003502150732565
 .003502150732565
 .0028841241327
 .0028841241327
 .000618026599865
 .000618026599865
 2.02108284526125E-03
 3.5985030227375E-04
 3.5985030227375E-04
 3.5985030227375E-04
 3.5985030227375E-04
 1.6612325429875E-03
 1.6612325429875E-03
 1.6612325429875E-03
 1.6612325429875E-03
 .018594392792205
 1.05556088667125E-02
 1.05556088667125E-02
 1.05556088667125E-02
 .008516457153825
 .008516457153825
 2.0391517128875E-03
 2.0391517128875E-03
 8.0387839254925E-03
 7.83277505887125E-03
 7.83277505887125E-03
 7.83277505887125E-03
 7.83277505887125E-03
 2.0600886662125E-04
 2.0600886662125E-04
 2.0600886662125E-04
 2.0600886662125E-04
 3.69659129661375E-03
 .000959600806065
 .000959600806065
 .000959600806065
 .000959600806065
 .000959600806065
 2.73699049054875E-03
 2.73699049054875E-03
 2.73699049054875E-03
 2.73699049054875E-03
 2.73699049054875E-03
 2.3990020151625E-04
 2.3990020151625E-04
 2.3990020151625E-04
 2.3990020151625E-04
 2.3990020151625E-04
 2.3990020151625E-04
 1.3122217831725E-03
 1.3122217831725E-03
 1.3122217831725E-03
 1.3122217831725E-03
 1.3122217831725E-03
 1.3122217831725E-03
 5.08760647641638E-02
 3.87697055251638E-02
 3.87697055251638E-02
 3.87697055251638E-02
 3.87697055251638E-02
 3.87697055251638E-02
 5.4865454150125E-04
 5.4865454150125E-04
 5.4865454150125E-04
 5.4865454150125E-04
 5.4865454150125E-04
 1.15577046974988E-02
 1.15577046974988E-02
 1.15577046974988E-02
 1.15577046974988E-02
 1.15577046974988E-02
 76.4477148569483
 76.1157870657353
 .052097969355925
 2.3990020151625E-04
 2.3990020151625E-04
 2.3990020151625E-04
 3.94364820851125E-02
 3.94364820851125E-02
 3.94364820851125E-02
 8.52664122866875E-03
 8.52664122866875E-03
 8.52664122866875E-03
 3.8949458406275E-03
 3.8949458406275E-03
 .000618026599865
 3.2769192407625E-03
 75.996801074677
 .09228535245965
 .09228535245965
 8.07917762050125E-03
 8.42061748391487E-02
 75.9001547111928
 6.40502674977875E-03
 6.40502674977875E-03
 .470889280529875
 .470889280529875
 74.1500621431853
 .010338702331975
 2.0600886662125E-04
 8.31304536373875E-03
 2.3990020151625E-04
 .411938300246125
 2.90145369966938E-03
 5.20659721087375E-03
 3.5985030227375E-04
 73.7105582849625
 2.1391616131925E-03
 2.1391616131925E-03
 .03635802916203
 3.56953817802375E-02
 6.626473817925E-04
 1.22045688894516
 1.22045688894516
 .01384418100746
 1.7292208308975E-03
 1.21149601765625E-02
 2.3084472279275E-03
 4.798004030325E-04
 4.798004030325E-04
 .001828646824895
 .001828646824895
 .00205256379661
 .00107926092212
 .00107926092212
 .00097330287449
 .00097330287449
 6.68880217023963E-02
 6.68880217023963E-02
 6.68880217023963E-02
 .066682012835775
 2.0600886662125E-04
 .297336246533939
 .28941528553449
 .287671074002865
 .167648238923875
 8.55551336722875E-02
 8.20931052515875E-02
 .12002283507899
 .12002283507899
 .001744211531625
 .000730271953525
 .000730271953525
 .0010139395781
 .0010139395781
 7.92096099944875E-03
 7.92096099944875E-03
 7.92096099944875E-03
 7.92096099944875E-03
 3.45915446790363E-02
 3.45915446790363E-02
 3.45915446790363E-02
 3.45915446790363E-02
 .025228427456025
 3.38886009098625E-03
 .005974257132025
 .005505390976695
 3.5985030227375E-04
 3.5985030227375E-04
 3.5985030227375E-04
 3.5985030227375E-04
 3.5985030227375E-04
 5.14554067442125E-03
 5.14554067442125E-03
 5.14554067442125E-03
 5.14554067442125E-03
 5.14554067442125E-03
 7.91670665002375E-03
 3.2386527204625E-03
 3.2386527204625E-03
 3.2386527204625E-03
 3.2386527204625E-03
 3.2386527204625E-03
 3.5985030227375E-04
 3.5985030227375E-04
 3.5985030227375E-04
 3.5985030227375E-04
 3.5985030227375E-04
 4.3182036272875E-03
 4.3182036272875E-03
 4.3182036272875E-03
 4.3182036272875E-03
 4.3182036272875E-03
 .302094078603606
 .00107926092212
 .00107926092212
 .00107926092212
 .00107926092212
 .00107926092212
 6.55785474557812E-03
 6.55785474557812E-03
 6.55785474557812E-03
 5.02362022542062E-03
 5.02362022542062E-03
 1.5342345201575E-03
 1.5342345201575E-03
 .294456962935908
 .294456962935908
 .294456962935908
 .294456962935908
 .294456962935908
 .326327339014653
 .326327339014653
 .326327339014653
 .326327339014653
 .326327339014653
 .326327339014653
 .326327339014653
