# Supplementary material for: Assessment of biomass potentials of microalgal communities in open pond raceways using mass cultivation
Source: PeerJ. 2020 Jul 16;8:e9418. doi: 10.7717/peerj.9418 (PMC7369025; doi:10.7717/peerj.9418)
Supplement: Data S5 [file peerj-08-9418-s022.zip › Krona/OPR#1/OPR#1_APR.html]

Javascript must be enabled to view this page.

magnitude
 98.9896002760701
 81.0611661491701
 48.6716940214257
 10.5697668687454
 1.6437478436592
 .332692592045
 .332692592045
 .332692592045
 .1897579969438
 .113361920252
 .113361920252
 .0763960766918
 .0763960766918
 .152792153384
 .152792153384
 .152792153384
 .261225294495
 .261225294495
 .261225294495
 .7072798067914
 .147863374242
 .147863374242
 .512593030706
 .512593030706
 .0468234018434
 .0468234018434
 6.308837301
 .0566809601262
 .0566809601262
 .0566809601262
 6.2521563408738
 .0763960766918
 .0763960766918
 .147863374242
 .147863374242
 6.02789688994
 6.02789688994
 1.9370102025718
 .9512543742908
 .9512543742908
 .0517521809848
 .229188230075
 .42141061659
 .118290699394
 .130612647247
 .480555966287
 .480555966287
 .480555966287
 .505199861994
 .505199861994
 .505199861994
 .192222386515
 .192222386515
 .192222386515
 .192222386515
 .4879491349994
 .4879491349994
 .441125733156
 .441125733156
 .0468234018434
 .0468234018434
 10.934496525213
 10.525407856476
 9.507614963776
 2.0947311351
 2.0947311351
 1.98876238356
 1.98876238356
 5.01749716595
 5.01749716595
 .406624279166
 .406624279166
 1.0177928927
 .56188082212
 .56188082212
 .189757996944
 .189757996944
 .266154073636
 .266154073636
 .28094041106
 .28094041106
 .28094041106
 .28094041106
 .128148257677
 .128148257677
 .128148257677
 .128148257677
 .219330671793
 .219330671793
 .219330671793
 .219330671793
 .219330671793
 26.8248804771393
 .704815417221
 .704815417221
 .704815417221
 .704815417221
 .0566809601262
 .0566809601262
 .0566809601262
 .0566809601262
 25.725762728606
 .512593030706
 .512593030706
 .512593030706
 25.2131696979
 25.2131696979
 25.2131696979
 .3376213711861
 .239045788358
 .239045788358
 .239045788358
 .0985755828281
 .0985755828281
 .0985755828281
 .123219478535
 .123219478535
 .123219478535
 .123219478535
 .123219478535
 .288333579772
 .288333579772
 .288333579772
 .288333579772
 .288333579772
 .288333579772
 20.661442160755
 .483020355858
 .483020355858
 .483020355858
 .483020355858
 .483020355858
 4.044063285527
 4.044063285527
 3.822268224164
 .155256542954
 .155256542954
 3.66701168121
 3.66701168121
 .221795061363
 .221795061363
 .221795061363
 16.13435851937
 12.733500911803
 11.341120804352
 .113361920252
 .113361920252
 11.2277588841
 11.2277588841
 1.392380107451
 1.392380107451
 .179900438661
 1.21247966879
 .970969490857
 .970969490857
 .904430972448
 .904430972448
 .066538518409
 .066538518409
 2.42988811671
 2.42988811671
 2.42988811671
 2.42988811671
 7.070333678356
 3.5413278131
 3.5413278131
 3.5413278131
 3.5413278131
 3.5413278131
 3.529005865256
 3.529005865256
 2.08980235596
 .330228202474
 .330228202474
 .125683868106
 .125683868106
 1.63389028538
 1.63389028538
 1.439203509296
 1.2790181872
 1.2790181872
 .160185322096
 .160185322096
 .2464389570706
 .142934595101
 .142934595101
 .142934595101
 .142934595101
 .142934595101
 .1035043619696
 .1035043619696
 .0492877914141
 .0492877914141
 .0492877914141
 .0542165705555
 .0542165705555
 .0542165705555
 .9512543742911
 .9512543742911
 .4731627975751
 .3499433190401
 .0492877914141
 .0492877914141
 .300655527626
 .300655527626
 .123219478535
 .123219478535
 .123219478535
 .041894622702
 .041894622702
 .041894622702
 .041894622702
 .436196954014
 .436196954014
 .436196954014
 .436196954014
 .3967667208834
 .3967667208834
 .3967667208834
 .3967667208834
 .3967667208834
 .0468234018434
 .34994331904
 2.7749026566163
 2.7749026566163
 .6530632362363
 .613633003105
 .613633003105
 .613633003105
 .0394302331313
 .0394302331313
 .0394302331313
 2.12183942038
 2.12183942038
 2.12183942038
 2.12183942038
 17.9284341269
 17.9284341269
 17.9284341269
 17.9284341269
 17.9284341269
 17.9284341269
 17.9284341269
