# Supplementary material for: Assessment of biomass potentials of microalgal communities in open pond raceways using mass cultivation
Source: PeerJ. 2020 Jul 16;8:e9418. doi: 10.7717/peerj.9418 (PMC7369025; doi:10.7717/peerj.9418)
Supplement: Data S5 [file peerj-08-9418-s022.zip › Krona/OPR#1/OPR#1_AUG.html]

Javascript must be enabled to view this page.

magnitude
 72.2439153282864
 56.7540588942864
 10.3255923196564
 3.29996183317675
 .3581809107181
 .132115909691
 .132115909691
 .132115909691
 .143859546108
 .143859546108
 .143859546108
 0
 0
 .0322950001468
 .0322950001468
 .0322950001468
 0
 0
 0
 .0499104547723
 0
 0
 .0499104547723
 .0499104547723
 0
 0
 .3346936378848
 0
 0
 0
 .3346936378848
 .0675259093978
 .0675259093978
 0
 0
 .267167728487
 .267167728487
 1.30647955139385
 1.11564545961685
 1.11564545961685
 .114500455066
 .0763336367106
 .921875458736
 0
 .00293590910425
 0
 0
 0
 .190834091777
 .190834091777
 .190834091777
 1.30060773318
 1.30060773318
 1.30060773318
 1.30060773318
 0
 0
 0
 0
 0
 0
 3.229500014675
 1.673468189425
 .769208185315
 0
 0
 .769208185315
 .769208185315
 0
 0
 0
 0
 .90426000411
 .869029094859
 .869029094859
 0
 0
 .035230909251
 .035230909251
 0
 0
 0
 0
 1.55603182525
 1.55603182525
 1.55603182525
 1.55603182525
 0
 0
 0
 0
 0
 2.7891136490446
 .0411027274596
 .0411027274596
 .0411027274596
 .0411027274596
 0
 0
 0
 0
 2.17844455536
 0
 0
 0
 2.17844455536
 2.17844455536
 2.17844455536
 .569566366225
 .217257273715
 .217257273715
 .217257273715
 .35230909251
 .35230909251
 .35230909251
 1.00701682276
 1.00701682276
 1.00701682276
 1.00701682276
 1.00701682276
 .126244091483
 .126244091483
 .126244091483
 .126244091483
 .126244091483
 .126244091483
 21.7404069170006
 .593053639059
 .593053639059
 .593053639059
 .593053639059
 .593053639059
 17.4627873521
 17.4627873521
 4.0691700185
 0
 0
 4.0691700185
 4.0691700185
 13.3936173336
 13.3936173336
 13.3936173336
 3.6845659258416
 .1115645459616
 .1115645459616
 .023487272834
 .023487272834
 .0880772731276
 .0880772731276
 0
 0
 0
 0
 0
 0
 0
 0
 0
 0
 3.57300137988
 3.57300137988
 3.57300137988
 3.57300137988
 2.116790464167
 .428642729221
 .428642729221
 .428642729221
 .428642729221
 .428642729221
 1.688147734946
 1.688147734946
 1.315287278706
 .273039546696
 .273039546696
 .6899386395
 .6899386395
 .35230909251
 .35230909251
 .37286045624
 .140923637004
 .140923637004
 .231936819236
 .231936819236
 .8514136402333
 .0264231819383
 .0264231819383
 .0264231819383
 .0264231819383
 .0264231819383
 .824990458295
 .824990458295
 .824990458295
 .824990458295
 .824990458295
 0
 0
 0
 .1262440914829
 .1262440914829
 .1115645459616
 .0792695458148
 .0616540911893
 .0616540911893
 .0176154546255
 .0176154546255
 .0322950001468
 .0322950001468
 .0322950001468
 .0146795455213
 .0146795455213
 .0146795455213
 .0146795455213
 0
 0
 0
 0
 1.887789554031
 1.887789554031
 1.887789554031
 1.887789554031
 1.887789554031
 1.41804409735
 .469745456681
 19.5795778162323
 19.5795778162323
 .2290009101323
 .202577728194
 .202577728194
 .202577728194
 .0264231819383
 .0264231819383
 .0264231819383
 19.3505769061
 19.3505769061
 19.3505769061
 19.3505769061
 15.489856434
 15.489856434
 15.489856434
 15.489856434
 15.489856434
 15.489856434
 15.489856434
