# Supplementary material for: Assessment of biomass potentials of microalgal communities in open pond raceways using mass cultivation
Source: PeerJ. 2020 Jul 16;8:e9418. doi: 10.7717/peerj.9418 (PMC7369025; doi:10.7717/peerj.9418)
Supplement: Data S5 [file peerj-08-9418-s022.zip › Krona/OPR#1/OPR#1_JUL.html]

Javascript must be enabled to view this page.

magnitude
 87.2066159096289
 82.5728537673389
 41.3783145182333
 8.75820425307922
 .2546600157523
 .0131268049357
 .0131268049357
 .0131268049357
 .152270937254
 .152270937254
 .152270937254
 0
 0
 .0446311367813
 .0446311367813
 .0446311367813
 0
 0
 0
 .0446311367813
 0
 0
 .0446311367813
 .0446311367813
 0
 0
 .52244683644031
 0
 0
 0
 .52244683644031
 .0761354686269
 .0761354686269
 .00787608296141
 .00787608296141
 .438435284852
 .438435284852
 3.07167235494661
 2.50984510369961
 2.50984510369961
 .406930953006
 .267786820688
 1.80362299816
 .00787608296141
 .0236282488842
 0
 0
 0
 .561827251247
 .561827251247
 .561827251247
 4.90942504594
 4.90942504594
 4.90942504594
 4.90942504594
 0
 0
 0
 0
 0
 0
 1.34155946442334
 .24415857180334
 .0236282488842
 0
 0
 .0236282488842
 .0236282488842
 0
 0
 0
 0
 .22053032291914
 .217904961932
 .217904961932
 0
 0
 .00262536098714
 .00262536098714
 0
 0
 0
 0
 1.09740089262
 1.09740089262
 1.09740089262
 1.09740089262
 0
 0
 0
 0
 0
 31.0711472827467
 .0131268049357
 .0131268049357
 .0131268049357
 .0131268049357
 .00262536098714
 .00262536098714
 .00262536098714
 .00262536098714
 30.5093200315
 0
 0
 0
 30.5093200315
 30.5093200315
 30.5093200315
 .5460750853239
 .451562089787
 .451562089787
 .451562089787
 .0945129955369
 .0945129955369
 .0945129955369
 .207403517984
 .207403517984
 .207403517984
 .207403517984
 .207403517984
 .141769493305
 .141769493305
 .141769493305
 .141769493305
 .141769493305
 .141769493305
 31.6408506169206
 15.2874770281
 15.2874770281
 15.2874770281
 15.2874770281
 15.2874770281
 15.95956944075
 15.95956944075
 14.4394854292
 0
 0
 14.4394854292
 14.4394854292
 1.52008401155
 1.52008401155
 1.52008401155
 .39380414807061
 .38067734313491
 .3728012601735
 .362299816225
 .362299816225
 .0105014439485
 .0105014439485
 .00787608296141
 .00787608296141
 .00787608296141
 0
 0
 0
 0
 0
 0
 0
 .0131268049357
 .0131268049357
 .0131268049357
 .0131268049357
 3.84877920714424
 2.61485954319
 2.61485954319
 2.61485954319
 2.61485954319
 2.61485954319
 1.23391966395424
 1.23391966395424
 1.2181674980314
 .0262536098714
 .0262536098714
 .771856130218
 .771856130218
 .420057757942
 .420057757942
 .01575216592284
 .00262536098714
 .00262536098714
 .0131268049357
 .0131268049357
 .847991598845
 .829614071935
 .829614071935
 .829614071935
 .829614071935
 .829614071935
 .01837752691
 .01837752691
 .01837752691
 .01837752691
 .01837752691
 0
 0
 0
 .4331845628776
 .4331845628776
 .1207666054083
 .0945129955369
 .0262536098714
 .0262536098714
 .0682593856655
 .0682593856655
 .0262536098714
 .0262536098714
 .0262536098714
 .228406405881
 .228406405881
 .228406405881
 .228406405881
 .0840115515883
 .0840115515883
 .0840115515883
 .0840115515883
 1.247046468889
 1.247046468889
 1.247046468889
 1.247046468889
 1.247046468889
 .472564977684
 .774481491205
 3.0349173011241
 3.0349173011241
 .0787608296141
 .0131268049357
 .0131268049357
 .0131268049357
 .0656340246784
 .0656340246784
 .0656340246784
 2.95615647151
 2.95615647151
 2.95615647151
 2.95615647151
 4.63376214229
 4.63376214229
 4.63376214229
 4.63376214229
 4.63376214229
 4.63376214229
 4.63376214229
