# Supplementary material for: Assessment of biomass potentials of microalgal communities in open pond raceways using mass cultivation
Source: PeerJ. 2020 Jul 16;8:e9418. doi: 10.7717/peerj.9418 (PMC7369025; doi:10.7717/peerj.9418)
Supplement: Data S5 [file peerj-08-9418-s022.zip › Krona/OPR#1/OPR#1_JUN.html]

Javascript must be enabled to view this page.

magnitude
 90.2287699666106
 86.8761844598906
 57.0007219565102
 8.1648768161739
 .30683151340155
 .133110730079
 .133110730079
 .133110730079
 .03609782510605
 .0315855969678
 .0315855969678
 .00451222813825
 .00451222813825
 .110549589387
 .110549589387
 .110549589387
 0
 0
 0
 .0270733688295
 0
 0
 .0270733688295
 .0270733688295
 0
 0
 5.1710134464398
 .018048912553
 .018048912553
 .018048912553
 5.1529645338868
 .0857323346268
 .0857323346268
 .20756249436
 .20756249436
 4.8596697049
 4.8596697049
 2.4343470805904
 1.9808681526954
 1.9808681526954
 .0473783954517
 .399332190236
 1.39879072286
 .0541467376591
 .0812201064886
 0
 0
 0
 .453478927895
 .453478927895
 .453478927895
 .214330836567
 .214330836567
 .214330836567
 .214330836567
 .03835393917515
 .03835393917515
 .0338417110369
 .0338417110369
 .00451222813825
 .00451222813825
 1.03330024366068
 .78287158198768
 .1331107300785
 0
 0
 .0744517642812
 .0744517642812
 .0586589657973
 .0586589657973
 0
 0
 .64976085190918
 .629455825287
 .629455825287
 .0135366844148
 .0135366844148
 .00676834220738
 .00676834220738
 0
 0
 0
 0
 .250428661673
 .250428661673
 .250428661673
 .250428661673
 .00225611406913
 .00225611406913
 .00225611406913
 .00225611406913
 .00225611406913
 47.2069307824265
 .00902445627651
 .00902445627651
 .00902445627651
 .00902445627651
 0
 0
 0
 0
 46.7850374515
 0
 0
 0
 46.7850374515
 46.7850374515
 46.7850374515
 .41286887465
 .266221460157
 .266221460157
 .266221460157
 .146647414493
 .146647414493
 .146647414493
 .59335800018
 .59335800018
 .59335800018
 .59335800018
 .59335800018
 .139879072286
 .139879072286
 .139879072286
 .139879072286
 .139879072286
 .139879072286
 10.5044671058568
 3.87149174262
 3.87149174262
 3.87149174262
 3.87149174262
 3.87149174262
 6.16370363685865
 6.16370363685865
 6.13888638209825
 .00451222813825
 .00451222813825
 6.13437415396
 6.13437415396
 .0248172547604
 .0248172547604
 .0248172547604
 .46927172637815
 .45347892789425
 .448966699756
 .286526486779
 .286526486779
 .162440212977
 .162440212977
 .00451222813825
 .00451222813825
 0
 .00451222813825
 0
 0
 0
 0
 0
 0
 .0157927984839
 .0157927984839
 .0157927984839
 .0157927984839
 5.46882050356716
 5.04918328671
 5.04918328671
 5.04918328671
 5.04918328671
 5.04918328671
 .41963721685716
 .41963721685716
 .41512498871891
 .00902445627651
 .00902445627651
 .0609150798664
 .0609150798664
 .345185452576
 .345185452576
 .00451222813825
 .00451222813825
 .00451222813825
 0
 0
 1.67403663929
 1.67403663929
 1.67403663929
 1.67403663929
 1.67403663929
 1.67403663929
 0
 0
 0
 0
 0
 0
 0
 0
 .09024456276503
 .09024456276503
 .06993953614293
 .05414673765903
 .00225611406913
 .00225611406913
 .0518906235899
 .0518906235899
 .0157927984839
 .0157927984839
 .0157927984839
 0
 0
 0
 0
 .0203050266221
 .0203050266221
 .0203050266221
 .0203050266221
 .7738471257107
 .7738471257107
 .7738471257107
 .7738471257107
 .7738471257107
 .0473783954517
 .726468730259
 11.2241674939047
 11.2241674939047
 .0654273080047
 .0112805703456
 .0112805703456
 .0112805703456
 .0541467376591
 .0541467376591
 .0541467376591
 11.1587401859
 11.1587401859
 11.1587401859
 11.1587401859
 3.35258550672
 3.35258550672
 3.35258550672
 3.35258550672
 3.35258550672
 3.35258550672
 3.35258550672
