# Supplementary material for: Assessment of biomass potentials of microalgal communities in open pond raceways using mass cultivation
Source: PeerJ. 2020 Jul 16;8:e9418. doi: 10.7717/peerj.9418 (PMC7369025; doi:10.7717/peerj.9418)
Supplement: Data S5 [file peerj-08-9418-s022.zip › Krona/OPR#1/OPR#1_MAY.html]

Javascript must be enabled to view this page.

magnitude
 76.5269616877341
 64.0294009236341
 41.88330695342
 7.7578543396727
 .31872682725857
 .0520370330218
 .0520370330218
 .0520370330218
 .134429001973
 .0975694369159
 .0975694369159
 .0368595650571
 .0368595650571
 .071550920405
 .071550920405
 .071550920405
 0
 0
 0
 .06070987185877
 .00867283883697
 .00867283883697
 .0520370330218
 .0520370330218
 0
 0
 4.7288653758598
 .0130092582555
 .0130092582555
 .0130092582555
 4.7158561176043
 .0563734524403
 .0563734524403
 .188634244704
 .188634244704
 4.47084842046
 4.47084842046
 2.2007328548816
 1.3464582294396
 1.3464582294396
 .0650462912773
 .418464473884
 .637453654517
 .0737191301143
 .151774679647
 0
 0
 0
 .854274625442
 .854274625442
 .854274625442
 .20164350296
 .20164350296
 .20164350296
 .20164350296
 .30788577871273
 .30788577871273
 .301381149585
 .301381149585
 .00650462912773
 .00650462912773
 1.62182086251323
 1.49389648966823
 1.131805468225
 0
 0
 .936666594393
 .936666594393
 .195138873832
 .195138873832
 0
 0
 .36209102144323
 .271026213655
 .271026213655
 .0845601786605
 .0845601786605
 .00650462912773
 .00650462912773
 0
 0
 0
 0
 .127924372845
 .127924372845
 .127924372845
 .127924372845
 .0411959844756
 .0411959844756
 .0411959844756
 .0411959844756
 .0411959844756
 32.4450900890846
 .357754602025
 .357754602025
 .357754602025
 .357754602025
 .0195138873832
 .0195138873832
 .0195138873832
 .0195138873832
 31.9182151297386
 .0325231456386
 .0325231456386
 .0325231456386
 31.8856919841
 31.8856919841
 31.8856919841
 .1496064699378
 .0693827106958
 .0693827106958
 .0693827106958
 .080223759242
 .080223759242
 .080223759242
 .0173456776739
 .0173456776739
 .0173456776739
 .0173456776739
 .0173456776739
 .101905856334
 .101905856334
 .101905856334
 .101905856334
 .101905856334
 .101905856334
 12.6341579757593
 1.51557858676
 1.51557858676
 1.51557858676
 1.51557858676
 1.51557858676
 8.38229873593
 8.38229873593
 6.93393465016
 0
 0
 6.93393465016
 6.93393465016
 1.44836408577
 1.44836408577
 1.44836408577
 2.73628065306927
 2.44140413261224
 2.439235922903
 2.24843346849
 2.24843346849
 .190802454413
 .190802454413
 .00216820970924
 .00216820970924
 0
 .00216820970924
 .24717590685373
 .24717590685373
 .240671277726
 .240671277726
 .00650462912773
 .00650462912773
 .0477006136033
 .0477006136033
 .0477006136033
 .0477006136033
 2.99429760847319
 1.01689035364
 1.01689035364
 1.01689035364
 1.01689035364
 1.01689035364
 1.97740725483319
 1.97740725483319
 1.7931094295477
 .106242275753
 .106242275753
 .0151774679647
 .0151774679647
 1.67168968583
 1.67168968583
 .18429782528549
 .179961405867
 .179961405867
 .00433641941849
 .00433641941849
 .14743826022849
 .14310184081
 .14310184081
 .14310184081
 .14310184081
 .14310184081
 .00433641941849
 .00433641941849
 .00433641941849
 .00433641941849
 .00433641941849
 0
 0
 0
 .20381171266883
 .20381171266883
 .1322607922638
 .1105786951714
 .0346913553479
 .0346913553479
 .0758873398235
 .0758873398235
 .0216820970924
 .0216820970924
 .0216820970924
 .00650462912773
 .00650462912773
 .00650462912773
 .00650462912773
 .0650462912773
 .0650462912773
 .0650462912773
 .0650462912773
 2.1335183538964
 2.1335183538964
 2.1335183538964
 2.1335183538964
 2.1335183538964
 .0390277747664
 2.09449057913
 3.9309642028539
 3.9309642028539
 .7610416079439
 .717677413759
 .717677413759
 .717677413759
 .0433641941849
 .0433641941849
 .0433641941849
 3.16992259491
 3.16992259491
 3.16992259491
 3.16992259491
 12.4975607641
 12.4975607641
 12.4975607641
 12.4975607641
 12.4975607641
 12.4975607641
 12.4975607641
