# Supplementary material for: Assessment of biomass potentials of microalgal communities in open pond raceways using mass cultivation
Source: PeerJ. 2020 Jul 16;8:e9418. doi: 10.7717/peerj.9418 (PMC7369025; doi:10.7717/peerj.9418)
Supplement: Data S5 [file peerj-08-9418-s022.zip › Krona/OPR#1/OPR#1_NOV.html]

Javascript must be enabled to view this page.

magnitude
 75.7964774950751
 31.5225048923751
 16.9315068493218
 7.42074363992022
 .555772994129
 0
 0
 0
 .219178082192
 .219178082192
 .219178082192
 0
 0
 0
 0
 0
 0
 0
 0
 .336594911937
 .125244618395
 .125244618395
 .211350293542
 .211350293542
 0
 0
 1.07240704500761
 .00782778864971
 .00782778864971
 .00782778864971
 1.0645792563579
 1.00978473581
 1.00978473581
 0
 0
 .0547945205479
 .0547945205479
 1.35420743639761
 1.2602739726011
 1.2602739726011
 .0782778864971
 .156555772994
 1.02544031311
 0
 0
 .00782778864971
 .00782778864971
 .00782778864971
 .0861056751468
 .0861056751468
 .0861056751468
 1.4011741683
 1.4011741683
 1.4011741683
 1.4011741683
 3.037181996086
 3.037181996086
 .915851272016
 .915851272016
 2.12133072407
 2.12133072407
 3.2250489236831
 1.8943248532331
 1.4872798434491
 .0234833659491
 .0234833659491
 0
 0
 1.4637964775
 1.4637964775
 0
 0
 .407045009784
 0
 0
 .25831702544
 .25831702544
 .148727984344
 .148727984344
 0
 0
 0
 0
 1.33072407045
 1.33072407045
 1.33072407045
 1.33072407045
 0
 0
 0
 0
 0
 6.2152641878711
 .109589041096
 .109589041096
 .109589041096
 .109589041096
 0
 0
 0
 0
 5.4481409002
 0
 0
 0
 5.4481409002
 5.4481409002
 5.4481409002
 .6575342465751
 .634050880626
 .634050880626
 .634050880626
 .0234833659491
 .0234833659491
 .0234833659491
 .0704500978474
 .0704500978474
 .0704500978474
 .0704500978474
 .0704500978474
 .360078277886
 .360078277886
 .360078277886
 .360078277886
 .360078277886
 .360078277886
 2.86497064579421
 .0391389432485
 .0391389432485
 .0391389432485
 .0391389432485
 .0391389432485
 2.16829745597
 2.16829745597
 1.00978473581
 0
 0
 1.00978473581
 1.00978473581
 1.15851272016
 1.15851272016
 1.15851272016
 .65753424657571
 .156555772994
 .156555772994
 .156555772994
 .156555772994
 0
 0
 0
 0
 0
 0
 .00782778864971
 .00782778864971
 .00782778864971
 .00782778864971
 0
 0
 .493150684932
 .493150684932
 .493150684932
 .493150684932
 3.702544031306
 .892367906067
 .892367906067
 .892367906067
 .892367906067
 .892367906067
 2.810176125239
 2.810176125239
 .454011741682
 .125244618395
 .125244618395
 .148727984344
 .148727984344
 .180039138943
 .180039138943
 2.356164383557
 .884540117417
 .884540117417
 1.47162426614
 1.47162426614
 .688845401174
 .688845401174
 .688845401174
 .688845401174
 .688845401174
 .688845401174
 0
 0
 0
 0
 0
 0
 0
 0
 .37573385518582
 .37573385518582
 .30528375733841
 .2974559686887
 .0626223091977
 .0626223091977
 .234833659491
 .234833659491
 .00782778864971
 .00782778864971
 .00782778864971
 .0626223091977
 .0626223091977
 .0626223091977
 .0626223091977
 .00782778864971
 .00782778864971
 .00782778864971
 .00782778864971
 .0704500978473
 .0704500978473
 .0704500978473
 .0704500978473
 .0704500978473
 .0156555772994
 .0547945205479
 6.52837573386
 6.52837573386
 4.89236790607
 1.83953033268
 1.83953033268
 1.83953033268
 3.05283757339
 3.05283757339
 3.05283757339
 1.63600782779
 1.63600782779
 1.63600782779
 1.63600782779
 44.2739726027
 44.2739726027
 44.2739726027
 44.2739726027
 44.2739726027
 44.2739726027
 44.2739726027
