# Supplementary material for: Assessment of biomass potentials of microalgal communities in open pond raceways using mass cultivation
Source: PeerJ. 2020 Jul 16;8:e9418. doi: 10.7717/peerj.9418 (PMC7369025; doi:10.7717/peerj.9418)
Supplement: Data S5 [file peerj-08-9418-s022.zip › Krona/OPR#1/OPR#1_OCT.html]

Javascript must be enabled to view this page.

magnitude
 46.7661276879208
 23.8706451075208
 12.5604267377934
 4.000666777799
 .6834472412072
 0
 0
 0
 .175029171529
 .175029171529
 .175029171529
 0
 0
 0
 0
 0
 0
 0
 0
 .5084180696782
 .33338889815
 .33338889815
 .150025004167
 .150025004167
 .0250041673612
 .0250041673612
 1.3002167027887
 .0416736122687
 .0416736122687
 .0416736122687
 1.25854309052
 1.25854309052
 1.25854309052
 0
 0
 0
 0
 .7001166861141
 .6834472412066
 .6834472412066
 .0833472245374
 .0250041673612
 .575095849308
 0
 0
 0
 0
 0
 .0166694449075
 .0166694449075
 .0166694449075
 1.14185697616
 1.14185697616
 1.14185697616
 1.14185697616
 .175029171529
 .175029171529
 .141690281714
 .141690281714
 .033338889815
 .033338889815
 2.9504917486257
 .9918319719957
 .883480580097
 0
 0
 0
 0
 .883480580097
 .883480580097
 0
 0
 .1083513918987
 0
 0
 .0166694449075
 .0166694449075
 .0916819469912
 .0916819469912
 0
 0
 0
 0
 1.95865977663
 1.95865977663
 1.95865977663
 1.95865977663
 0
 0
 0
 0
 0
 5.600933488915
 .116686114352
 .116686114352
 .116686114352
 .116686114352
 0
 0
 0
 0
 4.74245707618
 0
 0
 0
 4.74245707618
 4.74245707618
 4.74245707618
 .741790298383
 .741790298383
 .741790298383
 .741790298383
 0
 0
 0
 .00833472245374
 .00833472245374
 .00833472245374
 .00833472245374
 .00833472245374
 .833472245374
 .833472245374
 .833472245374
 .833472245374
 .833472245374
 .833472245374
 6.6177696282707
 .716786131022
 .716786131022
 .716786131022
 .716786131022
 .716786131022
 4.76746124354
 4.76746124354
 2.80880146691
 0
 0
 2.80880146691
 2.80880146691
 1.95865977663
 1.95865977663
 1.95865977663
 1.1335222537087
 .808468078013
 .808468078013
 .808468078013
 .808468078013
 0
 0
 0
 0
 0
 0
 .0416736122687
 .0416736122687
 .0416736122687
 .0416736122687
 0
 0
 .283380563427
 .283380563427
 .283380563427
 .283380563427
 1.72528754792424
 .158359726621
 .158359726621
 .158359726621
 .158359726621
 .158359726621
 1.56692782130324
 1.56692782130324
 .82513752292024
 .00833472245374
 .00833472245374
 .766794465744
 .766794465744
 .0500083347225
 .0500083347225
 .741790298383
 .425070845141
 .425070845141
 .316719453242
 .316719453242
 .125020836806
 .125020836806
 .125020836806
 .125020836806
 .125020836806
 .125020836806
 0
 0
 0
 0
 0
 0
 0
 0
 .8501416902817
 .8501416902817
 .7917986331055
 .775129188198
 .116686114352
 .116686114352
 .658443073846
 .658443073846
 .0166694449075
 .0166694449075
 .0166694449075
 .0583430571762
 .0583430571762
 .0583430571762
 .0583430571762
 0
 0
 0
 0
 .0416736122687
 .0416736122687
 .0416736122687
 .0416736122687
 .0416736122687
 .0166694449075
 .0250041673612
 1.116852808802
 1.116852808802
 .766794465745
 .233372228705
 .233372228705
 .233372228705
 .53342223704
 .53342223704
 .53342223704
 .350058343057
 .350058343057
 .350058343057
 .350058343057
 22.8954825804
 22.8954825804
 22.8954825804
 22.8954825804
 22.8954825804
 22.8954825804
 22.8954825804
