# Supplementary material for: Assessment of biomass potentials of microalgal communities in open pond raceways using mass cultivation
Source: PeerJ. 2020 Jul 16;8:e9418. doi: 10.7717/peerj.9418 (PMC7369025; doi:10.7717/peerj.9418)
Supplement: Data S5 [file peerj-08-9418-s022.zip › Krona/OPR#1/OPR#1_SEP.html]

Javascript must be enabled to view this page.

magnitude
 65.59139784946
 32.9387392719599
 6.19512676334744
 2.84107724178774
 .2268915852819
 0
 0
 0
 .138107921476
 .138107921476
 .138107921476
 0
 0
 0
 0
 0
 0
 0
 0
 .0887836638059
 0
 0
 .0591891092039
 .0591891092039
 .029594554602
 .029594554602
 .927296044195
 0
 0
 0
 .927296044195
 .927296044195
 .927296044195
 0
 0
 0
 0
 1.15418762947588
 1.13445792640788
 1.13445792640788
 .0591891092039
 .00986485153398
 1.06540396567
 0
 0
 0
 0
 0
 .019729703068
 .019729703068
 .019729703068
 .512972279767
 .512972279767
 .512972279767
 .512972279767
 .01972970306796
 .01972970306796
 .00986485153398
 .00986485153398
 .00986485153398
 .00986485153398
 2.9002663509959
 .0887836638059
 .029594554602
 0
 0
 0
 0
 .029594554602
 .029594554602
 0
 0
 .0591891092039
 0
 0
 0
 0
 .0591891092039
 .0591891092039
 0
 0
 0
 0
 2.81148268719
 2.81148268719
 2.81148268719
 2.81148268719
 0
 0
 0
 0
 0
 .3649995067579
 .0394594061359
 .0394594061359
 .0394594061359
 .0394594061359
 0
 0
 0
 0
 .128243069942
 0
 0
 0
 .128243069942
 .128243069942
 .128243069942
 .19729703068
 .19729703068
 .19729703068
 .19729703068
 0
 0
 0
 .0887836638059
 .0887836638059
 .0887836638059
 .0887836638059
 .0887836638059
 .305810397554
 .305810397554
 .305810397554
 .305810397554
 .305810397554
 .305810397554
 16.0303837427039
 .226891585282
 .226891585282
 .226891585282
 .226891585282
 .226891585282
 15.093222846975
 15.093222846975
 .631350498175
 0
 0
 .631350498175
 .631350498175
 14.4618723488
 14.4618723488
 14.4618723488
 .7102693104469
 .631350498175
 .631350498175
 .631350498175
 .631350498175
 0
 0
 0
 0
 0
 0
 0
 0
 0
 0
 0
 0
 .0789188122719
 .0789188122719
 .0789188122719
 .0789188122719
 1.874321791457
 .394594061359
 .394594061359
 .394594061359
 .394594061359
 .394594061359
 1.479727730098
 1.479727730098
 .947025747263
 .029594554602
 .029594554602
 .917431192661
 .917431192661
 0
 0
 .532701982835
 .029594554602
 .029594554602
 .503107428233
 .503107428233
 .256486139884
 .256486139884
 .256486139884
 .256486139884
 .256486139884
 .256486139884
 0
 0
 0
 0
 0
 0
 0
 0
 .2959455460198
 .2959455460198
 .2367564368159
 .2367564368159
 .0394594061359
 .0394594061359
 .19729703068
 .19729703068
 0
 0
 0
 .0591891092039
 .0591891092039
 .0591891092039
 .0591891092039
 0
 0
 0
 0
 .0887836638058
 .0887836638058
 .0887836638058
 .0887836638058
 .0887836638058
 .0493242576699
 .0394594061359
 7.891881227188
 7.891881227188
 4.478642596428
 4.36026437802
 4.36026437802
 4.36026437802
 .118378218408
 .118378218408
 .118378218408
 3.41323863076
 3.41323863076
 3.41323863076
 3.41323863076
 32.6526585775
 32.6526585775
 32.6526585775
 32.6526585775
 32.6526585775
 32.6526585775
 32.6526585775
