# Supplementary material for: Assessment of biomass potentials of microalgal communities in open pond raceways using mass cultivation
Source: PeerJ. 2020 Jul 16;8:e9418. doi: 10.7717/peerj.9418 (PMC7369025; doi:10.7717/peerj.9418)
Supplement: Data S5 [file peerj-08-9418-s022.zip › Krona/OPR#1.html]

Javascript must be enabled to view this page.

magnitude
 76.6687332750982
 57.453194183272
 29.3683362649635
 6.60164397129437
 .543532366425977
 8.28853837215625E-02
 8.28853837215625E-02
 8.28853837215625E-02
 .148591310322731
 .133870326586838
 .133870326586838
 1.47209837358938E-02
 1.47209837358938E-02
 5.14773500130125E-02
 5.14773500130125E-02
 5.14773500130125E-02
 .032653161811875
 .032653161811875
 .032653161811875
 .227925160556796
 7.68962162029962E-02
 7.68962162029962E-02
 .138351178877975
 .138351178877975
 .012677765475825
 .012677765475825
 2.545722048702
 1.71550664816388E-02
 1.71550664816388E-02
 1.71550664816388E-02
 2.52856698222036
 .444723389038575
 .444723389038575
 6.89920245334262E-02
 6.89920245334262E-02
 2.01485156864836
 2.01485156864836
 1.76984422454648
 1.37278130749473
 1.37278130749473
 .113302812003025
 .197816267935348
 .981136697368875
 3.17540812661012E-02
 4.87714489213813E-02
 6.10479693670888E-02
 6.10479693670888E-02
 6.10479693670888E-02
 .336014947684663
 .336014947684663
 .336014947684663
 1.23427911617363
 1.23427911617363
 1.23427911617363
 1.23427911617363
 .50826621544628
 .50826621544628
 .230469374880235
 .230469374880235
 .277796840566045
 .277796840566045
 3.40456051672374
 2.21184289729937
 1.74571532180335
 .264776812631138
 .264776812631138
 .474089647054175
 .474089647054175
 .956020827222288
 .956020827222288
 .05082803489575
 .05082803489575
 .466127575496019
 .318662114731625
 .318662114731625
 .07035516629585
 .07035516629585
 7.71102944685437E-02
 7.71102944685437E-02
 .0351175513825
 .0351175513825
 .0351175513825
 .0351175513825
 1.15760006804188
 1.15760006804188
 1.15760006804188
 1.15760006804188
 3.28478462922163E-02
 3.28478462922163E-02
 3.28478462922163E-02
 3.28478462922163E-02
 3.28478462922163E-02
 19.0647949329982
 .173944821187714
 .173944821187714
 .173944821187714
 .173944821187714
 9.8525260620675E-03
 9.8525260620675E-03
 9.8525260620675E-03
 9.8525260620675E-03
 18.4294526178783
 .068139522043075
 .068139522043075
 .068139522043075
 18.3613130958352
 18.3613130958352
 18.3613130958352
 .451544967870113
 .352075941550225
 .352075941550225
 .352075941550225
 9.94690263198875E-02
 9.94690263198875E-02
 9.94690263198875E-02
 .264488997654993
 .264488997654993
 .264488997654993
 .264488997654993
 .264488997654993
 .28718662674925
 .28718662674925
 .28718662674925
 .28718662674925
 .28718662674925
 .28718662674925
 15.3368060991326
 2.84167975149369
 2.84167975149369
 2.84167975149369
 2.84167975149369
 2.84167975149369
 9.25517549970633
 9.25517549970633
 4.98171017562716
 1.99710963865313E-02
 1.99710963865313E-02
 4.96173907924063
 4.96173907924063
 4.27346532407917
 4.27346532407917
 4.27346532407917
 3.23995084793262
 2.2146250263235
 2.03875794779101
 .57881041422025
 .57881041422025
 1.45994753357076
 1.45994753357076
 .175867078532487
 .175867078532487
 2.34720652028012E-02
 .152395013329686
 .158455849828643
 .158455849828643
 .149325456386551
 .149325456386551
 9.13039344209125E-03
 9.13039344209125E-03
 .866869971780475
 .866869971780475
 .866869971780475
 .866869971780475
 3.60014685404935
 1.7620281774885
 1.7620281774885
 1.7620281774885
 1.7620281774885
 1.7620281774885
 1.83811867656085
 1.83811867656085
 1.13220832035366
 .113495248315206
 .113495248315206
 .437065603550512
 .437065603550512
 .581647468487937
 .581647468487937
 .705910356207198
 .368280792044549
 .368280792044549
 .337629564162649
 .337629564162649
 .604708934191424
 .485807838367288
 .485807838367288
 .485807838367288
 .485807838367288
 .485807838367288
 .118901095824136
 .118901095824136
 .112124024504699
 .112124024504699
 .112124024504699
 6.7770713194375E-03
 6.7770713194375E-03
 6.7770713194375E-03
 .415820049446597
 .415820049446597
 .280191638076442
 .249724110865604
 4.91138489471787E-02
 4.91138489471787E-02
 .200610261918425
 .200610261918425
 3.04675272108388E-02
 3.04675272108388E-02
 3.04675272108388E-02
 5.89549598512288E-02
 5.89549598512288E-02
 5.89549598512288E-02
 5.89549598512288E-02
 7.66734515189263E-02
 7.66734515189263E-02
 7.66734515189263E-02
 7.66734515189263E-02
 .829984449666537
 .829984449666537
 .829984449666537
 .829984449666537
 .829984449666537
 .263185990871537
 .566798458795
 7.01020490507266
 7.01020490507266
 1.49063735752179
 .998932807468037
 .998932807468037
 .998932807468037
 .49170455005375
 .49170455005375
 .49170455005375
 5.51956754755087
 5.51956754755087
 5.51956754755087
 5.51956754755087
 19.2155390918263
 19.2155390918263
 19.2155390918263
 19.2155390918263
 19.2155390918263
 19.2155390918263
 19.2155390918263
