# Supplementary material for: Assessment of biomass potentials of microalgal communities in open pond raceways using mass cultivation
Source: PeerJ. 2020 Jul 16;8:e9418. doi: 10.7717/peerj.9418 (PMC7369025; doi:10.7717/peerj.9418)
Supplement: Data S5 [file peerj-08-9418-s022.zip › Krona/OPR#3/OPR#3_APR.html]

Javascript must be enabled to view this page.

magnitude
 46.6242826600805
 42.2977382694005
 21.911218633966
 8.76561269270038
 .83267694385088
 .230674018229
 .230674018229
 .230674018229
 .15753347586328
 .15472037808
 .15472037808
 .00281309778328
 .00281309778328
 .219421627096
 .219421627096
 .219421627096
 .0365702711826
 .0365702711826
 .0365702711826
 .18847755148
 .019691684483
 .019691684483
 .168785866997
 .168785866997
 0
 0
 6.27039495893284
 .00562619556656
 .00562619556656
 .00562619556656
 6.26476876336628
 .371328907393
 .371328907393
 .00281309778328
 .00281309778328
 5.89062675819
 5.89062675819
 .91144368178166
 .7904804771011
 .7904804771011
 .0787667379318
 .112523911331
 .51479689434
 .0478226623157
 .0365702711826
 .00562619556656
 .00562619556656
 .00562619556656
 .115337009114
 .115337009114
 .115337009114
 .751097108135
 .751097108135
 .751097108135
 .751097108135
 0
 0
 0
 0
 0
 0
 4.8863508495552
 4.3377967818162
 4.2196466749184
 .00843929334984
 .00843929334984
 3.79205581186
 3.79205581186
 .413525374142
 .413525374142
 .00562619556656
 .00562619556656
 .1181501068978
 .0450095645325
 .0450095645325
 .0590750534489
 .0590750534489
 .0140654889164
 .0140654889164
 0
 0
 0
 0
 .548554067739
 .548554067739
 .548554067739
 .548554067739
 .0253178800495
 .0253178800495
 .0253178800495
 .0253178800495
 .0253178800495
 8.23393721166088
 .0534488578823
 .0534488578823
 .0534488578823
 .0534488578823
 .00281309778328
 .00281309778328
 .00281309778328
 .00281309778328
 7.9638798244656
 .0562619556656
 .0562619556656
 .0562619556656
 7.9076178688
 7.9076178688
 7.9076178688
 .2137954315297
 .19691684483
 .19691684483
 .19691684483
 .0168785866997
 .0168785866997
 .0168785866997
 0
 0
 0
 0
 0
 .261618093845
 .261618093845
 .261618093845
 .261618093845
 .261618093845
 .261618093845
 1.82570046134724
 .019691684483
 .019691684483
 .019691684483
 .019691684483
 .019691684483
 .18847755147946
 .18847755147946
 .1828513559129
 .123776302464
 .123776302464
 .0590750534489
 .0590750534489
 .00562619556656
 .00562619556656
 .00562619556656
 1.61753122538478
 1.41780128277178
 .2728704849785
 .247552604929
 .247552604929
 .0253178800495
 .0253178800495
 1.14493079779328
 1.14493079779328
 .00281309778328
 1.14211770001
 0
 0
 0
 0
 0
 0
 .199729942613
 .199729942613
 .199729942613
 .199729942613
 7.5109710813591
 .219421627096
 .219421627096
 .219421627096
 .219421627096
 .219421627096
 7.2915494542631
 7.2915494542631
 7.229661303031
 1.0436592776
 1.0436592776
 .120963204681
 .120963204681
 6.06503882075
 6.06503882075
 .0618881512321
 .0393833689659
 .0393833689659
 .0225047822662
 .0225047822662
 1.41217508721
 1.41217508721
 1.41217508721
 1.41217508721
 1.41217508721
 1.41217508721
 0
 0
 0
 0
 0
 0
 0
 0
 .53730167660584
 .53730167660584
 .08720603128164
 .05344885788234
 .00843929334984
 .00843929334984
 .0450095645325
 .0450095645325
 .0337571733993
 .0337571733993
 .0337571733993
 .0421964667492
 .0421964667492
 .0421964667492
 .0421964667492
 .407899178575
 .407899178575
 .407899178575
 .407899178575
 .1941037470464
 .1941037470464
 .1941037470464
 .1941037470464
 .1941037470464
 .0140654889164
 .18003825813
 8.644649488021
 8.644649488021
 8.52368628334
 6.07066501632
 6.07066501632
 6.07066501632
 2.45302126702
 2.45302126702
 2.45302126702
 .120963204681
 .120963204681
 .120963204681
 .120963204681
 4.32654439068
 4.32654439068
 4.32654439068
 4.32654439068
 4.32654439068
 4.32654439068
 4.32654439068
