# Supplementary material for: Assessment of biomass potentials of microalgal communities in open pond raceways using mass cultivation
Source: PeerJ. 2020 Jul 16;8:e9418. doi: 10.7717/peerj.9418 (PMC7369025; doi:10.7717/peerj.9418)
Supplement: Data S5 [file peerj-08-9418-s022.zip › Krona/OPR#3/OPR#3_AUG.html]

Javascript must be enabled to view this page.

magnitude
 83.9273308322392
 56.4401834097391
 10.0875364735303
 1.1810476587468
 .13547311379735
 .00347366958455
 .00347366958455
 .00347366958455
 .0347366958455
 .0347366958455
 .0347366958455
 0
 0
 .0590523829373
 .0590523829373
 .0590523829373
 0
 0
 0
 .03821036543
 0
 0
 .0277893566764
 .0277893566764
 .0104210087536
 .0104210087536
 .1528414617202
 0
 0
 0
 .1528414617202
 .0555787133528
 .0555787133528
 0
 0
 .0972627483674
 .0972627483674
 .64610254272625
 .49673475059025
 .49673475059025
 .125052105044
 .0104210087536
 .350840628039
 .0069473391691
 .00347366958455
 0
 0
 0
 .149367792136
 .149367792136
 .149367792136
 .246630540503
 .246630540503
 .246630540503
 .246630540503
 0
 0
 0
 0
 0
 0
 3.8557732388475
 .4863137418375
 .1841044879815
 0
 0
 .111157426706
 .111157426706
 .0729470612755
 .0729470612755
 0
 0
 .302209253856
 .302209253856
 .302209253856
 0
 0
 0
 0
 0
 0
 0
 0
 3.36945949701
 3.36945949701
 3.36945949701
 3.36945949701
 0
 0
 0
 0
 0
 4.147561483953
 .166736140058
 .166736140058
 .166736140058
 .166736140058
 0
 0
 0
 0
 3.48061692372
 0
 0
 0
 3.48061692372
 3.48061692372
 3.48061692372
 .500208420175
 .399472002223
 .399472002223
 .399472002223
 .100736417952
 .100736417952
 .100736417952
 .903154091983
 .903154091983
 .903154091983
 .903154091983
 .903154091983
 .0416840350146
 .0416840350146
 .0416840350146
 .0416840350146
 .0416840350146
 .0416840350146
 21.297068222902
 .639155203557
 .639155203557
 .639155203557
 .639155203557
 .639155203557
 7.15923301376
 7.15923301376
 5.40502987356
 0
 0
 5.40502987356
 5.40502987356
 1.7542031402
 1.7542031402
 1.7542031402
 13.498680005585
 .385577323885
 .385577323885
 .121578435459
 .121578435459
 .263998888426
 .263998888426
 0
 0
 0
 0
 0
 0
 0
 0
 0
 0
 13.1131026817
 13.1131026817
 13.1131026817
 13.1131026817
 12.8942614977999
 12.4670001389
 12.4670001389
 12.4670001389
 12.4670001389
 12.4670001389
 .4272613588999
 .4272613588999
 .2674725580108
 .118104765875
 .118104765875
 .0243156870918
 .0243156870918
 .125052105044
 .125052105044
 .1597888008891
 .0451577045991
 .0451577045991
 .11463109629
 .11463109629
 1.19494233708555
 .218841183827
 .218841183827
 .218841183827
 .218841183827
 .218841183827
 .97610115325855
 .97610115325855
 .972627483674
 .972627483674
 .972627483674
 .00347366958455
 .00347366958455
 .00347366958455
 .1007364179518
 .1007364179518
 .0555787133527
 .0416840350145
 .0243156870918
 .0243156870918
 .0173683479227
 .0173683479227
 .0138946783382
 .0138946783382
 .0138946783382
 .0451577045991
 .0451577045991
 .0451577045991
 .0451577045991
 0
 0
 0
 0
 1.347783798805
 1.347783798805
 1.347783798805
 1.347783798805
 1.347783798805
 .88578574406
 .461998054745
 9.47617062665
 9.47617062665
 .03821036543
 .03821036543
 .03821036543
 .03821036543
 0
 0
 0
 9.43796026122
 9.43796026122
 9.43796026122
 9.43796026122
 27.4871474225
 27.4871474225
 27.4871474225
 27.4871474225
 27.4871474225
 27.4871474225
 27.4871474225
