# Supplementary material for: Assessment of biomass potentials of microalgal communities in open pond raceways using mass cultivation
Source: PeerJ. 2020 Jul 16;8:e9418. doi: 10.7717/peerj.9418 (PMC7369025; doi:10.7717/peerj.9418)
Supplement: Data S5 [file peerj-08-9418-s022.zip › Krona/OPR#3/OPR#3_JUL.html]

Javascript must be enabled to view this page.

magnitude
 72.2216364403128
 55.9784900885128
 23.9798608181932
 9.23397300717338
 .3110501897934
 .0948966680725
 .0948966680725
 .0948966680725
 .147617039224
 .147617039224
 .147617039224
 0
 0
 .0395402783636
 .0395402783636
 .0395402783636
 0
 0
 0
 .0289962041333
 0
 0
 .0289962041333
 .0289962041333
 0
 0
 .45075917334458
 .00263601855757
 .00263601855757
 .00263601855757
 .44812315478701
 .0843525938423
 .0843525938423
 .00790805567271
 .00790805567271
 .355862505272
 .355862505272
 1.8399409531854
 1.5684310417554
 1.5684310417554
 .632644453817
 .168705187685
 .732813159005
 0
 .0342682412484
 0
 0
 0
 .27150991143
 .27150991143
 .27150991143
 6.63222269085
 6.63222269085
 6.63222269085
 6.63222269085
 0
 0
 0
 0
 0
 0
 3.02614930409614
 1.47089835512614
 1.294285111769
 0
 0
 1.1308519612
 1.1308519612
 .163433150569
 .163433150569
 0
 0
 .17661324335714
 .171341206242
 .171341206242
 .00263601855757
 .00263601855757
 .00263601855757
 .00263601855757
 0
 0
 0
 0
 1.55525094897
 1.55525094897
 1.55525094897
 1.55525094897
 0
 0
 0
 0
 0
 10.9790172922467
 .440215099114
 .440215099114
 .440215099114
 .440215099114
 .00263601855757
 .00263601855757
 .00263601855757
 .00263601855757
 10.2488401518
 0
 0
 0
 10.2488401518
 10.2488401518
 10.2488401518
 .2873260227751
 .245149725854
 .245149725854
 .245149725854
 .0421762969211
 .0421762969211
 .0421762969211
 .740721214677
 .740721214677
 .740721214677
 .740721214677
 .740721214677
 .0579924082666
 .0579924082666
 .0579924082666
 .0579924082666
 .0579924082666
 .0579924082666
 21.0248840151447
 10.1302193167
 10.1302193167
 10.1302193167
 10.1302193167
 10.1302193167
 10.240932096167
 10.240932096167
 9.71636440321
 0
 0
 9.71636440321
 9.71636440321
 .524567692957
 .524567692957
 .524567692957
 .65373260227767
 .1528890763391
 .1528890763391
 .0948966680725
 .0948966680725
 .0579924082666
 .0579924082666
 0
 0
 0
 0
 .00263601855757
 .00263601855757
 .00263601855757
 .00263601855757
 0
 0
 .498207507381
 .498207507381
 .498207507381
 .498207507381
 4.63148460565142
 2.90489245044
 2.90489245044
 2.90489245044
 2.90489245044
 2.90489245044
 1.72659215521142
 1.72659215521142
 1.710776043866
 .092260649515
 .092260649515
 .487663433151
 .487663433151
 1.1308519612
 1.1308519612
 .01581611134542
 .00790805567271
 .00790805567271
 .00790805567271
 .00790805567271
 .80134964150157
 .630008435259
 .630008435259
 .630008435259
 .630008435259
 .630008435259
 .17134120624257
 .17134120624257
 .168705187685
 .168705187685
 .168705187685
 .00263601855757
 .00263601855757
 .00263601855757
 .2161535217207
 .2161535217207
 .0869886123998
 .0500843525938
 .018452129903
 .018452129903
 .0316322226908
 .0316322226908
 .036904259806
 .036904259806
 .036904259806
 .115984816533
 .115984816533
 .115984816533
 .115984816533
 .0131800927879
 .0131800927879
 .0131800927879
 .0131800927879
 3.21594264024
 3.21594264024
 3.21594264024
 3.21594264024
 3.21594264024
 1.45508224378
 1.76086039646
 2.0508224377947
 2.0508224377947
 .0817165752847
 .0606284268241
 .0606284268241
 .0606284268241
 .0210881484606
 .0210881484606
 .0210881484606
 1.96910586251
 1.96910586251
 1.96910586251
 1.96910586251
 16.2431463518
 16.2431463518
 16.2431463518
 16.2431463518
 16.2431463518
 16.2431463518
 16.2431463518
