# Supplementary material for: Assessment of biomass potentials of microalgal communities in open pond raceways using mass cultivation
Source: PeerJ. 2020 Jul 16;8:e9418. doi: 10.7717/peerj.9418 (PMC7369025; doi:10.7717/peerj.9418)
Supplement: Data S5 [file peerj-08-9418-s022.zip › Krona/OPR#3/OPR#3_JUN.html]

Javascript must be enabled to view this page.

magnitude
 86.1435225752691
 69.7551076880691
 31.0058571230759
 5.94936125074292
 .36434072775902
 .0184476317853
 .0184476317853
 .0184476317853
 .16833464004032
 .163722732094
 .163722732094
 .00461190794632
 .00461190794632
 .142969146336
 .142969146336
 .142969146336
 0
 0
 0
 .0345893095974
 0
 0
 .0345893095974
 .0345893095974
 0
 0
 1.5888022875028
 .013835723839
 .013835723839
 .013835723839
 1.5749665636638
 .0530369413827
 .0530369413827
 .0299774016511
 .0299774016511
 1.49195222063
 1.49195222063
 2.2275515380711
 1.8170917308491
 1.8170917308491
 .650279020431
 .426601485034
 .551122999585
 .0299774016511
 .159110824148
 0
 0
 0
 .410459807222
 .410459807222
 .410459807222
 1.76866669741
 1.76866669741
 1.76866669741
 1.76866669741
 0
 0
 0
 0
 0
 0
 3.76792879214016
 3.51196790111916
 3.02310565881
 0
 0
 2.36590877646
 2.36590877646
 .65719688235
 .65719688235
 0
 0
 .48886224230916
 .486556288336
 .486556288336
 .00230595397316
 .00230595397316
 0
 0
 0
 0
 0
 0
 .255960891021
 .255960891021
 .255960891021
 .255960891021
 .00230595397316
 .00230595397316
 .00230595397316
 .00230595397316
 .00230595397316
 21.1525157957767
 .0484250334363
 .0484250334363
 .0484250334363
 .0484250334363
 0
 0
 0
 0
 20.9565097080584
 .0207535857584
 .0207535857584
 .0207535857584
 20.9357561223
 20.9357561223
 20.9357561223
 .147581054282
 .103767928792
 .103767928792
 .103767928792
 .04381312549
 .04381312549
 .04381312549
 .133745330443
 .133745330443
 .133745330443
 .133745330443
 .133745330443
 .08762625098
 .08762625098
 .08762625098
 .08762625098
 .08762625098
 .08762625098
 24.5607157681494
 7.41133606973
 7.41133606973
 7.41133606973
 7.41133606973
 7.41133606973
 16.7112484435195
 16.7112484435195
 16.7112484435195
 .00691786191948
 .00691786191948
 16.7043305816
 16.7043305816
 0
 0
 0
 .4381312548999
 .2905502006179
 .2905502006179
 .26287875294
 .26287875294
 .0276714476779
 .0276714476779
 0
 0
 0
 0
 0
 0
 0
 0
 0
 0
 .147581054282
 .147581054282
 .147581054282
 .147581054282
 2.05691094405348
 .465802702578
 .465802702578
 .465802702578
 .465802702578
 .465802702578
 1.59110824147548
 1.59110824147548
 1.584190379556
 .17525250196
 .17525250196
 .136051284416
 .136051284416
 1.27288659318
 1.27288659318
 .00691786191948
 .00691786191948
 .00691786191948
 0
 0
 2.0430752202153
 2.02462758843
 2.02462758843
 2.02462758843
 2.02462758843
 2.02462758843
 .0184476317853
 .0184476317853
 .0184476317853
 .0184476317853
 .0184476317853
 0
 0
 0
 .2974680625374
 .2974680625374
 .1775584559332
 .1591108241479
 .0645667112484
 .0645667112484
 .0945441128995
 .0945441128995
 .0184476317853
 .0184476317853
 .0184476317853
 .0322833556242
 .0322833556242
 .0322833556242
 .0322833556242
 .08762625098
 .08762625098
 .08762625098
 .08762625098
 3.149933127338
 3.149933127338
 3.149933127338
 3.149933127338
 3.149933127338
 .647973066458
 2.50196006088
 6.5535211917196
 6.5535211917196
 .1821703638796
 .115297698658
 .115297698658
 .115297698658
 .0668726652216
 .0668726652216
 .0668726652216
 6.37135082784
 6.37135082784
 6.37135082784
 6.37135082784
 16.3884148872
 16.3884148872
 16.3884148872
 16.3884148872
 16.3884148872
 16.3884148872
 16.3884148872
