# Supplementary material for: Assessment of biomass potentials of microalgal communities in open pond raceways using mass cultivation
Source: PeerJ. 2020 Jul 16;8:e9418. doi: 10.7717/peerj.9418 (PMC7369025; doi:10.7717/peerj.9418)
Supplement: Data S5 [file peerj-08-9418-s022.zip › Krona/OPR#3/OPR#3_MAY.html]

Javascript must be enabled to view this page.

magnitude
 87.7591973244475
 62.7793795410475
 39.7232153154654
 4.66843501325897
 .5166647445503
 .0438242417253
 .0438242417253
 .0438242417253
 .170683888825
 .170683888825
 .170683888825
 0
 0
 .18913620113
 .18913620113
 .18913620113
 0
 0
 0
 .11302041287
 0
 0
 .11302041287
 .11302041287
 0
 0
 1.98593011186447
 .00230653903817
 .00230653903817
 .00230653903817
 1.9836235728263
 .110713873832
 .110713873832
 .0576634759543
 .0576634759543
 1.81524622304
 1.81524622304
 1.7022258101712
 1.2755160881092
 1.2755160881092
 .177603505939
 .348287394764
 .359820089955
 .0553569369162
 .334448160535
 0
 0
 0
 .426709722062
 .426709722062
 .426709722062
 .463614346673
 .463614346673
 .463614346673
 .463614346673
 0
 0
 0
 0
 0
 0
 1.00103794256697
 .80959520239897
 .756544804521
 0
 0
 .574328220505
 .574328220505
 .182216584016
 .182216584016
 0
 0
 .05305039787797
 .0507438588398
 .0507438588398
 .00230653903817
 .00230653903817
 0
 0
 0
 0
 0
 0
 .191442740168
 .191442740168
 .191442740168
 .191442740168
 .00230653903817
 .00230653903817
 .00230653903817
 .00230653903817
 .00230653903817
 34.0445162034868
 .00230653903817
 .00230653903817
 .00230653903817
 .00230653903817
 .0299850074963
 .0299850074963
 .0299850074963
 .0299850074963
 33.9130434783108
 .0369046246108
 .0369046246108
 .0369046246108
 33.8761388537
 33.8761388537
 33.8761388537
 .09918117864149
 .0899550224888
 .0899550224888
 .0899550224888
 .00922615615269
 .00922615615269
 .00922615615269
 .00691961711452
 .00691961711452
 .00691961711452
 .00691961711452
 .00691961711452
 .0738092492215
 .0738092492215
 .0738092492215
 .0738092492215
 .0738092492215
 .0738092492215
 7.5931265136719
 .0299850074963
 .0299850074963
 .0299850074963
 .0299850074963
 .0299850074963
 6.599008188219
 6.599008188219
 6.599008188219
 .140698881329
 .140698881329
 6.45830930689
 6.45830930689
 0
 0
 0
 .9641333179566
 .8418867489336
 .8418867489336
 .82112789759
 .82112789759
 .0207588513436
 .0207588513436
 0
 0
 0
 0
 0
 0
 0
 0
 0
 0
 .122246569023
 .122246569023
 .122246569023
 .122246569023
 2.45646407565517
 1.13712374582
 1.13712374582
 1.13712374582
 1.13712374582
 1.13712374582
 1.31934032983517
 1.31934032983517
 1.317033790797
 .495905893207
 .495905893207
 .1268596471
 .1268596471
 .69426825049
 .69426825049
 .00230653903817
 0
 0
 .00230653903817
 .00230653903817
 .08764848345057
 .0853419444124
 .0853419444124
 .0853419444124
 .0853419444124
 .0853419444124
 .00230653903817
 .00230653903817
 0
 0
 0
 .00230653903817
 .00230653903817
 .00230653903817
 .6066197670395
 .6066197670395
 .2306539038173
 .200668896321
 .066889632107
 .066889632107
 .133779264214
 .133779264214
 .0299850074963
 .0299850074963
 .0299850074963
 .0691961711452
 .0691961711452
 .0691961711452
 .0691961711452
 .306769692077
 .306769692077
 .306769692077
 .306769692077
 1.5084765309615
 1.5084765309615
 1.5084765309615
 1.5084765309615
 1.5084765309615
 .0738092492215
 1.43466728174
 10.730019605582
 10.730019605582
 2.006688963212
 1.7944873717
 1.7944873717
 1.7944873717
 .212201591512
 .212201591512
 .212201591512
 8.72333064237
 8.72333064237
 8.72333064237
 8.72333064237
 24.9798177834
 24.9798177834
 24.9798177834
 24.9798177834
 24.9798177834
 24.9798177834
 24.9798177834
