# Supplementary material for: Assessment of biomass potentials of microalgal communities in open pond raceways using mass cultivation
Source: PeerJ. 2020 Jul 16;8:e9418. doi: 10.7717/peerj.9418 (PMC7369025; doi:10.7717/peerj.9418)
Supplement: Data S5 [file peerj-08-9418-s022.zip › Krona/OPR#3/OPR#3_NOV.html]

Javascript must be enabled to view this page.

magnitude
 75.5482523497039
 41.2187480804039
 13.2041280177001
 7.5281036918822
 1.584863935134
 0
 0
 0
 1.0780760489
 1.0780760489
 1.0780760489
 0
 0
 0
 0
 0
 0
 0
 0
 .506787886234
 .147429203268
 .147429203268
 .359358682966
 .359358682966
 0
 0
 3.2741568892486
 .0460716260213
 .0460716260213
 .0460716260213
 3.2280852632273
 3.13287056945
 3.13287056945
 0
 0
 .0952146937773
 .0952146937773
 1.3053627372703
 1.283862645127
 1.283862645127
 .178143620616
 .116714785921
 .98900423859
 0
 0
 .0061428834695
 .0061428834695
 .0061428834695
 .0153572086738
 .0153572086738
 .0153572086738
 .835432151852
 .835432151852
 .835432151852
 .835432151852
 .5282879783773
 .5282879783773
 .482216352356
 .482216352356
 .0460716260213
 .0460716260213
 3.2557282388368
 .9030038700168
 .623502672154
 0
 0
 0
 0
 .623502672154
 .623502672154
 0
 0
 .2795011978628
 0
 0
 .215000921433
 .215000921433
 .0645002764298
 .0645002764298
 0
 0
 0
 0
 2.35272436882
 2.35272436882
 2.35272436882
 2.35272436882
 0
 0
 0
 0
 0
 2.3987959948378
 .0645002764298
 .0645002764298
 .0645002764298
 .0645002764298
 0
 0
 0
 0
 2.01486577799425
 .00921432520425
 .00921432520425
 .00921432520425
 2.00565145279
 2.00565145279
 2.00565145279
 .31942994041375
 .316358498679
 .316358498679
 .316358498679
 .00307144173475
 .00307144173475
 .00307144173475
 .0215000921433
 .0215000921433
 .0215000921433
 .0215000921433
 .0215000921433
 .439216168069
 .439216168069
 .439216168069
 .439216168069
 .439216168069
 .439216168069
 1.0074328889995
 .0061428834695
 .0061428834695
 .0061428834695
 .0061428834695
 .0061428834695
 .706431598993
 .706431598993
 .528287978377
 0
 0
 .528287978377
 .528287978377
 .178143620616
 .178143620616
 .178143620616
 .294858406537
 .104429018982
 .104429018982
 .104429018982
 .104429018982
 0
 0
 0
 0
 0
 0
 0
 0
 0
 0
 0
 0
 .190429387555
 .190429387555
 .190429387555
 .190429387555
 2.4540819460658
 .0399287425518
 .0399287425518
 .0399287425518
 .0399287425518
 .0399287425518
 2.414153203514
 2.414153203514
 1.806007740033
 .774003317157
 .774003317157
 .454573376743
 .454573376743
 .577431046133
 .577431046133
 .608145463481
 .224215246637
 .224215246637
 .383930216844
 .383930216844
 .724860249401
 .724860249401
 .724860249401
 .724860249401
 .724860249401
 .724860249401
 0
 0
 0
 0
 0
 0
 0
 0
 .77400331715765
 .77400331715765
 .6880029485846
 .6480742060328
 .0767860433688
 .0767860433688
 .571288162664
 .571288162664
 .0399287425518
 .0399287425518
 .0399287425518
 .0767860433688
 .0767860433688
 .0767860433688
 .0767860433688
 .00921432520425
 .00921432520425
 .00921432520425
 .00921432520425
 .0399287425518
 .0399287425518
 .0399287425518
 .0399287425518
 .0399287425518
 0
 .0399287425518
 22.575096750459
 22.575096750459
 20.618588365419
 20.3605872597
 20.3605872597
 20.3605872597
 .258001105719
 .258001105719
 .258001105719
 1.95650838504
 1.95650838504
 1.95650838504
 1.95650838504
 34.3295042693
 34.3295042693
 34.3295042693
 34.3295042693
 34.3295042693
 34.3295042693
 34.3295042693
