# Supplementary material for: Assessment of biomass potentials of microalgal communities in open pond raceways using mass cultivation
Source: PeerJ. 2020 Jul 16;8:e9418. doi: 10.7717/peerj.9418 (PMC7369025; doi:10.7717/peerj.9418)
Supplement: Data S5 [file peerj-08-9418-s022.zip › Krona/OPR#3/OPR#3_OCT.html]

Javascript must be enabled to view this page.

magnitude
 64.2354708734157
 37.7928020214157
 14.8057092125944
 6.50139998634626
 1.12681827494316
 0
 0
 0
 .717066174964
 .717066174964
 .717066174964
 0
 0
 0
 0
 0
 0
 0
 0
 .40975209997916
 .198046848323
 .198046848323
 .208290650823
 .208290650823
 .00341460083316
 .00341460083316
 2.7828996790317
 .0785358191627
 .0785358191627
 .0785358191627
 2.704363859869
 2.68387625487
 2.68387625487
 0
 0
 .020487604999
 .020487604999
 1.4443761524284
 1.393157139931
 1.393157139931
 .266338864987
 .201461449157
 .925356825787
 0
 0
 0
 0
 0
 .0512190124974
 .0512190124974
 .0512190124974
 .836577204125
 .836577204125
 .836577204125
 .836577204125
 .310728675818
 .310728675818
 .126340230827
 .126340230827
 .184388444991
 .184388444991
 4.500443898101
 1.591203988251
 1.2770607116
 0
 0
 0
 0
 1.2770607116
 1.2770607116
 0
 0
 .314143276651
 0
 0
 .187803045824
 .187803045824
 .126340230827
 .126340230827
 0
 0
 0
 0
 2.90923990985
 2.90923990985
 2.90923990985
 2.90923990985
 0
 0
 0
 0
 0
 3.7458171139833
 .307314074985
 .307314074985
 .307314074985
 .307314074985
 0
 0
 0
 0
 2.87850850236
 0
 0
 0
 2.87850850236
 2.87850850236
 2.87850850236
 .5599945366383
 .532677729973
 .532677729973
 .532677729973
 .0273168066653
 .0273168066653
 .0273168066653
 .0580482141638
 .0580482141638
 .0580482141638
 .0580482141638
 .0580482141638
 .39950829748
 .39950829748
 .39950829748
 .39950829748
 .39950829748
 .39950829748
 3.1755787748352
 .0341460083316
 .0341460083316
 .0341460083316
 .0341460083316
 .0341460083316
 2.74875367069
 2.74875367069
 1.2565731066
 0
 0
 1.2565731066
 1.2565731066
 1.49218056409
 1.49218056409
 1.49218056409
 .3926790958136
 .20487604999
 .20487604999
 .20487604999
 .20487604999
 0
 0
 0
 0
 0
 0
 .0341460083316
 .0341460083316
 .0341460083316
 .0341460083316
 0
 0
 .153657037492
 .153657037492
 .153657037492
 .153657037492
 4.131667008125
 .12975483166
 .12975483166
 .12975483166
 .12975483166
 .12975483166
 4.001912176465
 4.001912176465
 2.386805982378
 .225363654989
 .225363654989
 1.88144505907
 1.88144505907
 .279997268319
 .279997268319
 1.615106194087
 .331216280817
 .331216280817
 1.28388991327
 1.28388991327
 .9321860274538
 .915113023288
 .915113023288
 .915113023288
 .915113023288
 .915113023288
 .0170730041658
 .0170730041658
 .0170730041658
 .0170730041658
 .0170730041658
 0
 0
 0
 .9799904391183
 .9799904391183
 .9287714266208
 .870723212457
 .221949054156
 .221949054156
 .648774158301
 .648774158301
 .0580482141638
 .0580482141638
 .0580482141638
 .040975209998
 .040975209998
 .040975209998
 .040975209998
 .0102438024995
 .0102438024995
 .0102438024995
 .0102438024995
 .32438707915
 .32438707915
 .32438707915
 .32438707915
 .32438707915
 .020487604999
 .303899474151
 13.043775182659
 13.043775182659
 12.142320562704
 11.5550092194
 11.5550092194
 11.5550092194
 .587311343304
 .587311343304
 .587311343304
 .901454619955
 .901454619955
 .901454619955
 .901454619955
 26.442668852
 26.442668852
 26.442668852
 26.442668852
 26.442668852
 26.442668852
 26.442668852
