# Supplementary material for: Assessment of biomass potentials of microalgal communities in open pond raceways using mass cultivation
Source: PeerJ. 2020 Jul 16;8:e9418. doi: 10.7717/peerj.9418 (PMC7369025; doi:10.7717/peerj.9418)
Supplement: Data S5 [file peerj-08-9418-s022.zip › Krona/OPR#3/OPR#3_SEP.html]

Javascript must be enabled to view this page.

magnitude
 71.1312887261018
 33.4671291064018
 8.94742995205794
 5.4752347920911
 1.196368029306
 0
 0
 0
 .849538209735
 .849538209735
 .849538209735
 0
 0
 0
 0
 0
 0
 0
 0
 .346829819571
 .144187677799
 .144187677799
 .202642141772
 .202642141772
 0
 0
 1.4808464206339
 .0155878570594
 .0155878570594
 .0155878570594
 1.4652585635745
 1.45356767078
 1.45356767078
 0
 0
 .0116908927945
 .0116908927945
 2.0575971318316
 1.97576088227
 1.97576088227
 .444253926191
 .151981606329
 1.37952534975
 0
 0
 0
 0
 0
 .0818362495616
 .0818362495616
 .0818362495616
 .506605354429
 .506605354429
 .506605354429
 .506605354429
 .2338178558906
 .2338178558906
 .175363391918
 .175363391918
 .0584544639726
 .0584544639726
 2.0692880246251
 .3507267838351
 .303963212657
 0
 0
 0
 0
 .303963212657
 .303963212657
 0
 0
 .0467635711781
 0
 0
 .0272787498539
 .0272787498539
 .0194848213242
 .0194848213242
 0
 0
 0
 0
 1.71856124079
 1.71856124079
 1.71856124079
 1.71856124079
 0
 0
 0
 0
 0
 .98982892326874
 .541678032812
 .541678032812
 .541678032812
 .541678032812
 0
 0
 0
 0
 .0974241066209
 0
 0
 0
 .0974241066209
 .0974241066209
 .0974241066209
 .35072678383584
 .346829819571
 .346829819571
 .346829819571
 .00389696426484
 .00389696426484
 .00389696426484
 .413078212073
 .413078212073
 .413078212073
 .413078212073
 .413078212073
 .381902497954
 .381902497954
 .381902497954
 .381902497954
 .381902497954
 .381902497954
 8.6824363820465
 .210436070301
 .210436070301
 .210436070301
 .210436070301
 .210436070301
 3.105880519072
 3.105880519072
 2.18619695257
 0
 0
 2.18619695257
 2.18619695257
 .919683566502
 .919683566502
 .919683566502
 5.3661197926735
 2.54082070067
 2.54082070067
 2.54082070067
 2.54082070067
 0
 0
 0
 0
 0
 0
 .0350726783835
 .0350726783835
 .0350726783835
 .0350726783835
 0
 0
 2.79022641362
 2.79022641362
 2.79022641362
 2.79022641362
 5.2647987217923
 .689762674876
 .689762674876
 .689762674876
 .689762674876
 .689762674876
 4.5750360469163
 4.5750360469163
 2.5875842718503
 .0896301780913
 .0896301780913
 2.35376641596
 2.35376641596
 .144187677799
 .144187677799
 1.987451775066
 .335138926776
 .335138926776
 1.65231284829
 1.65231284829
 .8846108881179
 .833950352675
 .833950352675
 .833950352675
 .833950352675
 .833950352675
 .0506605354429
 .0506605354429
 .0506605354429
 .0506605354429
 .0506605354429
 0
 0
 0
 .8456412454701
 .8456412454701
 .7793928529679
 .705350531936
 .12080589221
 .12080589221
 .584544639726
 .584544639726
 .0740423210319
 .0740423210319
 .0740423210319
 .0662483925022
 .0662483925022
 .0662483925022
 .0662483925022
 0
 0
 0
 0
 1.2859982073981
 1.2859982073981
 1.2859982073981
 1.2859982073981
 1.2859982073981
 .0467635711781
 1.23923463622
 7.174311211565
 7.174311211565
 5.635010326955
 4.80105997428
 4.80105997428
 4.80105997428
 .833950352675
 .833950352675
 .833950352675
 1.53930088461
 1.53930088461
 1.53930088461
 1.53930088461
 37.6641596197
 37.6641596197
 37.6641596197
 37.6641596197
 37.6641596197
 37.6641596197
 37.6641596197
