# Supplementary material for: Assessment of biomass potentials of microalgal communities in open pond raceways using mass cultivation
Source: PeerJ. 2020 Jul 16;8:e9418. doi: 10.7717/peerj.9418 (PMC7369025; doi:10.7717/peerj.9418)
Supplement: Data S5 [file peerj-08-9418-s022.zip › Krona/OPR#3.html]

Javascript must be enabled to view this page.

magnitude
 73.4488727226963
 49.9661972756238
 20.4581194433229
 6.16289601161775
 .758531994891764
 4.89145286745813E-02
 4.89145286745813E-02
 4.89145286745813E-02
 .415448271674638
 .414520145958438
 .414520145958438
 .0009281257162
 .0009281257162
 8.12649544828625E-02
 8.12649544828625E-02
 8.12649544828625E-02
 .004571283897825
 .004571283897825
 .004571283897825
 .208332956161858
 .063669426734125
 .063669426734125
 .142934078229388
 .142934078229388
 .001729451198345
 .001729451198345
 2.24832887278489
 2.05749724055875E-02
 2.05749724055875E-02
 2.05749724055875E-02
 2.2277539003793
 .99316569061285
 .99316569061285
 1.22952538826737E-02
 1.22952538826737E-02
 1.22229295588378
 1.22229295588378
 1.51682506843324
 1.32512934446663
 1.32512934446663
 .3191352793696
 .192087103621825
 .725410023131375
 1.75130425065125E-02
 7.09838958373187E-02
 1.4711348795075E-03
 1.4711348795075E-03
 1.4711348795075E-03
 .1902245890871
 .1902245890871
 .1902245890871
 1.50510576174713
 1.50510576174713
 1.50510576174713
 1.50510576174713
 .134104313760738
 .134104313760738
 .097989996887625
 .097989996887625
 3.61143168731125E-02
 3.61143168731125E-02
 3.29533753609611
 1.68268832805011
 1.46027666680136
 .00105491166873
 .00105491166873
 .996787774591375
 .996787774591375
 .461730706095438
 .461730706095438
 .00070327444582
 .00070327444582
 .222411661248746
 .131982521475788
 .131982521475788
 6.20507852660875E-02
 6.20507852660875E-02
 2.83783545068713E-02
 2.83783545068713E-02
 0
 0
 0
 0
 1.612649208046
 1.612649208046
 1.612649208046
 1.612649208046
 3.74129663260375E-03
 3.74129663260375E-03
 3.74129663260375E-03
 3.74129663260375E-03
 3.74129663260375E-03
 10.7114987524017
 .203078006719446
 .203078006719446
 .203078006719446
 .203078006719446
 4.42926547964375E-03
 4.42926547964375E-03
 4.42926547964375E-03
 4.42926547964375E-03
 10.1942110591662
 1.53918114048813E-02
 1.53918114048813E-02
 1.53918114048813E-02
 10.1788192477614
 10.1788192477614
 10.1788192477614
 .309780421036397
 .27889094655135
 .27889094655135
 .27889094655135
 3.08894744850475E-02
 3.08894744850475E-02
 3.08894744850475E-02
 .284645846574702
 .284645846574702
 .284645846574702
 .284645846574702
 .284645846574702
 .217919625103838
 .217919625103838
 .217919625103838
 .217919625103838
 .217919625103838
 .217919625103838
 11.145867878387
 2.31013903050855
 2.31013903050855
 2.31013903050855
 2.31013903050855
 2.31013903050855
 5.93249563523749
 5.93249563523749
 5.32319503774605
 .03392413071406
 .03392413071406
 5.28927090703199
 5.28927090703199
 .609300597491445
 .609300597491445
 .609300597491445
 2.90323321264101
 .742353800273672
 .599237450549513
 .549770016079063
 .549770016079063
 .04946743447045
 .04946743447045
 .14311634972416
 .14311634972416
 .00035163722291
 .14276471250125
 8.98183815908375E-03
 8.98183815908375E-03
 8.98183815908375E-03
 8.98183815908375E-03
 0
 0
 2.15189757420825
 2.15189757420825
 2.15189757420825
 2.15189757420825
 5.17507998506277
 2.25671086424023
 2.25671086424023
 2.25671086424023
 2.25671086424023
 2.25671086424023
 2.91836912082255
 2.91836912082255
 2.36119150869026
 .376772529799288
 .376772529799288
 .6982047635266
 .6982047635266
 1.28621421536438
 1.28621421536438
 .557177612132284
 .123742180673399
 .123742180673399
 .433435431458885
 .433435431458885
 1.01010599180446
 .8556147330628
 .8556147330628
 .8556147330628
 .8556147330628
 .8556147330628
 .154491258741661
 .154491258741661
 .153439230344125
 .153439230344125
 .153439230344125
 1.05202839753625E-03
 1.05202839753625E-03
 1.05202839753625E-03
 .544739305950161
 .544739305950161
 .379269118119743
 .341143114548167
 .075275555429355
 .075275555429355
 .265867559118812
 .265867559118812
 .038126003571575
 .038126003571575
 .038126003571575
 6.11035200649625E-02
 6.11035200649625E-02
 6.11035200649625E-02
 6.11035200649625E-02
 .104366667765456
 .104366667765456
 .104366667765456
 .104366667765456
 1.38331923418635
 1.38331923418635
 1.38331923418635
 1.38331923418635
 1.38331923418635
 .392995871076625
 .990323363109725
 10.0310458118063
 10.0310458118063
 6.15354897577804
 5.59949316653901
 5.59949316653901
 5.59949316653901
 .554055809239025
 .554055809239025
 .554055809239025
 3.87749683602825
 3.87749683602825
 3.87749683602825
 3.87749683602825
 23.4826754470725
 23.4826754470725
 23.4826754470725
 23.4826754470725
 23.4826754470725
 23.4826754470725
 23.4826754470725
